# Findzx: an automated pipeline for detecting and visualising sex chromosomes using whole-genome sequencing data

**DOI:** 10.1186/s12864-022-08432-9

**Published:** 2022-04-27

**Authors:** Hanna Sigeman, Bella Sinclair, Bengt Hansson

**Affiliations:** Department of Biology, Lund University, Ecology Building, 223 62 Lund, Sweden

**Keywords:** Sex chromosomes, Snakemake, Paired-end, Genomics

## Abstract

**Background:**

Sex chromosomes have evolved numerous times, as revealed by recent genomic studies. However, large gaps in our knowledge of sex chromosome diversity across the tree of life remain. Filling these gaps, through the study of novel species, is crucial for improved understanding of why and how sex chromosomes evolve. Characterization of sex chromosomes in already well-studied organisms is also important to avoid misinterpretations of population genomic patterns caused by undetected sex chromosome variation.

**Results:**

Here we present findZX, an automated Snakemake-based computational pipeline for detecting and visualizing sex chromosomes through differences in genome coverage and heterozygosity between any number of males and females. A main feature of the pipeline is the option to perform a genome coordinate liftover to a reference genome of another species. This allows users to inspect sex-linked regions over larger contiguous chromosome regions, while also providing important between-species synteny information. To demonstrate its effectiveness, we applied findZX to publicly available genomic data from species belonging to widely different taxonomic groups (mammals, birds, reptiles, and fish), with sex chromosome systems of different ages, sizes, and levels of differentiation. We also demonstrate that the liftover method is robust over large phylogenetic distances (> 80 million years of evolution).

**Conclusions:**

With findZX we provide an easy-to-use and highly effective tool for identification of sex chromosomes. The pipeline is compatible with both Linux and MacOS systems, and scalable to suit different computational platforms.

**Supplementary Information:**

The online version contains supplementary material available at 10.1186/s12864-022-08432-9.

## Background

Sex determination, the process by which sexually reproducing organisms initiate the developmental program to become male, female, or hermaphroditic, is remarkably diverse [1–3]. The mechanism that triggers the sex determination program can be either genetic, environmental, or a combination of both. In many animals and some plants this process is genetically controlled by sex chromosomes, where either males (XY systems) or females (ZW systems) are heterogametic, i.e., carry two different sex chromosomes. Sex chromosomes have evolved many times, both de novo in organisms without genetic sex determination and through turnovers of already existing sex chromosome systems [1, 30, 31]. There have also been numerous translocations of autosomes to existing sex chromosomes (forming “neo-sex chromosomes”), which may result in variable gene content between the sex chromosomes of different, closely related species. Despite recent efforts in identifying and characterizing sex chromosomes across a broad range of taxonomic groups (e.g., [12, 14, 23]), we are likely still missing much of the existing sex chromosome diversity. Filling these knowledge gaps is important for an improved understanding of how and why sex chromosomes evolve, and to avoid misinterpretations of population genomic data caused by undetected sex chromosome diversity.

Sex chromosomes can be identified using cytogenetic methods, where sex-specific karyotype differences (in chromosome number and/or size) can reveal the sex chromosome pair. These methods may, however, fail to reveal homomorphic sex chromosomes (as they are of similar size) and provide imprecise information on gene content and homologies to other species. Sex chromosomes can also be identified by contrasting genomic (or transcriptomic) data from males and females (e.g., [33]). This is because X and Y (or Z and W) are expected to evolve genetic differences as a consequence of recombination suppression. Such “sex-linked” regions may be detected through a range of genome signatures, including sex differences in allele segregation patterns, gene expression, heterozygosity, or genome coverage (reviewed in [22]). Different data types, sampling strategies and computational methods may be suitable for detecting sex chromosomes of variable degrees of differentiation, with homomorphic sex chromosomes requiring more carefully designed computational methods and sampling strategies.

Here we present findZX, a Snakemake-based [17] pipeline that identifies sex chromosomes, or more specifically the non-recombining part of the sex chromosomes, using whole-genome sequencing (WGS) paired-end data from a flexible number of male and female samples. In essence, findZX scans across windows of a reference genome for two genomic signatures of sex-linkage: sex differences in (i) genome coverage and (ii) heterozygosity. The combined analysis of these measurements is a powerful and widely used approach for identifying sex chromosome systems of varying degrees of differentiation [22, 33]. The pipeline can be applied to fragmented (scaffold-level) genome assemblies but also includes the option to perform a “liftover” to a more contiguous reference genome of another species. The benefit of this i s twofold: it allows users to inspect sex-linked regions over larger contiguous chromosome regions, while also providing detailed synteny information between species. FindZX is available on GitHub (https://github.com/hsigeman/findZX).

Several other programs and computational resources for detecting sex-linked genomic regions have been published. Among these are SEX-DETector [20] (which uses expression data from a pedigree of samples), discoverY [26] (which finds sex-limited scaffolds through comparisons of reference genomes from a homogametic and heterogametic sample) and RADSex [11] (which uses restriction site–associated DNA sequencing (RAD-seq) data from several males and females). WGS is now a standard form of sequencing, which is being increasingly used to characterize sex chromosomes (e.g., [4, 25, 34]). Recently, a method to identify sex-linked scaffolds from WGS data using genome coverage was published (SATC [21]). FindZX is, however, to our knowledge the first computational resource that uses WGS data to detect sex chromosomes using the highly effective combined approach of heterozygosity and genome coverage. It is also, to our knowledge, the first program that includes an option for automatic genome-coordinate liftover. 

**Fig. 1:**
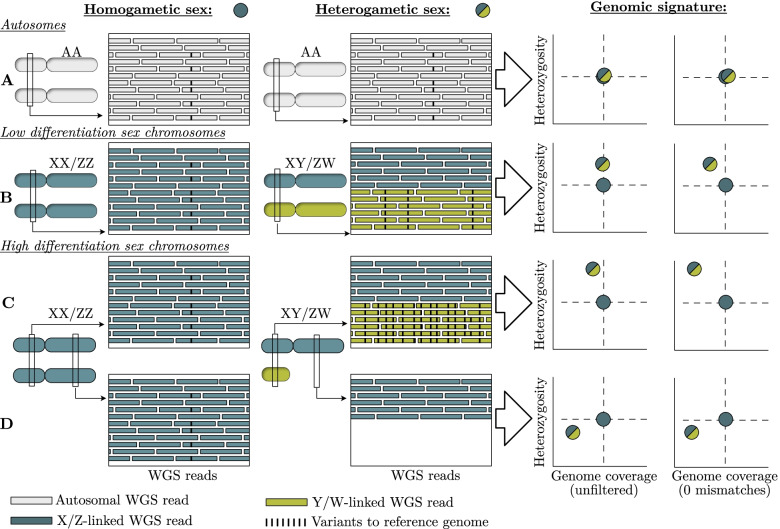
Sex-linked genomic regions of varying stages of differentiation can be detected through differences in genome coverage and heterozygosity between WGS reads of males and females aligned to a homogametic (XX/ZZ) reference genome. Reads are coloured according to chromosome origin (“autosomal”, “X/Z-linked” or “Y/W-linked”). Genome coverage is calculated as the number of WGS reads aligning to a specific genomic region. The black bars within the WGS reads represent genetic variants compared to the reference allele (i.e., heterozygous sites). (**A**) Genome coverage and heterozygosity is expected to be similar between sexes on autosomes. (**B**) Sex chromosomes of low differentiation are characterized by higher heterozygosity in the heterogametic sex. They often display equal genome coverage between sexes when allowing mismatching reads to map to the reference genome but pronounced coverage differences when restricting the number of allowed mismatches. (**C**,**D**) Highly differentiated sex chromosomes are expected to have either equal genome covera ge between sexes when allowing mismatches, or lower genome coverage in the heterogametic sex if the sequence divergence is large enough to prevent successful alignment of reads to the reference genome. When restricting the allowed number of mismatches, we expect significantly lower genome coverage in the heterogametic sex. If the genomic region has a completely degenerated sex-limited chromosome copy, we expect lower genome coverage in the heterogametic sex regardless of the number of allowed mismatches. Highly differentiated sex chromosomes will have either (**C**) very high or (**D**) lower heterozygosity in the heterogametic sex depending on mapping success, level of Y/W degeneration, and level of genetic variation on X/Z

FindZX aligns paired-end WGS reads of female and male samples to a reference genome constructed from the homogametic sex (female XX or male ZZ) and detects chromosomal regions with sex differences in genome coverage and heterozygosity by contrasting them to the genome-wide (autosomal) pattern characterised by no such sex differences (Fig. 1a). The sex-specific signature depends on the level of sex chromosome differentiation and Y/W degeneration because reads from the sex-limited chromosome (Y/W) may or may not successfully align to the homogametic (XX/ZZ) reference genome. In general, species with little differentiation between the sex chromosome copies (Fig. 1b) are expected to show weaker sex-specific signals than species with highly differentiated sex chromosomes (Fig. 1c,d). Restricting the number of allowed mismatches between the reference genome and aligned reads when attempting to identify sex chromosomes is reco mmended, as this prevents reads from the sex-limit ed chromosome aligning to its gametologous chromosome copy and therefore increase the sex difference in genome coverage. For low differentiation (homomorphic) sex chromosome systems, t he clearest geno mic signature will be heterozygosity as many reads from the sex-limited chromosome (Y/W) will align (to the X/Z), while genome coverage may only re veal sex differences when strongly rest ricting the number of allowed mismatches (Fig. 1b). For high differentiation (heteromorphic) sex chromosome systems, the clearest genomic signature will be cove rage differences, especially when r estricting the number of allowed mismatches (Fig. 1c,d), while heterozygosity may sometimes be skewed towards the heterogametic sex if reads from the sex-limited chromosomes do align (Fig. 1c) or towards the homogametic sex if they do not (due to genetic variation on X/Z resulting in heterozygosity only in homogametic individuals) (Fig. 1d). Since findZX uses a homogametic reference genome as input, it is designed to identify X and Z chromosomes/scaffolds rather than Y and W chromosomes. However, the output will reveal useful information about the size of the Y or W chromosome, and the level of X-to-Y, or Z-to-W, divergence.

We applied the pipeline to published data from species of various taxonomic clades and sex chromosome systems to demonstrate its effectiveness in both identifying the sex chromosomes and finding homology to other species.

### Implementation

**Fig. 2:**
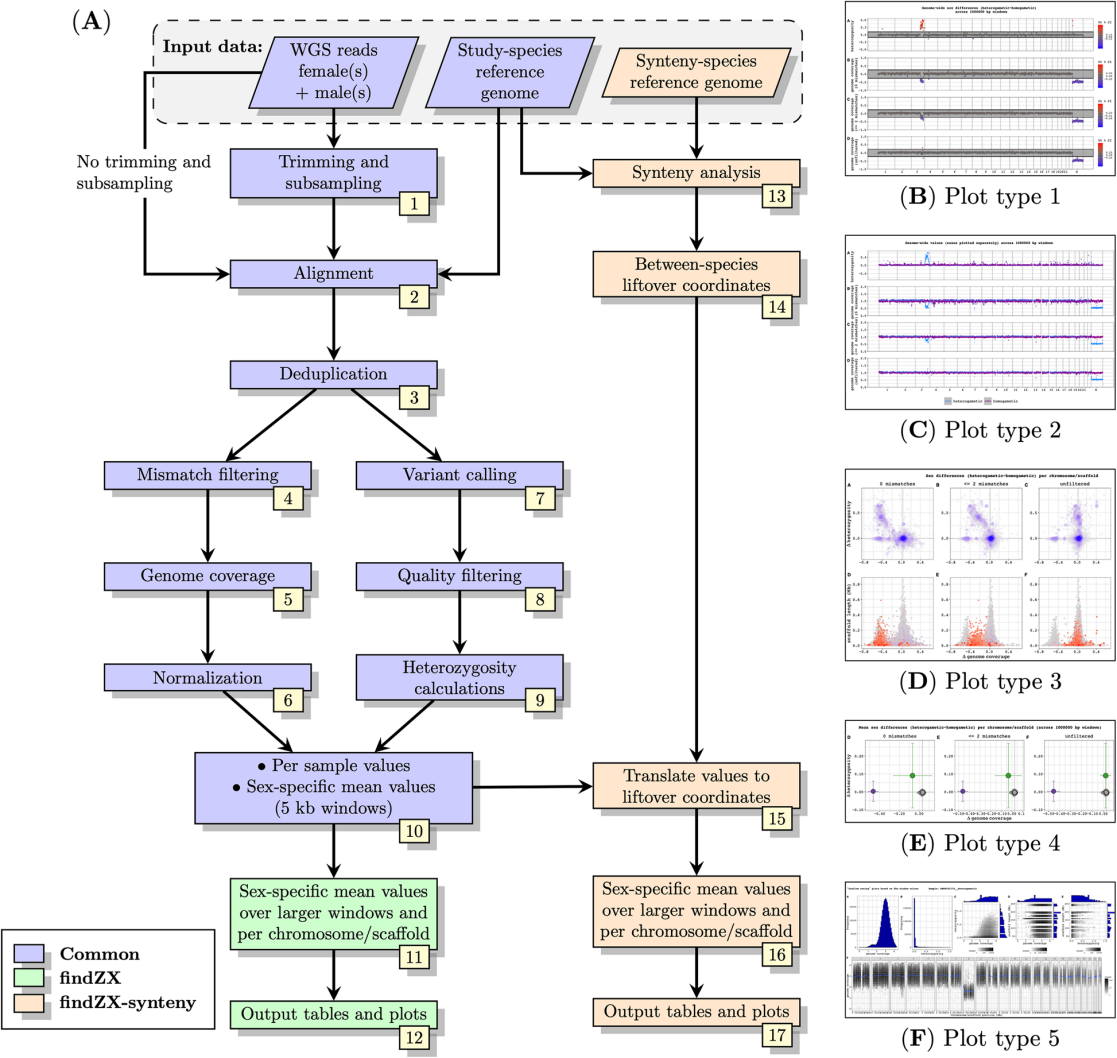
Flowchart of the main computational steps in the findZX/findZX-synteny pipeline (**A**), and miniatures of the five output plot types (**B**-**F**). **A** The flowchart boxes in blue are common for both findZX and findZX-synteny. The green boxes are specific for findZX and orange boxes are specific for findZX-synteny. Parallelograms (top row) represent input data, and rectangles represent computational steps and output. See Main text and Supplementary Methods (Additional File 1) for details. Plot type 1 (**B**; see also Fig. 3) and 2 (**C**; see also Supplementary Fig. 2, Additional File 1) show genome-wide heterozygosity and genome coverage values based on means across genome windows (here 1 Mb). Plot type 1 (**B**) shows sex differences and 2 (**C**) shows values for each sex separately. Plot type 3 (**D**; see also Fig. 4) shows heterozygosity and genome coverage values for each chromosome/scaffold, as well as chromosome/scaffold length. Plot type 4 (**E**; see also Fig. 5) shows mean ± SD sex differences (calculated from genome windows, here 1 Mb) for each chromosome/scaffold. Here we invoked the option to highlight certain chromosomes/scaffolds (specified in the configuration file; see Main text). Plot type 5 (**F**; see also Supplementary Fig. 3 and 4, Additional File 1) shows heterozygosity and genome coverage profiles for each studied individual and can be used to confirm that samples have been correctly sexed

FindZX identifies sex-linked genomic regions using WGS reads from samples of different sexes, with a minimum input of one individual per sex and a reference genome constructed from a homogametic individual (“study-species reference genome”). When no reference genome is available for the studied species (as would be the case for most studies), a scaffold-level based assembly sufficient for the pipeline can easily be constructed de novo using the short-read data from one of the homogametic samples and standard assembly programs (see example on how to do this on the findZX GitHub page). Figure 2 shows the main computational steps (Fig. 2a) and examples of the output plot types (Fig. 2b-f; “plot type 1–5”; see also Supplementary Fig. 1, Additional File 1, for larger versions) of findZX. The pipeline can be run using either of two snakefiles: findZX or findZX-synteny. Input data and computational steps in blue boxes (Fig. 2a; Steps 1–10) are common for both run modes. Computational steps in green are specific for findZX (Steps 11–12), whereas input data (a reference genome from a second species; “synteny-species reference genome”) and computational steps in orange (Steps 13–17) are specific for findZX-synteny. The aim of this section is to provide a conceptual overview of the computational steps and output generated by findZX. For details about the software and settings used by findZX in each computational step, see Supplementary Methods, Additional File 1.

The first steps, trimming and subsampling of WGS reads (Fig. 2a: Step 1), are optional. If the input reads are already trimmed for adaptors and bad quality sequences, this step can be skipped. The subsampling step may be used if the WGS files are unnecessarily large, in which case subsampling will reduce the pipeline run time. The reads are then aligned to the study-species reference genome (Step 2) and duplicate reads are removed (Step 3). From these deduplicated output BAM files (referred to as “unfiltered”), reads with different (and modifiable) number of mismatches to the reference genome are removed, and the remaining reads are written to new BAM files (Step 4). The default mismatch settings, which were used for all analyses in this paper, are: i) no filtering (“unfiltered”; see above), ii) intermediate filtering (≤ 2 mismatches allowed), and iii) strict filtering (0 mismatches allowed). Genome coverage is then calculated for each sample from these BAM files across 5 kb windows (Step 5). Inspecting the results from different mismatch settings is useful since the optimal mismatch threshold may differ between species and sex chromosome systems. Comparisons of different mismatch settings may also reveal important information about the level of sex chromosome differentiation, and the level of Y/W chromosome degeneration. Extreme genome coverage outliers are masked before the genome coverage values are being normalized between samples (Step 6). Variants are called from the “unfiltered” BAM file (Step 7), followed by quality filtering (Step 8) and heterozygosity calculations across 5 kb windows for each sample (Step 9). Sex-specific genome coverage and heterozygosity mean values (if more than one sample per sex is used) is then calculated for each 5 kb window (Step 10). If the pipeline is run with the findZX snakefile, mean values will be calculated over larger genome windows (Step 11), and output plots and tables will be generated (Step 12).

If the pipeline is run with findZX-synteny, a synteny analysis is performed between the two reference genomes (Step 13) and the genome coordinates from the study-species reference genome are lifted over to the reference genome of a second species (Step 14). This option is recommended if the study-species reference genome is below chromosome-level, and/or to establish homology between sex chromosome systems (discussed below). The sex-specific genome coverage and heterozygosity values (Step 10) will then be translated to these liftover coordinates in the second species (Step 15). Lastly, the mean of these translated values will be calculated over larger genome windows (Step 16), and output plots and tables will be generated (Step 17). 

If a list of chromosomes/scaffolds is provided as input to the pipeline, the plots will only show these ones. Similarly, certain chromosomes/scaffolds can be highlighted in plot type 4 if specified. All plotting steps are quick to re-run (e.g., with different sets of chromosomes/scaffolds and highlighting options) and do not require reanalysis of previous steps.

Four of the five output plot types (1–4; Fig. 2b-e) are based on data from Step 11 (findZX) or 16 (findZX-synteny). In the last plot type (5; Fig. 2f), genome coverage and heterozygosity values are plotted for each individual and mismatch setting separately (based on the data from Step 10 for findZX, Step 15 for findZX-synteny). Plot type 5 may be informative in confirming the sexing of included samples, and to inspect the genome coverage depth for each sample. Note, however, that for low differentiation sex chromosome systems, or systems where the sex chromosomes are extremely small, there may not be clear differences between sexes in these plots.

If the pipeline is run with one sample per sex together with a de novo-assembled study-species reference genome constructed from the homogametic sample, and if the species is highly heterozygous, the sex-specific signatures may be suboptimal and the results not clear. To circumvent this problem, the pipeline contains an option to construct a “consensus genome”, by incorporating genetic variants found in the two samples into the original study-species reference genome (Fig. 2a; using the VCF file from Step 8; see also Supplementary Methods, Additional File 1). Running the pipeline again using this consensus reference genome instead of the original one has proven effective in a previous study, as it results in an autosome-wide equal mapping success between samples (for details see [28]). Note, however, that very high genome-wide heterozygosity levels (≥ 1%) may lead to failure to reveal the sex chromosomes (see below). Per-sample heterozygosity values, genome assembly statistics, and the proportion of genome windows that was successfully lifted over to a synteny-species reference genome (if opted for), are generated automatically by findZX.

All findZX output plots are multi-page PDF files, where the last page contains data paths to the output tables used to create each plot. All output plots and selected output tables (outlier values as visualized in plot type 1, genome assembly statistics, heterozygosity values and synteny-analysis statistics) can also be summarized in an interactive HTML report, with additional descriptions of each plot type.

## Results

### Datasets for pipeline evaluation

We evaluated the pipeline by analysing data from five species with previously identified sex chromosomes (Supplementary Methods, Additional File 1). These species were selected to represent a broad range of sex chromosome systems in terms of XY (or ZW) differentiation, size, and age. We included the platypus (*Ornithorhynchus anatinus*), a species with multiple highly differentiated sex chromosomes (X_1-5_Y_1-5_ [13]). We also included two species with “neo-sex chromosomes” formed through translocations between the ancestral sex chromosomes and (parts of) autosomes. In these species, Eurasian skylark (*Alauda arvensis*; neo-ZW [7, 27]) and mantled howler monkey (*Alouatta palliata*; neo-XY [19, 29]), the added (formerly) autosomal regions are characterized by less sex chromosome differentiation than the old, ancestral part. We also included the guppy (*Poecilia reticulata),* a species with an extremely low differentiated XY-system [16, 34], and the central bearded dragon (*Pogona vitticeps*) which has a micro-ZW sex chromosome system in which identification of the sex chromosome sequence as well as homology to other species has proven challenging (but see [6]). Lastly, we demonstrate the usefulness of the pipeline for identifying other divergent haplotype blocks using genomic data from different morphs of the ruff (*Calidris pugnax*), a bird species where male phenotypes (plumage colouration and behaviour) are determined by an inversion polymorphism [18] (Supplementary Methods, Additional File 1).

Accession numbers for all WGS data are provided in Supplementary Table 1 (Additional File 2), accession numbers for all reference genomes used are in Supplementary Table 2 (Additional File 3), and configuration files used to run each analysis is listed in Supplementary Table 3 (Additional File 4). The number of samples used for each species ranged between 1 and 3 per sex, except for guppy where we ran the analyses with both a large (*n* = 23) and small (*n *= 2) number of samples. For species with available chromosome-level assemblies (platypus and guppy), we ran the pipeline without a synteny-species (findZX). All four remaining species (mantled howler monkey, Eurasian skylark, central bearded dragon, and ruff) were run with one or several synteny- species (findZ X-synteny), and some also without a synteny-species (findZX). Supplementary Table 3 (Additional File 4) contains information on the number of samples used for each analysis, if the pipeline was run with (findZX-synteny) or without (findZX) a synteny-species reference genome, and what chromosomes/scaffolds were expected to be sex-linked/polymorphic (details regarding this are also described in the Supplementary Methods, Additional File 1). Assembly statistics of the genomes used in this study are reported in Supplementary Tables 2 (Additional File 3), and synteny-analysis statistics are in Supplementary Table 3 (Additional File 4). Per-sample genome coverage values for all BAM files (Fig. 2a; Steps 3 and 4) as well as the genome-wide heterozygosity percentage (based on the “unfiltered” BAM files, see Fig. 2a; Step 9) are reported in Supplementary Table   4 (Additional File 5). These metrics may be used as comparisons to values from novel analyses. 

### Example use of findZX and findZX-synteny: mantled howler monkey

**Fig. 3:**
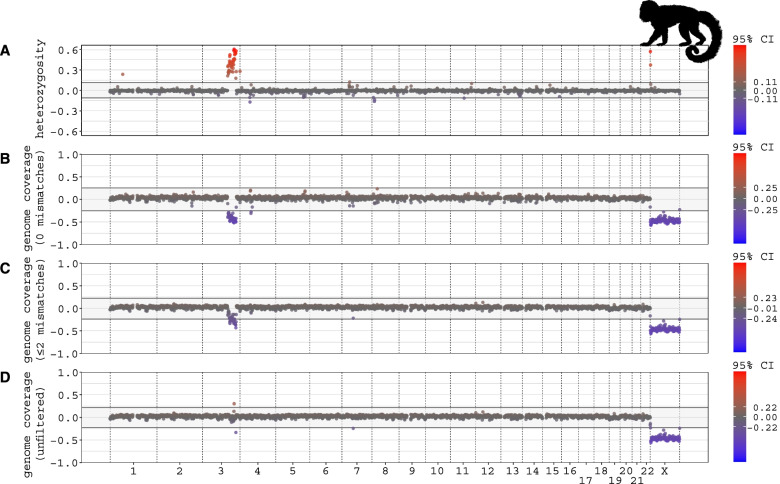
Sex differences in genome coverage and heterozygosity values (1 Mb windows) for the mantled howler monkeys, plotted along chromosome positions in the human genome. The four rows show: (**A**) heterozygosity, and genome coverage with (**B**) strict filtering (0 mismatches allowed), (**C**) intermediate filtering (≤ 2 mismatches) and (**D**) no filtering of mapped reads (“unfiltered”). The grey background marks the 95% confidence intervals (CI), and data points outside these values are red (if they are higher) or blue (if they are lower). All outlier values are reported as separate data tables in the pipeline output. The data reveals that chromosome X and a part of chromosome 3 are sex-linked in this species. The same data is also plotted for each sex separately (see Supplementary Fig. 2, Additional File 1). The silhouette was downloaded from phylopic.org

**Fig. 4:**
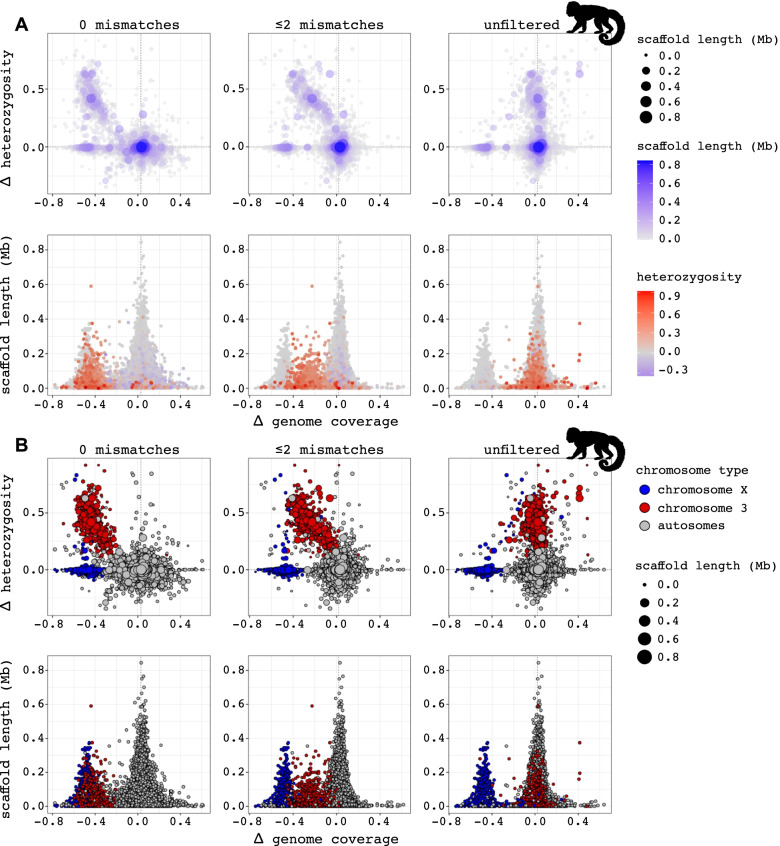
Per scaffold difference in heterozygosity and genome coverage between male and female mantled howler monkeys, with scaffold lengths indicated by symbol colour and/or size (top row in **A** and **B**) or plotted on the y-axis (bottom row in **A** and **B**). Genome coverage was calculated for three different mismatch filtering stringencies of mapped reads (left, mid, right panels: strict (0 mismatches), intermediate (≤ 2 mismatches) and no filtering (“unfiltered”)). The left-side panels show three separate clusters of scaffolds, corresponding to the expected patterns of autosomes, and sex chromosome regions of low and high differentiation, respectively. (**A**) This plot was generated through the pipeline directly, with the findZX option (i.e., not using a synteny-species reference genome). (**B**) The underlying data in this plot is identical to Fig. 4a but coloured differently to facilitate interpretation of Fig. 4a (see Main text). In this plot, the data points are coloured blue if the scaffolds aligned to the human X chromosome and red if the scaffolds aligned to the sex-linked region of chromosome 3 (134–178 Mb; Fig. 3). All other scaffolds are coloured grey. The silhouette was downloaded from phylopic.org

**Fig. 5:**
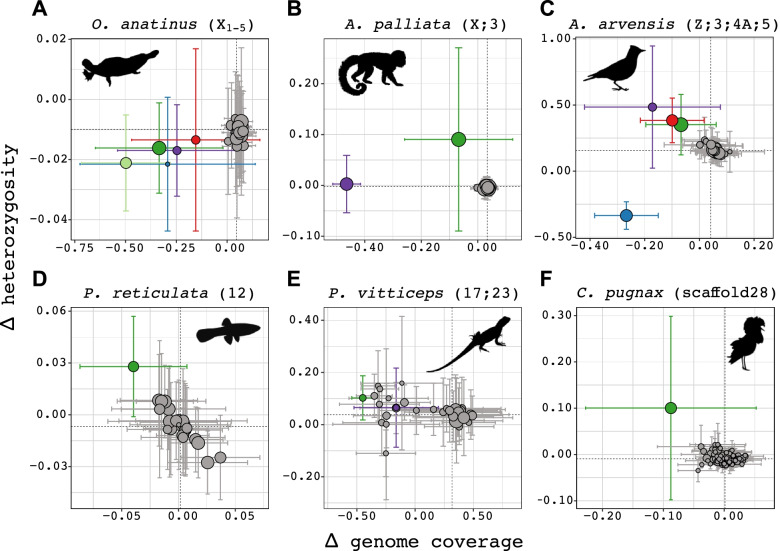
Genome coverage and heterozygosity values (mean ± standard deviation across 1 Mb windows) per chromosome or scaffold for all studied species. Dashed lines mark the genome-wide median across all 1 Mb windows. (**A-F**) The chromosome/scaffold names in parentheses next to species names are the expected sex chromosomes/inversion polymorphism scaffolds as described in the Main text and Supplementary Information (Additional File 1). These chromosomes/scaffolds are coloured differently from other chromosomes/scaffolds (which are grey). The sizes of the data points reflect the chromosome/scaffold length. Each of these plots constitutes one of 6 panels in Supplementary Figs. 5–13 (Additional File 1; shown here are the panels based on 0 mismatches, except for Eurasian skylark (*A. arvensis*) for which we show the panel based on ≤ 2 mismatches), which also have a colour legend for each species. These panels are based on pipeline runs using all samples listed for each species in Supplementary Table 1 (Additional File 2). All species except for mantled howler monkey (*A. palliata*), Eurasian skylark and central bearded dragon (*P. vitticeps*) were run using findZX. The mantled howler monkey was run, as described previously, with human (*H. sapiens*) as a synteny-species. Eurasian skylark was run with zebra finch (*T. guttata*) as a synteny-species, and for central bearded dragon we used the chicken (*G. gallus*) as a synteny-species. Silhouettes of animals were downloaded from phylopic.org (credits in Supplementary Information; Additional File 1)

Here, we describe an example output from the analysis of one of the studied species, the mantled howler monkey. Previous studies have shown that this species has a neo-sex chromosome system, formed through one or two autosome-to-Y translocations [19, 29]. In a karyotype study including other *Alouatta* species, chromosome regions homologous to human chromosomes 3 and 15 were found to have translocated to the ancestral Y chromosome [5]. This species was chosen as an example output because its neo-sex chromosome system allows us to show both what highly differentiated regions (ancestral X; cf. Fig. 1d) and more recently added regions of lower differentiation (translocated formerly autosomal regions; cf. Fig. 1b) look like in the pipeline output. We ran the pipeline using WGS data from 2 homogametic (XX) females and 2 heterogametic (XY) males, together with a newly published reference genome of this species [35] (Supplementary Table 2, Additional File 3), and both with and without the use of the human genome as a “synteny-species”. Plot types 1 (Fig. 3), 3 (Fig. 4a), and 4 (Fig. 5c) are shown here in the Main text. Plot types 2 (Supplementary Fig. 2) and 5 (Supplementary Figs. 3 and 4) are in the Supplementary Information (Additional File 1). The HTML report file generated from the “findZX-synteny” analysis is provided as Additional File 6 (“Alouatta palliata findZX-synteny HTML report”).

The reference genome of the mantled howler monkey consists of 43,519 scaffolds > 10 kb and has a scaffold N50 value of 72 kb, meaning that scaffolds covering half the genome (1.5 Gb of the total size ~ 3 Gb) are shorter than 72 kb (Supplementary Table 2, Additional File 3). Running the pipeline using a reference genome with this level of fragmentation is fine in principle (shown in Fig. 4), but the use of a synteny-species (i.e., with findZX-synteny) with higher contiguity can add valuable information. Importantly, running an analysis using a synteny-species with high contiguity (human reference genome N50 value: 156 Mb; Supplementary Table 2, Additional File 3) is likely to make distinctions between fully sex-linked and autosomal regions clearer. This is because the chromosome coordinate anchoring method will produce mean values from all small scaffolds (across 5 kb genome windows) that belong to the same genome region (e.g., within each 1 Mb window in the synteny-species reference genome). The synteny analysis successfully anchored 78% of the 5 kb windows in the mantled howler monkey to the human reference genome (Supplementary Table 3 , Additional File 4). 

The genome-wide plot types 1 (Fig. 3; sex differences) and 2 (Supplementary Fig. 2, Additional File 1; values plotted for each sex separately) show that genomic regions in the mantled howler monkey with synteny to human chromosome X (i.e., the ancestral therian mammalian sex chromosome) and chromosome 3 have pronounced sex differences in heterozygosity and genome coverage compared to the rest of the chromosomes (Fig. 3). The entire chromosome X (0–156 Mb) is characterized by markedly lower genome coverage in the heterogametic sex (male) compared to the homogametic sex (female), regardless of the number of allowed mismatches, concordant with the expected patterns from a heavily degenerated ancestral Y chromosome (homologous to chromosome X; Fig. 3). Chromosome 3 shows characteristics of a younger and less degenerated sex-linked genomic region across parts of its length (134–178 Mb), as the genome coverage is reduced only when restricting the number of allowed mismatches and there is a clear excess of heterozygosity in the heterogametic sex compared to the homogametic sex (Fig. 3).

The grey area of plot type 1 marks the 95% confidence intervals from the genome-wide mean value for each metric (Fig. 3a-d), and windows with values exceeding these thresholds are reported as separate “outlier tables”. These outlier tables, which are added to the HTML report, can be used to estimate the genomic range of candidate sex-linked regions and candidate pseudoautosomal (PAR) boundaries. Plot type 2 (Supplementary Fig. 2, Additional File 1), in which values are plotted for each sex separately, can be used to verify that sex differences observed in plot type 1 are caused by local differences from the genome-wide mean in the heterogametic sex (as is expected for sex chromosomes), and not by deviations from the genome-wide mean in the homogametic sex.

The sex-specific signature observed in the mantled howler monkey across only parts of chromosome 3 may be the result of recombination suppression only extending to a part of the translocated chromosome, or if only parts of chromosome 3 were translocated to the X chromosome. The two outer ranges of the sex-linked region on chromosome 3 (134 and 178 Mb) may mark either the location of the fusion point between chromosome 3 and X, or a PAR boundary. There was no sex-specific signature on chromosome 15, which has been shown to be translocated to the Y chromosome in some *Alouatta* spp. (see above; [19, 29]). This may be either because (i) this chromosome is not translocated to the rest of the sex chromosome in this species, or (ii) that it is translocated but recombination suppression has not extended to this part of the sex chromosomes.

There are several reasons for running the pipeline without the use of a synteny-species: (i) when the study-species reference genome itself is of sufficiently high contiguity for the purposes of the study, (ii) when the purpose is to identify sex-linked scaffolds in the study-species reference genome, or (iii) when no suitable synteny-species reference genome is available. For highly contiguous study-species reference genomes, output plot types 1 (Fig. 3) and 2 (Supplementary Fig. 2, Additional File 1) may reveal large coherent sex-linked regions. However, in cases where the study-species reference genome is highly fragmented (as in the mantled howler monkey), plot type 3 (Fig. 4) is more likely to be of use. This plot type displays differences in sex-specific heterozygosity and genome coverage values per scaffold (as opposed to across specified genome window sizes), while also separating the scaffolds by length (Fig. 4a). The latter is valuable as short scaffolds may be responsible for much noise in the data.

To facilitate interpretation of plot type 3 (Fig. 4a), we produced a second plot (Fig. 4b) using the same data but where the scaffolds in the mantled howler monkey genome aligning to sex-linked regions identified when using human as synteny-species (Fig. 3) were coloured differently from the rest. Specifically, we coloured all scaffolds from the mantled howler monkey genome aligning with more than 90% of their length to the X chromosome (0–156 Mb) in blue, and those aligning to the sex-linked part of chromosome 3 (134–178 Mb) in red. All other scaffolds were coloured grey. Three clusters are clearly defined in the leftmost panels of Fig. 4b corresponding to different chromosomal categories (cf. Fig. 1): one (grey) cluster with no or little differentiation between sexes (autosomes), one (blue) with a slight deficiency of heterozygous sites and low genome coverage in the heterogametic sex (highly differentiated sex chromosomes), and one (red) with an excess of heterozygosity in the heterogametic sex and lower genome coverage in the heterogametic sex (low differentiation sex chromosomes). The effect of restricting the allowed number of mismatches between mapped reads and the reference genome for low differentiation sex-linked regions can be clearly seen in Fig. 4, as a cluster of scaffolds with an excess of heterozygosity and lower genome coverage in the heterogametic sex (red cluster in Fig. 4b) “moves” towards the autosomal cluster when allowing reads with more mismatches to be included (from left-side panel to right-side panel). Note that a second strategy to identify candidate sex-linked scaffolds in the study-species reference genome is to use the findZX-synteny option, and then extract the names of the scaffolds that were lifted over to the candidate sex-linked regions in the synteny-species reference genome (i.e., X: 0–156 and 3: 134–178 Mb; as done in Fig. 4b).

### *Validation through analysis of six* data sets

 To analyse the performance of this pipeline for sex chromosome systems of varying sizes and degrees of differentiation, we applied it to downloaded datasets for all the previously listed species (*n* = 6; Supplementary Table 1, Additional File 2). Plots supporting results that are specifically mentioned in the Main text are provided as Supplementary Figs. 5–18 (Additional File 1). In this section, we show excerpts of output plot type 4 (Fig. 5; see the full six-panel plots in Supplementary Figs. 5–9,11, Additional File 1). The underlying data for plot type 4 is the same as for plot type 1 (Fig. 3) and 2 (Supplementary Fig. 2, Additional File  1), but is in the format of scatter plots (with heterozygosity on the y-axis and genome coverage on the x-axis) instead of genome-wide plots (with chromosome position on the x-axis). The mean (± standard deviation) values for all genome windows per chromosome (here 1 Mb windows) are shown in Fig. 5. These values are also provided as output tables, which are included in the HTML report. We selected the previously identified sex chromosome(s) to be distinguished from other chromosomes or scaffolds with the “highlighting” option (see above). In the case of the ruff, the scaffold previously found to contain the 4.5 Mb large inversion polymorphism was highlighted [18]. Details on what chromosomes or scaffolds are expected to be sex-linked (or associated with male morphs) are in the Supplementary Methods (Additional File 1), and also summarized in Supplementary Table 3 (Additional File  4). 

In five of the six species, the expected chromosome(s)/scaffold(s) were clearly distinguished from the rest of the chromosomes/scaffolds (Fig. 5a-d, f). Among these were the platypus, where all five X chromosomes were identified through lower genome coverage in the heterogametic (XY) sex, which also had lower or similar levels of heterozygosity (chromosomes X_1_-X_5_; Fig. 5a; Supplementary Fig. 5, Additional File 1). This signature is expected for sex chromosomes of high Y (or W) degeneration (Fig. 1d). The two neo-sex chromosome systems were also clearly identified: the mantled howler monkey (chromosomes X, 3; Fig. 5b; Supplementary Fig. 6, Additional File 1) and the Eurasian skylark (chromosomes Z, 3, 4A, 5; Fig. 5c; Supplementary Fig. 7, Additional File 1). The sex chromosomes in these species consist of an ancestral region of high differentiation and Y/W degeneration as well as regions of more recent sex-linkage, which are characterized by less differentiation and Y/W degeneration. In both species, the ancestral sex chromosome regions were identified through lower genome coverage and equal to lower heterozygosity in the heterogametic sex (mantled howler monkey: chromosome X, Fig. 5b; Eurasian skylark: chromosome Z, Fig. 5c). The added regions were characterized by higher heterozygosity and similar-to-lower genome coverage in the heterogametic sex (mantled howler monkey: chromosome 3, Fig. 5b; Eurasian skylark: chromosomes 3, 4A and 5, Fig. 5c).

Two species, the guppy, and the central bearded dragon (Fig. 5d, e; Supplementary Figs. 8 and 9, Additional File 1), have sex chromosome systems that have been challenging to identify, due to either low differentiation (guppy) or due to difficulties in establishing homology to other species (central bearded dragon). Of these, we successfully identified the guppy (chromosome 12; Fig. 5d) sex chromosomes. The expected sex chromosome regions were not clear outliers in the central bearded dragon (chromosomes 17 and 23; Fig. 5e). However, chromosome 17 (Fig. 5e, in green), which is the chromosome syntenic to most identified sex-linked scaffolds in the central bearded dragon, did have the lowest male-to-female genome coverage of all chromosomes. One possible problem with this analysis is that only 2% of the 5 kb genome windows in the central bearded dragon was successfully anchored to the chicken reference genome (Supplementary Table 3, Additional File 4). However, we also ran an analysis of the central bearded dragon using the findZX option where the four main scaffolds identified as sex-linked were highlighted (Supplementary Fig. 10, Additional File 1) and the results were even less clear than when using the chicken as a synteny-species. We therefore hypothesize that the main problem with this species is the high heterozygosity observed among the central bearded dragon samples (~ 1%; Supplementary Table 4, Additional File 5), which may be too high for the method to be fully successful in this system. Lastly, the scaffold containing the inversion polymorphism controlling male morphs in the ruff (scaffold28/NW_015090842.1) was identified through having lower genome coverage and higher heterozygosity in the “faeder” phenotype individual (which is heterozygotic for the inversion) than in the “resident” phenotype individuals (which is homozygotic for the ancestral, non-inverted haplotype; Fig. 5f; Supplementary Fig. 11, Additional File 1).

### Robustness to evolutionary distances, sample sizes and genome coverage

To test for the effect of lowering the sample size for species whose sex chromosomes had been successfully identified based on more than one sample per sex, i.e. mantled howler monkey and guppy (Fig. 5b,d), we reran the pipeline using only 1 individual per sex. In both cases, the same regions were retrieved (Supplementary Figs. 12 and 13, Additional File 1). To test for sensitivity to sequencing depth, we subsampled the WGS data from 1 female and 1 male mantled howler monkey to 50% of the number of base pairs in the smallest of the mantled howler monkey samples. This did not affect the results either, as the same sex-linked regions were retrieved, even with as low as 1.43 × coverage with 0 mismatches allowed (Supplementary Fig. 14, Additional File 1; average genome coverage values for all discussed analyses are given in Supplementary Table 4, Additional File 5).

The results from the mantled howler monkey with human as synteny-species (Fig. 3) demonstrate that the pipeline is robust to large evolutionary distances between the studied species and the synteny-species, as howler monkeys (that belong to the New World monkeys) and humans separated over 35 million years ago [32]. As mentioned above, 78% of the 5 kb genome windows in the mantled howler monkey were anchored to the human reference genome (Supplementary Table  3, Additional File 4). We also ran the pipeline with an even more distant relative to the mantled howler monkey as a synteny-species; the meerkat (*Suricata suricatta*; Supplementary Table 2, Additional File 3 [9, 10]). Even though these species are separated by over 80 million years of independent evolution [8], we still managed to identify the sex-linked regions (homologous to meerkat chromosomes X and 5; Supplementary Fig. 15, Additional File 1). In this case, 39% of the 5 kb genome windows were successfully anchored (Supplementary Table 3, Additional File 4). Additionally, we ran the Eurasian skylark samples with the chicken (*Gallus gallus*) as a synteny-species reference genome and obtained the expected sex-linked regions (Supplementary Fig. 16 and 17, Additional File 1), with a 5 kb genome window anchoring percentage of 76% (compared with 89% to the zebra finch; Supplementary Table  3, Additional File 4). Lastly, we ran the ruff samples using the chicken as a synteny-species and found chromosome 11 to be an outlier, as predicted (Supplementary Methods and Supplementary Fig. 18, Additional File 1; 5 kb window anchoring percentage of 79%). Both the Eurasian skylark and the ruff separated from the chicken over 70 million years ago [24].

## Discussion

We present a novel Snakemake pipeline and demonstrate its effectiveness in uncovering sex-linked genomic regions using WGS data from a wide range of species and sex chromosome systems. The pipeline is easy to use, and the output plots can be easily customized in terms of what data is shown, thereby producing near-publication ready figures. The findZX-synteny option allows the user to quickly infer homology between sex chromosomes in different species, and we show that this method is effective even when the study-species and synteny-species are phylogenetically distant (Supplementary Figs. 15–18, Additional File 1). All heterozygosity and genome coverage values used to produce the figures, as well as outlier values outside the 95% CI genome-wide averages based on the specified window sizes (from plot type 1), are provided as separate output tables which facilitates further data exploration. A README file in the table directory describes each column, and names of output tables used for plotting are specified in each output plot.

Sex chromosomes can be identified using different kinds of genomic data [11, 20, 22, 26]. Here we utilize WGS data, which is more comprehensive compared to reduced representation sequencing techniques (such as RADseq) or RNA-seq, thereby increasing the likelihood of sex chromosome identification. It also provides opportunities for follow-up studies of e.g., gene content and repeat landscape of sex-linked regions. WGS sequencing is more expensive per sample than for example RADseq. However, the results from the multi-species analyses (Fig. 5) show that one sample per sex is sufficient to reveal sex-linked regions in most systems (Fig. 5; Supplementary Table 3, Additional File 4). We also show that the method is robust to low sequencing coverage (Supplementary Table 4 , Additional File 5). Note, however, that too low coverage will lead to an underestimation of heterozygous sites. This pipeline cannot be directly compared to most other published methods and software for detecting sex chromosomes, as it uses a different data type. Another pipeline that uses WGS data (SATC [21]) analyses genome coverage but not heterozygosity which we often found to be an essential component in separating sex chromosomes from autosomes, especially for low differentiation sex chromosome systems (see Fig. 5). The availability of complementary methods for identifying sex chromosomes is highly useful, as it allows the scientific community to take full advantage of the variety of sequence data that are being produced. 

Our pipeline successfully detected the previously identified sex-linked regions in four of the five studied species (Fig. 5a-d). One of these species was the guppy, in which the sex chromosomes have been notoriously difficult to characterise, even when analysing many samples per sex [34]. We were also able to identify the inversion polymorphism present in the ruff (Fig. 5f). However, while the expected sex chromosomes in the central bearded dragon (17 and 23) had more pronounced sex differences in genome coverage than most chromosomes, some chromosomes showed similar differences (Fig. 5d). Sequencing of additional individuals (we analysed data from only 1 male and 1 female individual), or the use of linkage mapping-based approaches may be necessary to successfully identify the sex chromosomes in highly heterozygous species such as this. Note also that when the fully sex-linked region is very small (as e.g., in the extreme example of the fugu [15]), our approach is likely too crude as it is based on genome window scans, and per-chromosome or -scaffold calculations. Our code is, however, open and modifiable for any special needs.

We urge users to inspect all the different output plots to avoid misinterpretations. For example, our method assumes that the type of heterogamety (XY or ZW) is known a priori. Should a heterogametic study-system reference genome be used instead of a homogametic one, one sex will show an excess of genomic windows without any (or with very low) coverage in the other sex (representing Y/W scaffolds). Plot type 5, which shows per-individual genome coverage and heterozygosity profiles (Supplementary Figs. 3 and 4, Additional File 1) may reveal if any of the included samples were not sexed correctly, and the pipeline can be rerun using correct heterogamety settings for these samples. It is also important to look not only at the female-to-male difference values (Fig. 3) when interpreting the results, but also at the values for the different sexes separately (Supplementary Fig. 2, Additional File 1). A low heterogametic-to-homogametic genome coverage signature may be caused by either lower coverage in the samples of the heterogametic sex, or by heightened coverage values in the samples of the homogametic sex. Furthermore, as exemplified by the analysis of the ruff, should studied females and males differ systematically by an autosomal inversion, this would produce similar genome signatures as a sex chromosome. Therefore, independent validation of sex-linked regions found using this pipeline is recommended, for example through PCR and Sanger sequencing of targeted loci in additional samples, especially when analysing only one sample of each sex (cf. [28]).

## Conclusions

Uncovering novel changes in sex chromosome systems between species is important to advance our understanding of sex chromosome evolution. Furthermore, failing to consider unexpected sex chromosome variation may lead to misinterpretations of genomic patterns in studies not related to sex chromosome research. As WGS data becomes increasingly available (and published on open databases such as NCBI’s short read archive), we hope that our pipeline will be of use to many researchers.

### Availability and requirements

Project name: findZX.

Project home page: https://github.com/hsigeman/findZX

Repository: https://github.com/hsigeman/findZX

Operating system(s): Linux; MacOS.

Programming language: R, bash, Python/Snakemake.

Other requirements: Conda.

License: GNU GPL version 3.

Any restrictions to use by non-academics: None.

## Supplementary Information

**Additional file 1.** Supplementary Information. Contain Supplementary Methods and Supplementary Figures 1-22.

**Additional file 2. Supplementary Tabl e 1.  **

**Additional file 3. Supplementary Table 2.  **

** Additional file 4. Supplementary Table 3. **

**Additional file 5. Supplementary Table 4.**

**Additional file 6. Supplementary Table 5.**

## Data Availability

The findZX pipeline is available on GitHub: https://github.com/hsigeman/findZX, along with instructions on how to install and configure findZX for analyses on new datasets. A short tutorial is also provided in the Supplementary Information (Additional File 1). NCBI accession numbers for all paired-end WGS data used in this study are listed in Supplementary Tables 1 (Additional File 2) and 4 (Additional File 5). GenBank assembly accession numbers or Dryad archive links for the reference genomes used in this study are listed in Supplementary Table 2 (Additional File 3). Paths to configuration files for all findZX analyses are also given in Supplementary Table 3 (Additional File 4).

## Abbreviations

WGS: Whole-genome sequencing
Kb: Kilo-base pair
Mb: Mega-base pair
Gb: Giga-base pair
PAR: Pseudoautosomal region
SD: Standard deviation
CI: Confidence interval
PCR: Polymerase chain reaction
NCBI: National Center for Biotechnology Information
