# Supplementary material for: Findzx: an automated pipeline for detecting and visualising sex chromosomes using whole-genome sequencing data
Source: BMC Genomics. 2022 Apr 27;23:328. doi: 10.1186/s12864-022-08432-9 (PMC9044604; doi:10.1186/s12864-022-08432-9)
Supplement: Supplementary file 1 — Additional file 1. Supplementary Information. Contain Supplementary Methods and Supplementary Figures 1-22. [file 12864_2022_8432_MOESM1_ESM.pdf]

Supplementary Information

FindZX: an automated pipeline for detecting and visualising sex chromosomes using whole-genome sequencing data

Authors: Hanna Sigeman, Bella Sinclair, Bengt Hansson

1 Supplementary Methods

1.1 Details of software and settings used by findZX

Information on how to install findZX is in the Supplementary Methods below and on the GitHub page (https://github.com/hsigeman/findZX). All needed software and dependencies, which are fully compatible with both Linux (64-bit) and macOS systems, can be easily installed using the Conda package manager (Anaconda Software Distribution, 2016). The minimal data requirements for running findZX is paired-end WGS data from one individual of each sex, as well as a reference genome of the homogametic sex from the same species (Figure 2a). If no reference genome is available for the study-species, it can be constructed based on the paired-end data from the homogametic sample (see instructions on the GitHub page). To run findZX-synteny, a reference genome of the species that should be used for the genome coordinate liftover analysis is also required (Figure 2a). The analysis will be run according to settings provided in a configuration file, which also contains paths to input data (see the GitHub page and below for usage examples). The following sections describe the different computational steps performed by findZX.

1.1.1 Trimming and subsampling (optional)

The first step performed by findZX, if opted for in the configuration file, is trimming and quality filtering of the fastq reads using Trimmomatic v0.39 [1]. An HTML quality control report will be created for both the untrimmed and trimmed reads, using fastqc v0.11.9 [2] and multiqc v1.10.1 [3]. Very large fastq files can take a long time to process (especially the alignment of reads to the reference genome). FindZX therefore includes an option to subsample reads to a specific number of basepairs using reformat.sh (BBTools suite v38.92, Bushnell). The pipeline is written in a modular way so that it can be stopped after the trimming and HTML report are completed, to allow for visual inspection of the results before running the rest of the steps. FindZX can then be restarted if the trimming was successful.

1.1.2 Alignment and variant calling

The reads from each sample are then aligned to the reference genome of the study species using bwa mem v0.7.17 [4], sorted using samtools sort v1.7 [5] and deduplicated with picardtools v2.18.0 (http://broadinstitute.github.io/picard). Variants are called for all samples using platypus v0.8.1.1 [6] and compressed using bgzip and tabix v1.9 [7].

1.1.3 Heterozygosity and genome coverage calculations

Variants are filtered with vcftools v0.1.15 [8] using options –gzip –min-alleles 2 –max-alleles 2 –minQ 20 –MinDP 3 and –remove-filtered-geno-all. The number of heterozygous sites is summed across 5 kb genome windows for each individual. For each genome window, the mean proportion of heterozygous sites (number of heterozygous sites per 100 base pairs) is calculated for each sex. The heterozygosity of each sample is reported in a separate file (number of heterozygous sites/genome length).

Genome coverage is calculated using bedtools multicov v2.27.1 [9] (only considering properly aligned reads with quality score >20) across the same 5 kb genome windows. This is done on the original deduplicated BAM files (see above) which will be referred to as ”unfiltered”, and on two other BAM files that are filtered to only contain reads with different thresholds of mismatches to the reference genome (thresholds specified in the configuration file). The thresholds used for all analyses in this paper are i) ”unfiltered”, ii) intermediate filtering ($\leq$2 mismatches allowed), and iii) strict filtering (0 mismatches allowed). The genome coverage values for each sample in each of these three BAM files are first filtered for outliers (values exceeding the genome-wide mean three times the standard deviation are masked), and then normalized between samples. Alignment statistics for each BAM file are generated using samtools stats v1.7.

1.1.4 Chromosome anchoring to a synteny-species (only when using findZX-synteny)

If findZX-synteny is used, a lastdb [10] database will be constructed from the synteny-species reference genome. Whole-genome alignment between the study-species and synteny-species reference genomes (using the lastdb database) will then be performed with lastal, and converted to psl format (using maf-convert); both programs from the software last v1238 [10]. A custom script then locates the longest match for each 5 kb window in the study-species reference genome to the synteny-species reference genome, with 500 matching base pairs as a minimum. Matches where more than two windows from the study-species matched to the same chromosome position in the synteny-species reference genome are filtered out. The proportion of 5 kb windows that were matched to the synteny-species reference genome also reported.

1.1.5 Statistics, plotting and HTML report

Mean heterozygosity (from the "unfiltered" BAM file) and genome coverage values (from BAM files with all three mismatch filtering settings; see above) are calculated per sex and chromosome/scaffold, and per sex across different genome window sizes. The window sizes are specified in the configuration file (for the analyses in this paper we chose 50 kb, 100 kb and 1 Mb for all species). From these values, differences between sexes are calculated and written as output tables. Genome-wide mean ±95% confidence intervals (CI) are calculated for the heterozygosity and genome coverage data, and another set of tables is written with windows having values on either side of these thresholds (mean ±95% CI).

The sex differences in heterozygosity and genome coverage are plotted in different formats (see Results section and Supplementary Figures for examples) per chromosome/scaffold, and per window size. If a list of chromosomes/scaffolds is provided in the configuration file, these are the only ones that will be plotted. If no such file is provided all chromosomes/scaffolds will be plotted, except for the genome-wide plots (plot type 1 and 2) which require a pre-set maximum number of scaffolds (default: n = 50). FindZX also provides output plots that can be used for verifying the sex of the studied samples by plotting heterozygosity and genome coverage profiles for each sample separately (plot type 5). Note, however, that these results are clearer for species with large and heteromorphic sex chromosomes.

All output plots are in multi-page PDF format, where the last page contain information on what output table was used to create each plot. All output plots can be rendered into an interactive HTML report file, which will also contain run time information and the multiqc report files (see Supplementary Methods/GitHub page for details).

1.1.6 Consensus genome assembly

Results from a previous study showed that sex chromosome regions of low differentiation (Figure 1a) can be hard to identify in highly heterozygous species, when only one sample of each sex is used and when the reference genome is constructed from one of these samples [11]. This is because identification of such regions often requires strict filtering of mismatches to the reference genome (Figure 4), and in highly heterozygous species this may lead to a drastic reduction of aligned reads both from the autosomes and the sex chromosomes in the heterogametic sample which will obscure the signal from the sex chromosomes (see [11] for more details). To circumvent this problem, findZX has an option to create a consensus reference genome using bcftools consensus, in which all biallelic variants with non-reference allele counts ≥2 will be incorporated. The consensus reference genome is created from a filtered version of the output VCF file (Figure 2a; Step 8). This new consensus genome assembly can then be used as input to the pipeline instead of the original reference genome. See Supplementary Methods and/or the GitHub page for instructions on how to run this.

1.1.7 Per-sample average genome coverage calculations

We performed one analysis in this paper that was not part of the pipeline: calculations of an average genome coverage value per sample (Supplementary Table 4, Additional File 5). These calculations were done using a bash script (available on GitHub at workflow/scripts/calc_cov.sh) which takes as input the output file from samtools stats (see above) and the indexed study-species reference genome (produced with samtools faidx). The script divides the total number of aligned base pairs in each sample by the total length of the respective study-species reference genome.

1.2 How to install and run findZX

1.2.1 Download and installation

FindZX works on Linux and macOS systems, and contains a configuration file which can be used to run the pipeline on a SLURM system. There is only one prerequisite (except for findZX itself): that conda (`https://docs.conda.io/en/latest/`) is installed on the system. Conda will download and install all other dependencies into separate conda environments the first time findZX/findZX-synteny is run.

Obtain a copy of findZX from the GitHub page:

```
git clone https://github.com/hsigeman/findZX.git
cd findZX # Go to directory
```

Create a minimal conda environment and install software automatically through findZX:

```
conda create -n findZX -c conda-forge -c bioconda \
python=3.9.4 snakemake-wrapper-utils=0.2.0 snakemake=6.4.0 \
mamba=0.15.3
```

Then activate the environment:

```
conda activate findZX
```

To verify the installation, run findZX on a test dataset:

```
snakemake -s workflow/findZX --configfile .test/config.yml \
--cores 1 -R all --use-conda -k
```

For more information, please visit the GitHub page (`https://github.com/hsigeman/findZX.git`).

1.2.2 Basic usage

FindZX can be run using the following command:

```
snakemake -s workflow/findZX \
--configfile <Config file> \
--cores <Select number of cores> -R all \
--use-conda -k
```

FindZX-synteny can be run using the following command:

```
snakemake -s workflow/findZX-synteny \
--configfile <Config file> \
--cores <Select number of cores> -R all \
--use-conda -k
```

Note that computational steps shared by findZX and findZX-synteny (Figure 2a in the Main text; Steps 1-10) do not need to be rerun when changing between using findZX and findZX-synteny. If an analysis is first performed using findZX, and later using findZX-synteny (adding a synteny-species), only Steps 13-17 are run. Similarly, if an analysis is first performed using findZX-synteny

and then findZX, only Steps 11-12 are run. This is because snakemake automatically keeps track of which files need to be created, and which ones are already present.

To render an interactivate HTML report with all output plots and run times, run the following command once findZX/findZX-synteny is finished:

```
snakemake -s workflow/findZX{-synteny} \
--configfile <Config file> \
--report <Report_name.html>
```

The pipeline can also be run on a SLURM server cluster, by specifying a SLURM configuration file (cluster.yml):

```
snakemake -s workflow/findZX \
--configfile <Config file> \
--cores <Select number of cores> -R all \
--cluster-config cluster.yml \
--cluster " sbatch -A {cluster.account} -t {cluster.time} \
-n {cluster.n} " \
--use-conda -k
```

For more information on how to use findZX/findZX-synteny, see instructions on the GitHub page (`https://github.com/hsigeman/findZX`)

1.2.3 Constructing a consensus genome

Use this command to run the ”consensus genome” option (see Main text):

```
snakemake -s workflow/findZX \
--configfile <Config file> \
--cores <Select number of cores> -R modify_genome \
--use-conda -k
```

See below (*Alauda arvensis* analysis under subsection 1.2.1) for example usage.

1.2.4 Reproducing the results in this paper

The commands used to run all analyses are described for each species under subsection 1.2, and summarized in Supplementary Table 3 (Additional File 4).

1.3 Information on the sex chromosome systems of the studied species, and details on findZX analyses:

Here, we give brief information about the current knowledge of the sex chromosome system in each of the studied species (or inversion polymorphism in the case of the ruff), and how each species was analysed. In species where a synteny-species reference genome was used to anchor the scaffolds/chromosomes from the study-species reference genome, we list which chromosomes are expected to show signs of sex-linkage in each synteny-species. These expectations are based either on results from previous studies or based on known syntenies between species (as determined by https://www.genomicus.biologie.ens.fr/).

Config files listed here are on the findZX GitHub page.

1.3.1 *Alauda arvensis* (Eurasian skylark)

Sex chromosome system:
Neo-sex chromosome system with shared synteny to zebra finch (*Taeniopygia guttata*) chromosomes Z, 3, 4A and 5 [11, 12]

Details on findZX analyses:
The Eurasian skylark WGS samples (n = 2; Supplementary Table 1; Additional File 2) were analysed using a (highly fragmented) study-species reference genome constructed from the male WGS sample (Supplementary Table 1, Additional File 2; Supplementary Table 3, Supplementary File 4; see Sigeman et al. [11]).

We ran the findZX pipeline three times for this species. First, we used the "consensus reference genome" option, to ensure equal mapping success to the reference genome between sexes (with findZX):

```
snakemake -s workflow/findZX --configfile \
config/9_species_config/Alauda_arvensis_config.yml \
--cores 25 -R modify_genome --use-conda -k
```

Then, we ran the pipeline using this consensus reference genome as input to the pipeline, using the zebra finch (*Taeniopygia guttata*) as a synteny-species (with findZX-synteny):

```
snakemake -s workflow/findZX-synteny --configfile \
config/9_species_config/Alauda_arvensis_consensus_config_ZF.yml \
--cores 25 -R all --use-conda -k
```

And lastly using the chicken (*Gallus gallus*) as a synteny-species (with findZX-synteny):

```
snakemake -s workflow/findZX-synteny --configfile \
config/9_species_config/Alauda_arvensis_consensus_config_GG.yml \
--cores 25 -R all --use-conda -k
```

Expected sex-linked regions:
Synteny-species 1 (zebra finch): chromosomes Z, 3, 4A and 5 [11, 12].
Synteny-species 2: (chicken): chromosomes Z, 3, 4 and 5 (Based on synteny between zebra finch and chicken genomes: https://www.genomicus.biologie.ens.fr/.

1.3.2 *Alouatta palliata* (mantled howler monkey)

Sex chromosome system:
Multiple sex chromosome system (X_1X_2Y) [13]. The X_2 chromosome in *A. caraya* (a relative of *A. palliata*) share synteny with human chromosomes 3 and 15 [14].

Details on findZX analyses:
The mantled howler monkey WGS samples (n = 4; Supplementary Table 1, Additional File 2) were

analysed using a fragmented study-species reference genome (Supplementary Table 3, Additional File 4). We first ran the pipeline using all samples (n = 4) and with the findZX option:

```
snakemake -s workflow/findZX --configfile \
config/9_species_config/Alouatta_palliata_config.yml \
--cores 25 -R all --use-conda -k
```

Then, we used the human (*Homo sapiens*) reference genome (Supplementary Table 3, Additional File 4) as a synteny-species (findZX-synteny):

```
snakemake -s workflow/findZX-synteny --configfile \
config/9_species_config/Alouatta_palliata_config.yml \
--cores 25 -R all --use-conda -k
```

We then ran the pipeline (again with human as a synteny-species) using only one sample of each sex (n = 2):

```
snakemake -s workflow/findZX-synteny --configfile \
config/9_species_config/Alouatta_palliata_config_1M_1F.yml \
--cores 25 -R all --use-conda -k
```

Then with downsampling (to 50 % of the basepairs in the sample with the smallest number of reads) of the same two samples (n = 2):

```
snakemake -s workflow/findZX-synteny --configfile \
config/9_species_config/Alouatta_palliata_config_subsampling_1M_1F.yml \
--cores 25 -R all --use-conda -k
```

Then, lastly using the meerkat (*Suricata suricatta*) (Supplementary Table 3, Additional File 4) as synteny-species reference genome (findZX-synteny):

```
snakemake -s workflow/findZX-synteny --configfile \
config/9_species_config/Alouatta_palliata_config_SS.yml \
--cores 25 -R all --use-conda -k
```

Expected sex-linked regions:
Study-species: Unknown
Synteny-species 1 (human): chromosomes X, 3, 15 [14].
Synteny-species 2: (meerkat): chromosomes X, 5, 9 (Based on synteny between human and meerkat genomes: https://www.genomicus.biologie.ens.fr/.

1.3.3 *Calidris pugnax* (ruff)

Inversion polymorphism:
Scaffold28 contains the 4.5 Mb large inversion polymorphism controlling male phenotypes in the ruff [15]. Scaffold28 shares synteny with chicken chromosome 11 [15].

Details on findZX analyses:
The ruff samples (n = 3; Supplementary Table 1, Additional File 2) were analysed using the scaffold-level reference genome of the ruff (Supplementary Table 3, Additional File 4) as a study-species reference genome. The pipeline was run twice; first without a synteny-species reference genome (findZX):

```
snakemake -s workflow/findZX --configfile \
config/9_species_config/Calidris_pugnax_config.yml \
--cores 25 -R all --use-conda -k
```

And then using the chicken as a synteny-species reference genome (findZX-synteny):

```
snakemake -s workflow/findZX-synteny --configfile \
config/9_species_config/Calidris_pugnax_config.yml \
--cores 25 -R all --use-conda -k
```

Expected inversion polymorphic region:
Study-species: scaffold28/NW_015090842.1 [15].
Synteny-species (chicken): chromosome 11 [15].

1.3.4 *Ornithorhynchus anatinus* (platypus)

Sex chromosome system:
XY-system consisting of five X chromosomes and five Y chromosomes: $X_{1\text{-}5}Y_{1\text{-}5}$ [16].

Details on findZX analyses:
The platypus WGS samples (n = 2; Supplementary Table 1, Additional File 2) were analysed (findZX) using a chromosome-level study-species reference genome (Supplementary Table 3, Additional File 4):

```
snakemake -s workflow/findZX --configfile \
config/9_species_config/Ornithorhynchus_anatinus_config.yml \
--cores 25 -R all --use-conda -k
```

Expected sex-linked regions:
Study-species: Chromosome X_1-X_5 [16].

1.3.5 *Poecilia reticulata* (guppy):

Sex chromosome system:
Extremely undifferentiated XY-system [17, 18], LG12/chr12.

Details on findZX analyses:
All guppy WGS samples (n = 23; Supplementary Table 1, Additional File 2) were analysed (findZX) using a chromosome-level study-species reference genome (Supplementary Table 3, Additional File 4):

```
snakemake -s workflow/findZX --configfile \
config/9_species_config/Poecilia_reticulata_config.yml \
--cores 25 -R all --use-conda -k
```

And then using two (n= 2) of the original WGS samples:

```
snakemake -s workflow/findZX --configfile \
config/9_species_config/Poecilia_reticulata_config_1M_1F.yml \
--cores 25 -R all --use-conda -k
```

Expected sex-linked regions:
Study-species: Chromosome 12/LG12 [17, 18].

1.3.6 *Pogona vitticeps* (central bearded dragon)

Sex chromosome system:
Micro-ZW system. Mainly shares synteny with chicken microchromosomes 17, but also with 23 to a very small degree [19].

Details on findZX analyses:
The central bearded dragon samples (n = 6; originating from 2 individuals; Supplementary Table 1,

Additional File 2) were analysed using the scaffold-level reference genome of the central bearded dragon (Supplementary Table 3, Additional File 4) as a study-species reference genome. The pipeline was run using the chicken as a synteny-species reference genome (findZX-synteny):

```
snakemake -s workflow/findZX-synteny --configfile \
config/9_species_config/Pogona_vitticeps_config.yml \
--cores 25 -R all --use-conda -k
```

And then witout a synteny-species reference genome (findZX):

```
snakemake -s workflow/findZX --configfile \
config/9_species_config/Pogona_vitticeps_config.yml \
--cores 25 -R all --use-conda -k
```

Expected sex-linked regions:
Study-species: CEMB01002638.1, CEMB01011940.1, CEMB01037558.1, CEMB01017046.1 [19].
Synteny-species (chicken): Chromosome 17 and (partly) 23 [19].

2 Supplementary Figures

2.1 Example output - plot types 1-5

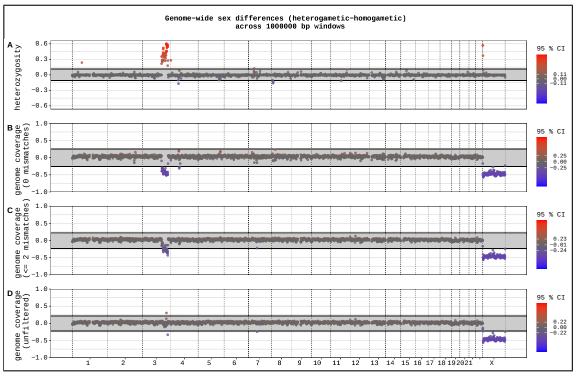

(**A**) Plot type 1

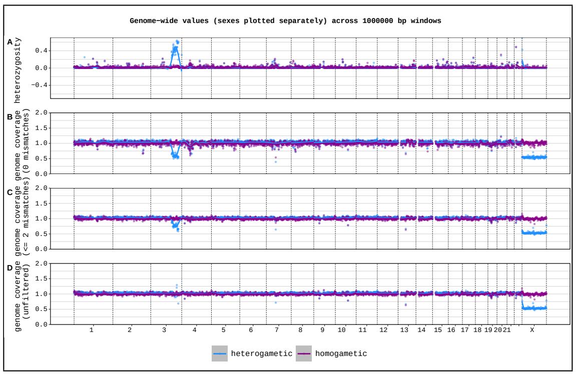

(**B**) Plot type 2

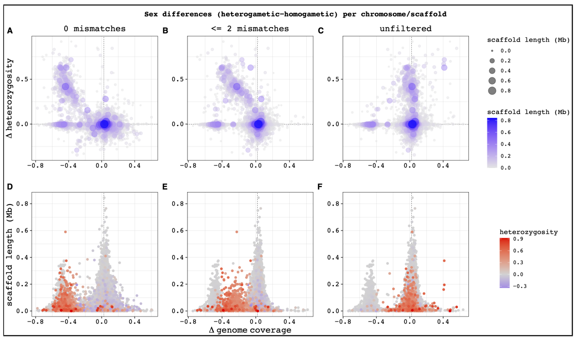

(**C**) Plot type 3

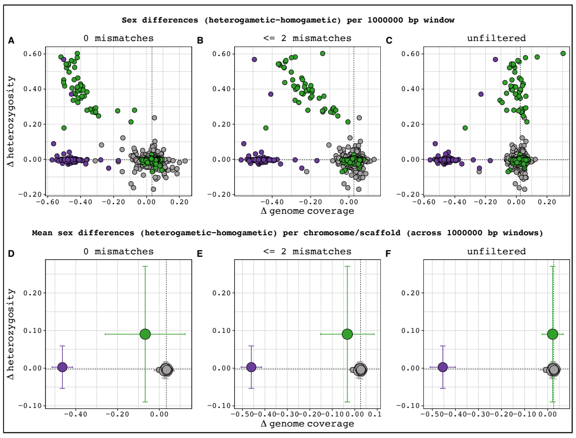

(**D**) Plot type 4

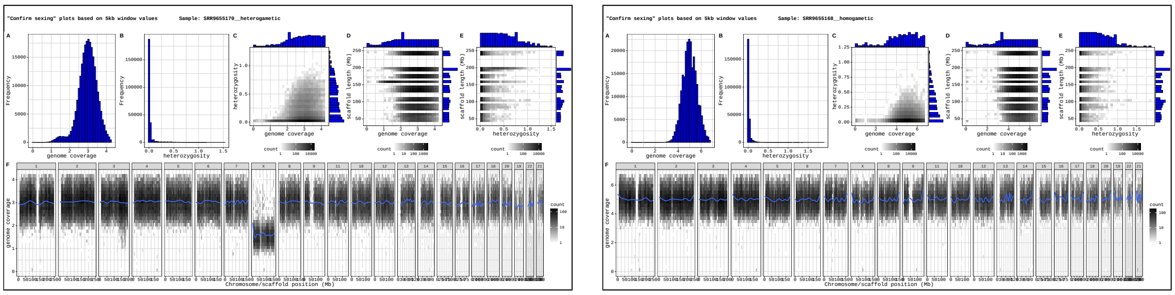

(**E**) Plot type 5 (two different individuals)

Supplementary Figure 1: FindZX will produce five types of output plots (here shown as miniatures **A-E)**, all based on heterozygosity and genome coverage values. **(A):** Genome-wide sex differences across specified genome windows (here 1 Mb; see Figure 3 in main text). **(B):** Genome-wide sex-specific values across specified genome windows (here 1 Mb; see Supplementary Figure 2). **(C):** Per chromosome/scaffold values (bottom row also include chromosome/scaffold length; see Figure 4 in main text). **(D):** Genome window values (here 1 Mb; upper row), and per-chromosome/scaffold values based on the same windows (bottom row; see Figure 5 in main text). **(E):** "Confirm sexing" plots. Differences in genome coverage and heterozygosity profiles may reveal if some samples were given the wrong sex (see Supplementary Figure 3 and 4).

2.2 Example output - plot type 2

Mantled howler monkey (*A. palliata*)

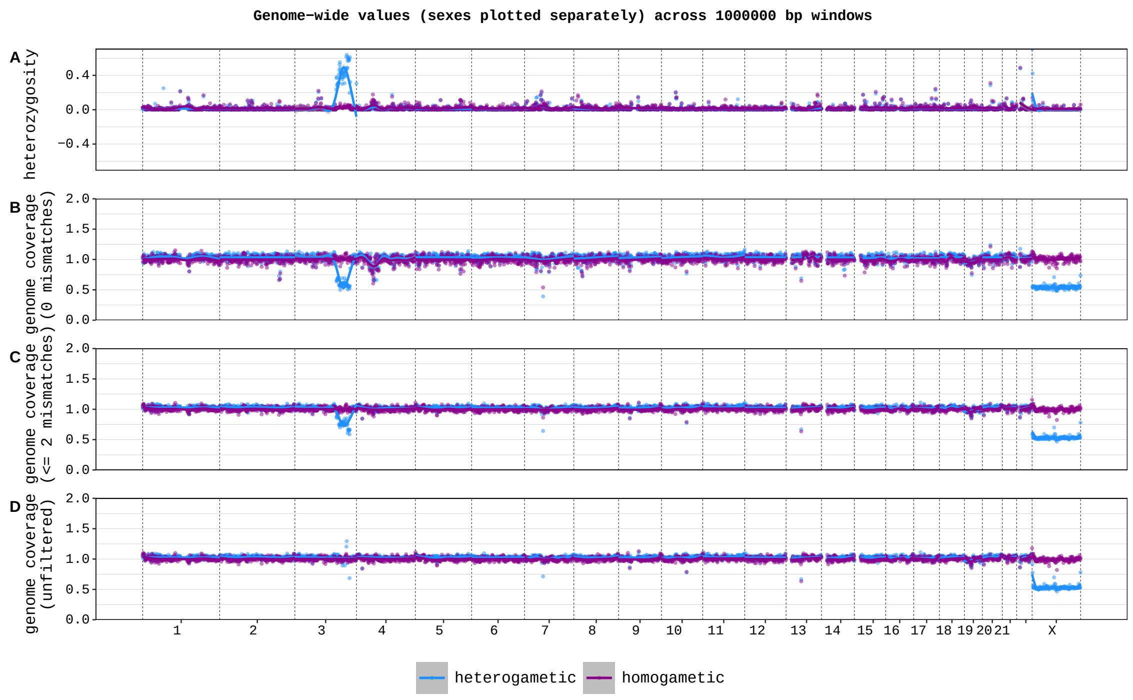

Supplementary Figure 2: Per-sex genome coverage and heterozygosity values (1 Mb windows) for mantled howler monkey, plotted along chromosome positions in the human genome. The four rows show: (**A**) heterozygosity, and genome coverage with (**B**) strict filtering (0 mismatches allowed), (**C**) intermediate filtering ($\leq$2 mismatches) and (**D**) no filtering of mapped reads (unfiltered). The values for each sex are plotted separately, with a smoothing line for each sex (heterogametic in blue, homogametic in purple). The data confirms that chromosome X and a part of chromosome 3 are sex-linked in this species [14]. It also shows that the sex differences observed on chromosomes X and 3 (see Figure 3 in Main text) are due to deviations from the genome-wide mean in the heterogametic sex, as expected for sex chromosomes.

2.3 Example output - plot type 5

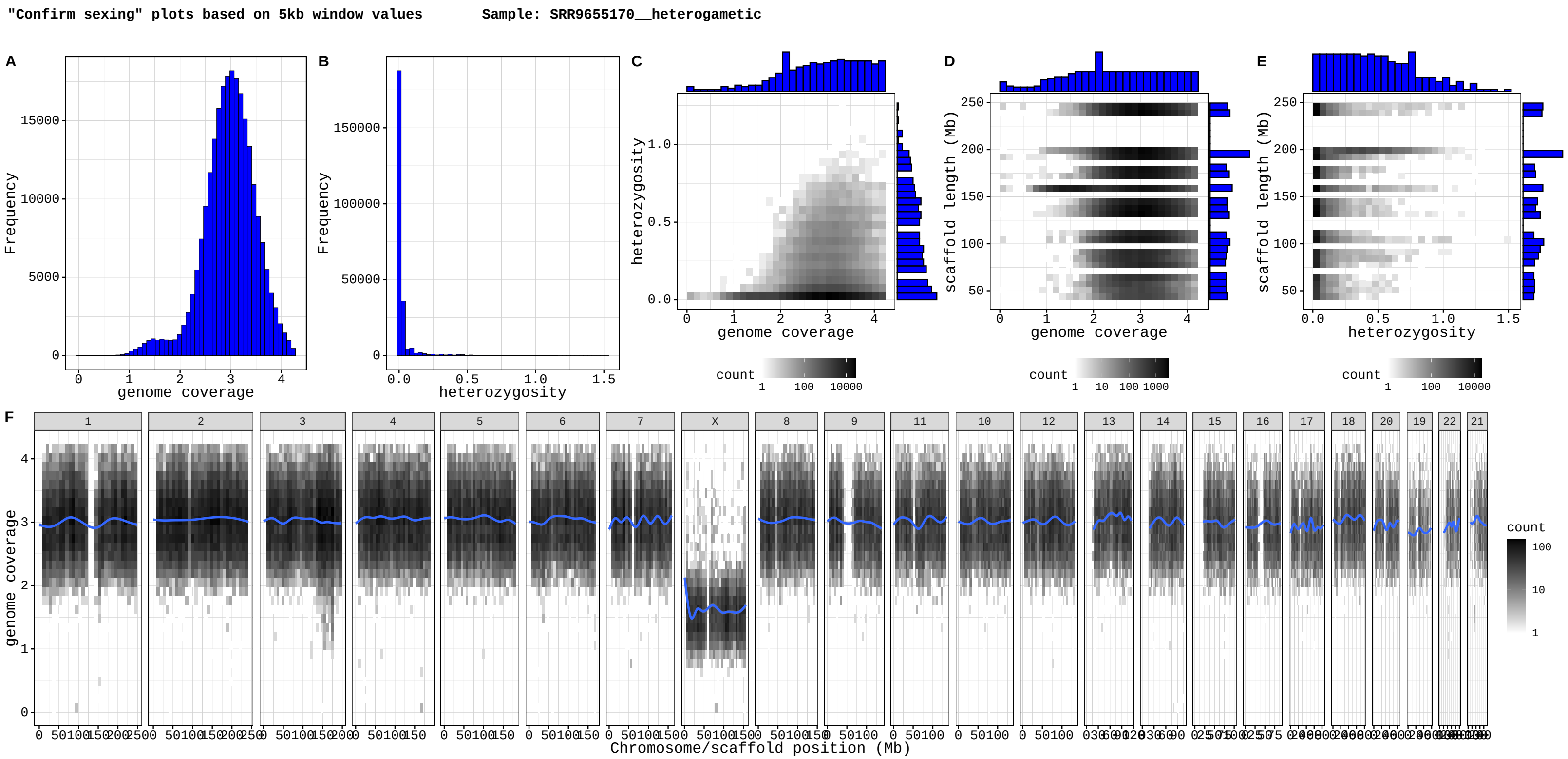

Supplementary Figure 3: Example of a "Confirm-sexing" plot (see Figure 2 in main text), from a male (XY) mantled howler monkey sample (*A. palliata*). Histogram of (**A**) genome coverage and (**B**) heterozygosity per 5 kb window. (**C**) Heatmap of genome coverage and heterozygosity values per 5 kb window. Heatmaps of (**D**) genome coverage and (**E**) heterozygosity (on the x-axis) and scaffold length (on the y-axis). (**F**) Heatmap of genome coverage values along the 50 largest chromosomes/scaffolds (or a selected list of chromosomes/scaffolds). The genome coverage on the X chromosome (**F**) is strongly reduced, due to the high degeneration of the Y chromosome. The genome coverage histogram (**A**) also show a slight bimodal distribution, suggesting this is a heterogametic individual.

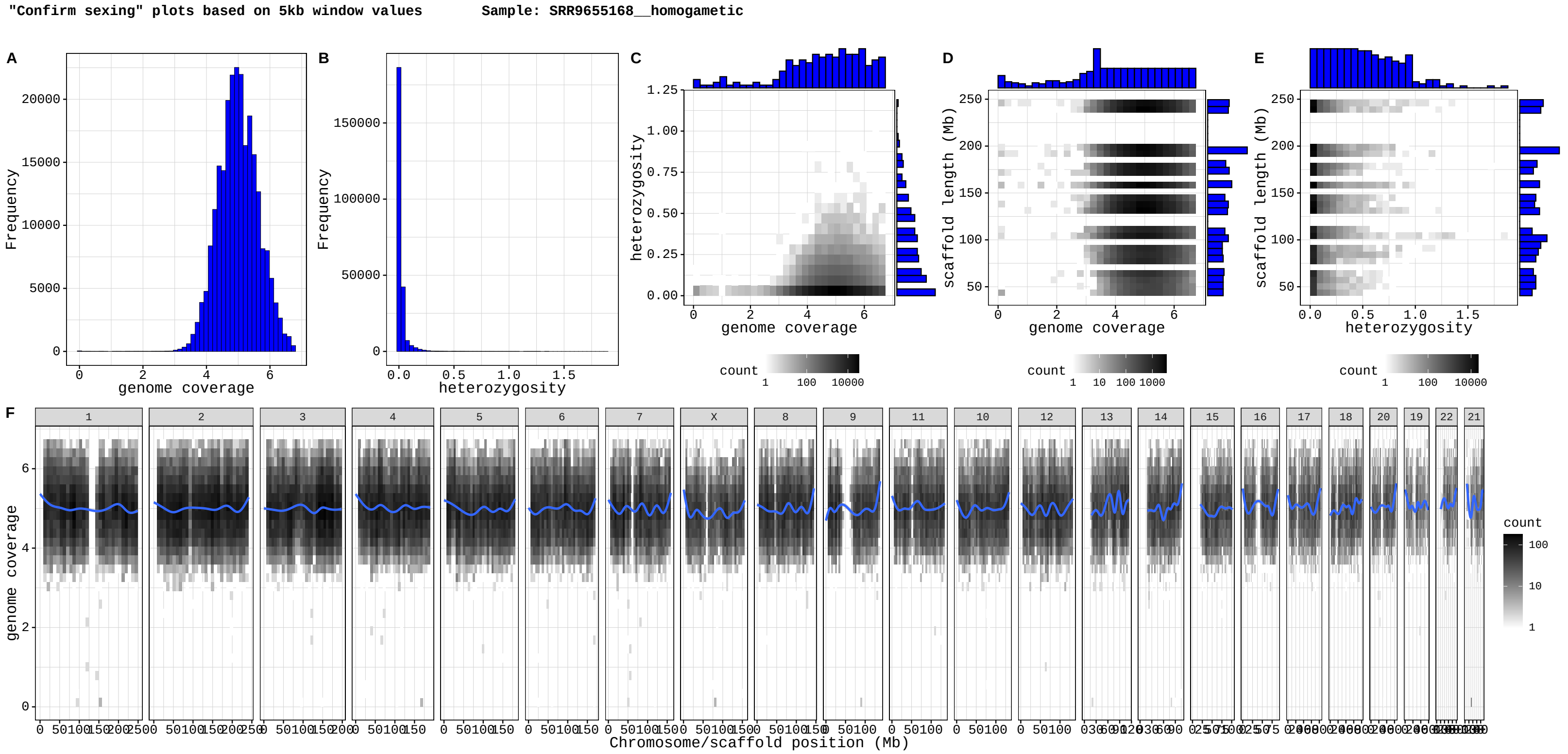

Supplementary Figure 4: Example of a "Confirm-sexing" plot (see Figure 2 in main text), from a female (XX) mantled howler monkey sample (*A. palliata*). Histogram of (**A**) genome coverage and (**B**) heterozygosity per 5 kb window. (**C**) Heatmap of genome coverage and heterozygosity values per 5 kb window. Heatmaps of (**D**) genome coverage and (**E**) heterozygosity (on the x-axis) and scaffold length (on the y-axis). (**F**) Heatmap of genome coverage values along the 50 largest chromosomes/scaffolds (or a selected list of chromosomes/scaffolds). The genome coverage on the X chromosome (**F**) is similar to the autosomes and the genome coverage histogram (**A**) show a normal distribution, suggesting this is a homogametic individual.

2.4 Output plots type 4 from all six studied species

Platypus (*O. anatinus*)

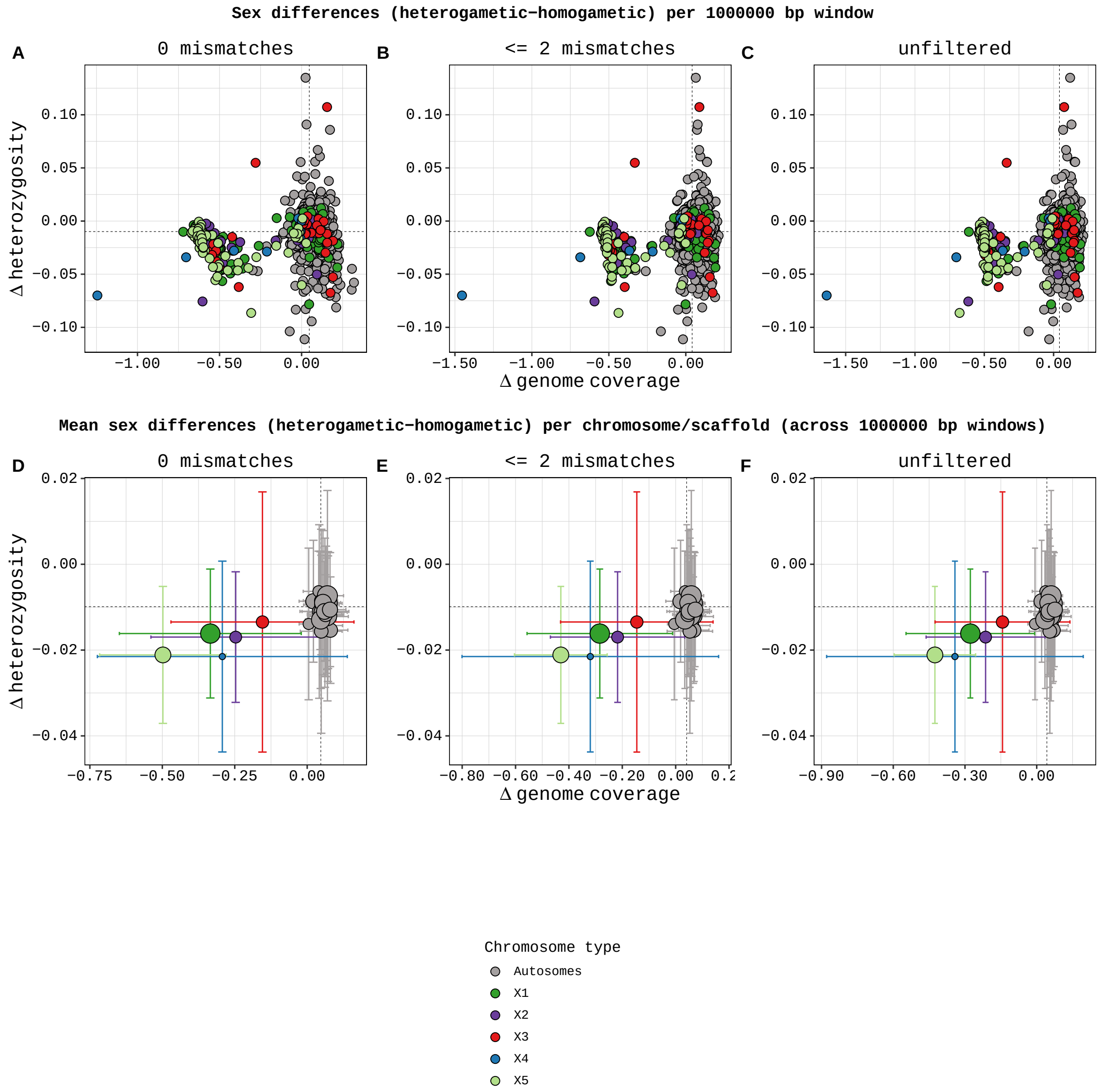

Supplementary Figure 5: (**A-C**) Sex differences in genome coverage and heterozygosity for all 1 Mb genome windows. (**D-F**) Mean (± SD) sex differences in genome coverage and heterozygosity per chromosome/scaffold, calculated from the 1 Mb genome windows. Dashed lines mark the genome-wide median across all 1 Mb windows. Data from 1 male and 1 female platypus (*O. anatinus*), analysed using findZX (without the use of a synteny-species reference genome. The previously identified sex chromosomes in this species (X_1, X_2, X_3, X_4 and X_5 [16]) are clear outliers.

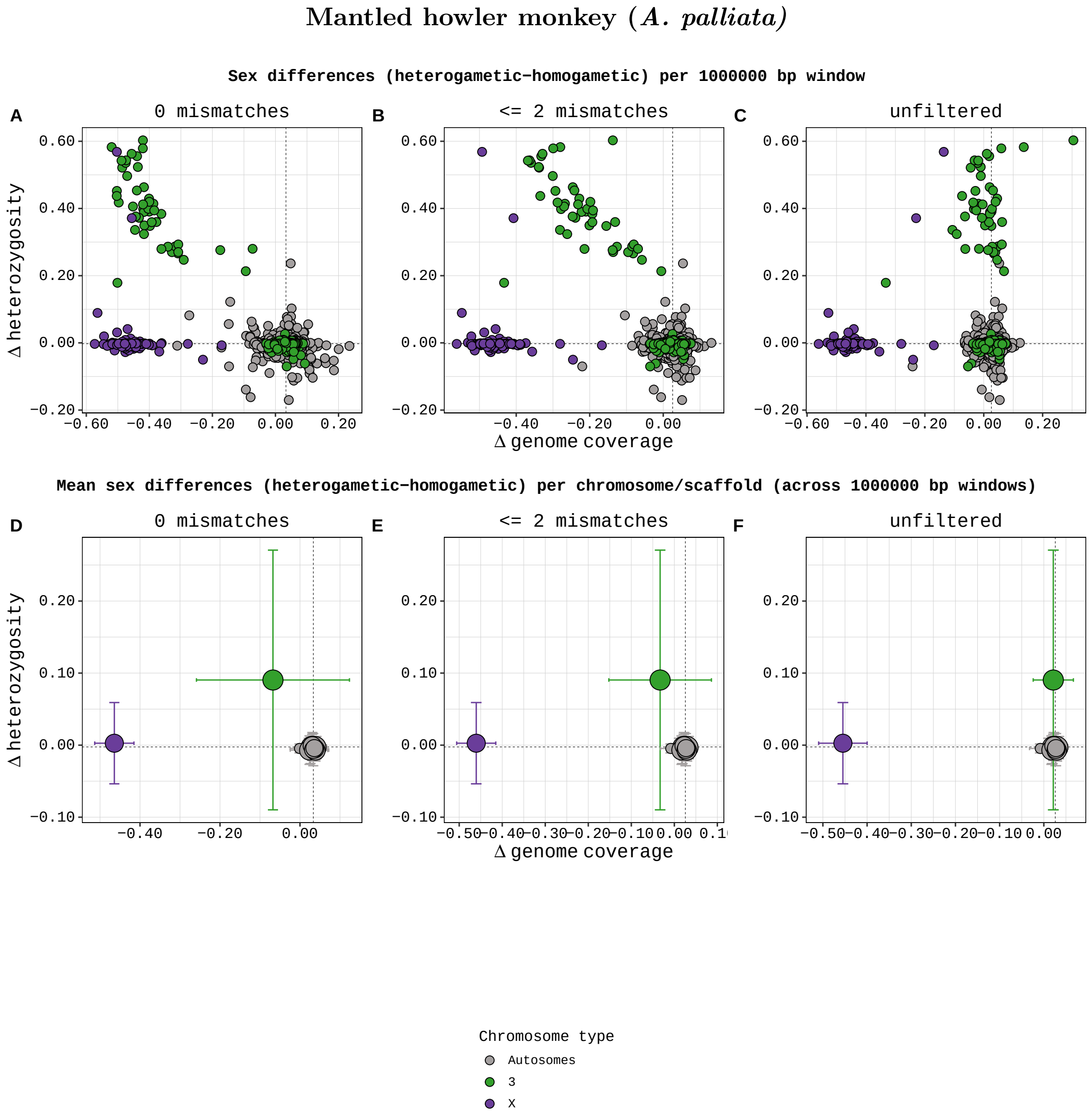

Supplementary Figure 6: (**A-C**) Sex differences in genome coverage and heterozygosity for all 1 Mb genome window. (**D-F**) Mean (± SD) sex differences in genome coverage and heterozygosity per chromosome/scaffold, calculated from the 1 Mb genome windows. Dashed lines mark the genome-wide median across all 1 Mb windows. Data from 2 male and 2 female mantled howler monkeys (*A. palliata*), analysed using findZX-synteny and human (*H. sapiens*) as a synteny-species reference genome. The previously identified sex chromosomes in this species (X and 3 [14]) are clear outliers.

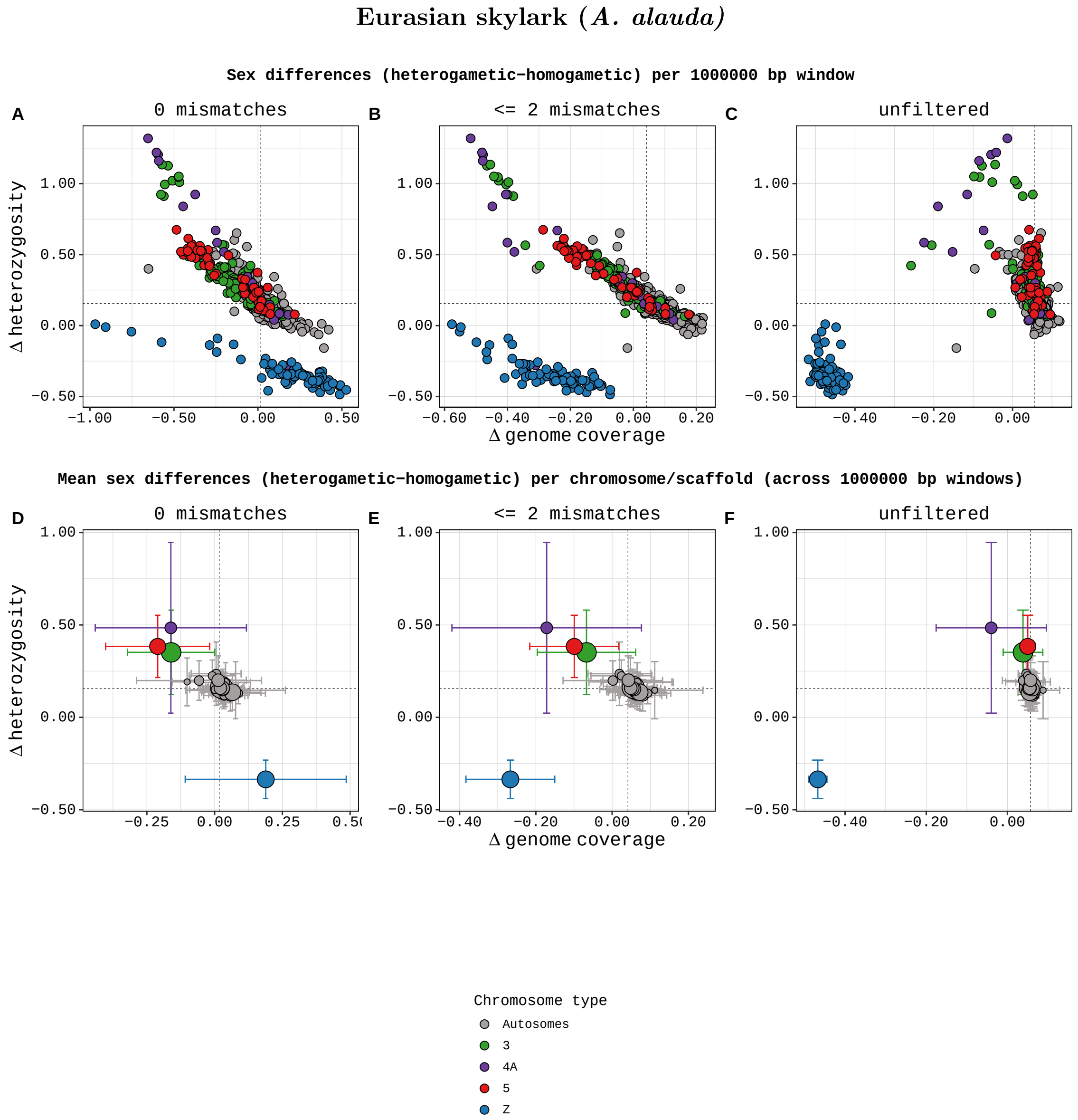

Supplementary Figure 7: (**A-C**) Sex differences in genome coverage and heterozygosity for all 1 Mb genome windows. (**D-F**) Mean (± SD) sex differences in genome coverage and heterozygosity per chromosome/scaffold, calculated from the 1 Mb genome windows. Dashed lines mark the genome-wide median across all 1 Mb windows. Data from 1 male and 1 female Eurasian skylarks (*A. arvensis*), analysed using findZX-synteny and zebra finch (*T. guttata*) as a synteny-species reference genome. The previously identified sex chromosomes in this species (Z, 3, 4A, and 5 [11, 12]) are clear outliers. The analysis was performed using a "consensus reference genome".

Guppy (*P. reticulata*)

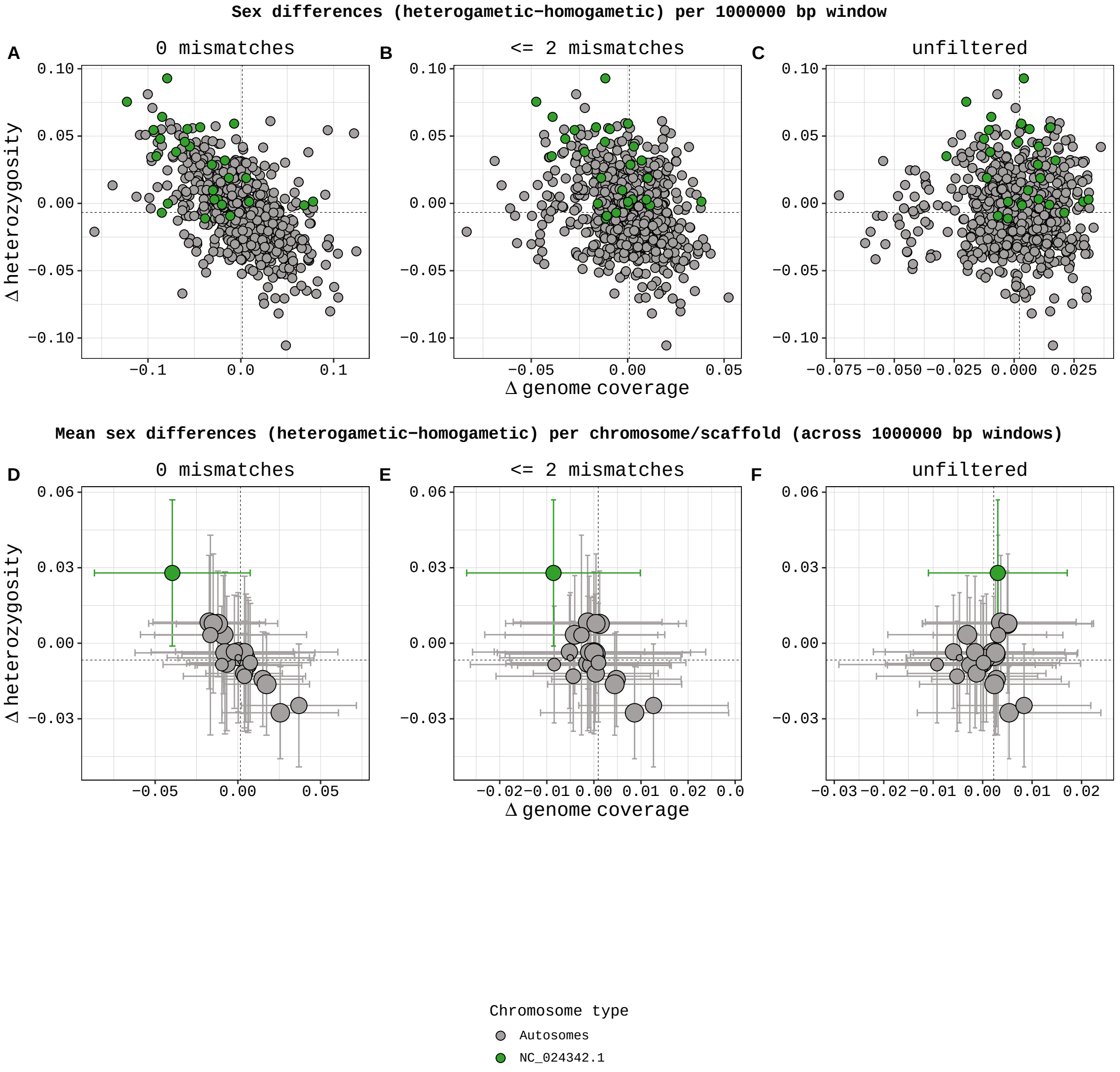

Supplementary Figure 8: (**A-C**) Sex differences in genome coverage and heterozygosity for all 1 Mb genome windows. (**D-F**) Mean (± SD) sex differences in genome coverage and heterozygosity per chromosome/scaffold, calculated from the 1 Mb genome windows. Dashed lines mark the genome-wide median across all 1 Mb windows. Data from 7 male and 16 female guppies (*P. reticulata*), analysed using findZX (without a synteny-species reference genome). The previously identified sex chromosome in this species (Chromosome 12/NC_024342.1 [17, 18]) is a clear outlier.

Central bearded dragon (*P. vitticeps*)

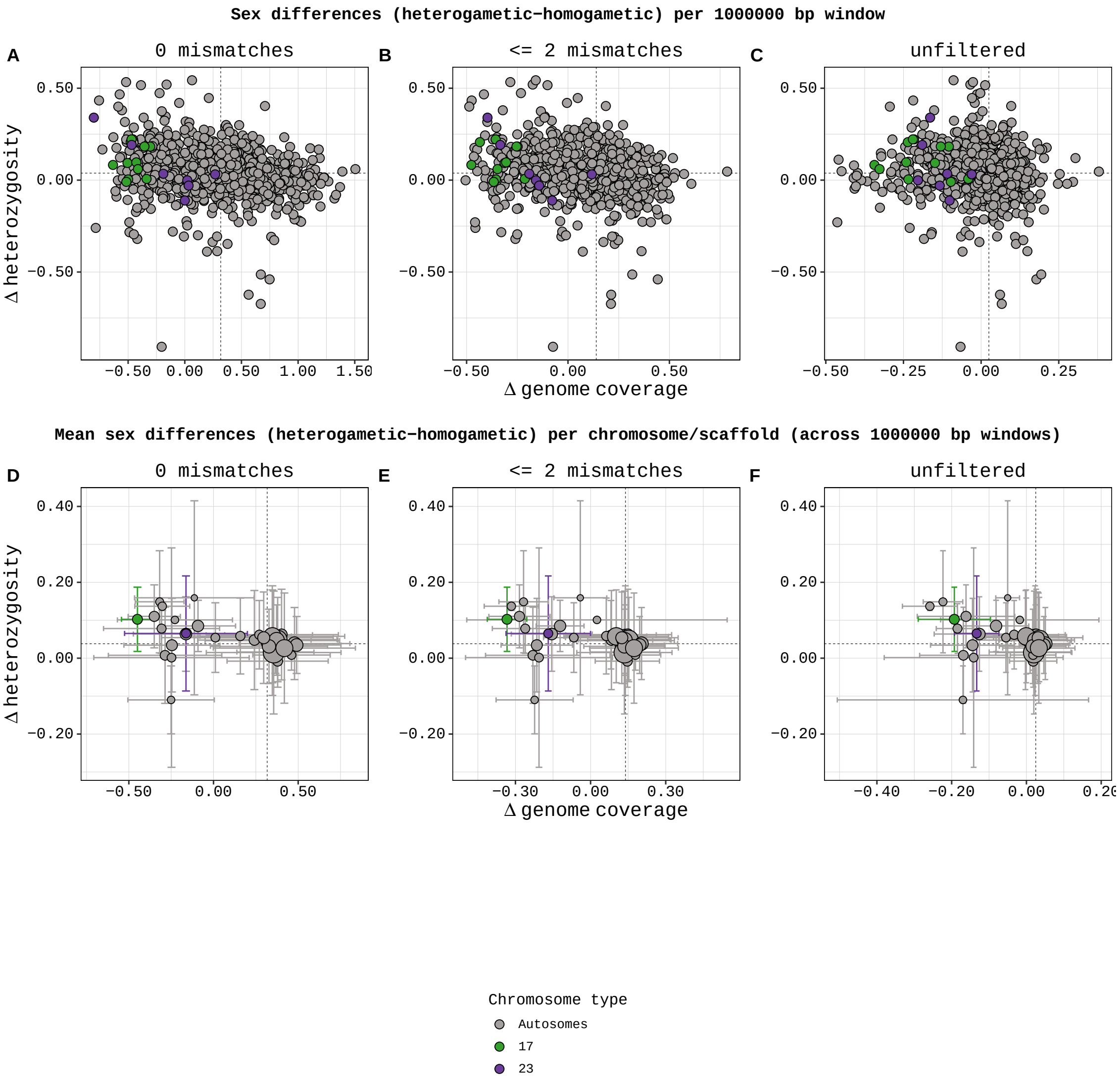

Supplementary Figure 9: (**A-C**) Sex differences in genome coverage and heterozygosity for all 1 Mb genome windows. (**D-F**) Mean (± SD) sex differences in genome coverage and heterozygosity per chromosome/scaffold, calculated from the 1 Mb genome windows. Dashed lines mark the genome-wide median across all 1 Mb windows. Data from 1 male and 1 female (sequenced three times each; see Supplementary Table 2, Additional File 3) central bearded dragons (*P. vitticeps*), analysed using findZX-synteny with the chicken (*G. gallus*) as a synteny-species reference genome. The previously identified sex chromosomes in this species (17 and 23 [19]) are outliers, but not the only ones.

Central bearded dragon (*P. vitticeps*)

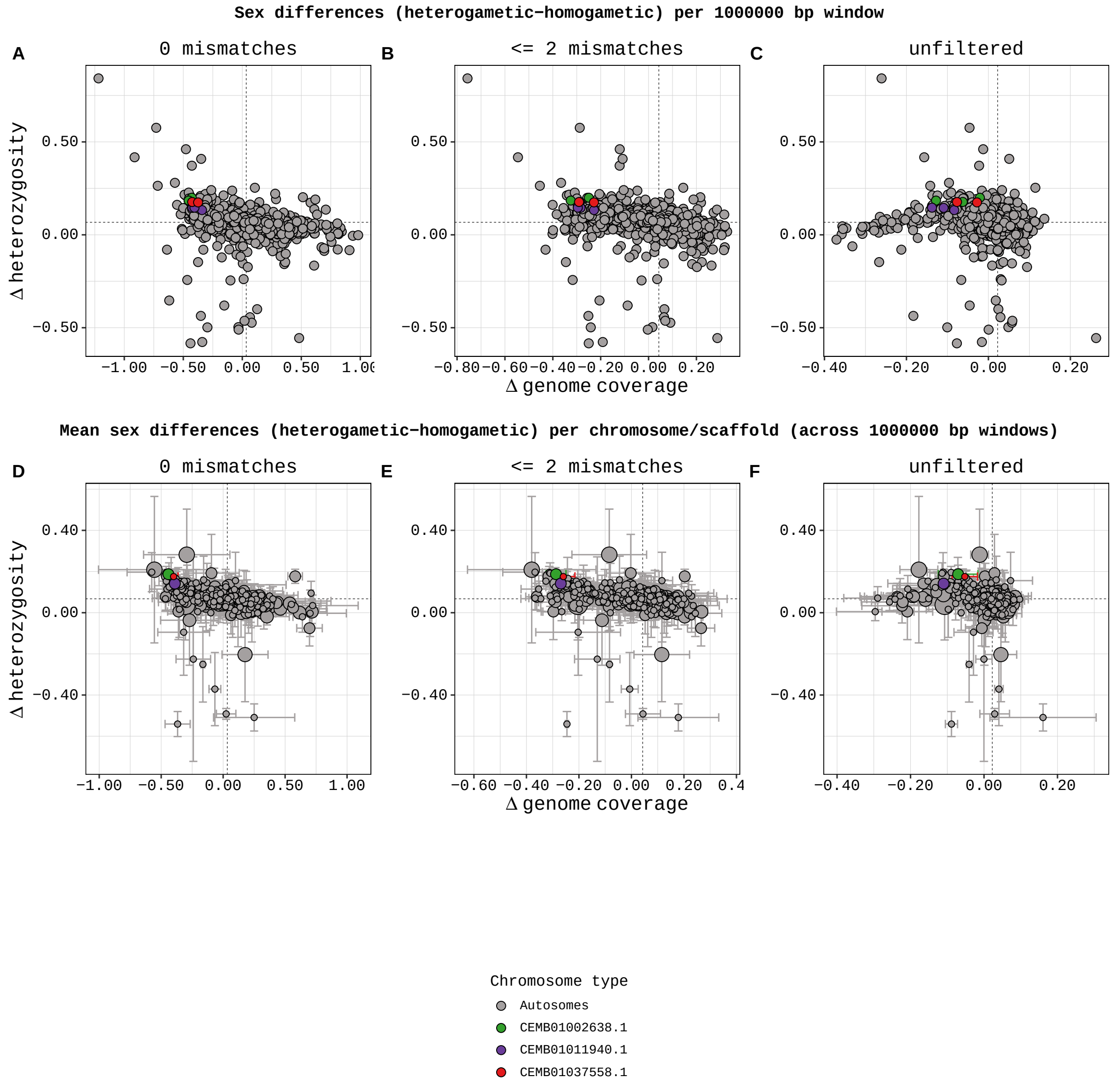

Supplementary Figure 10: (**A-C**) Sex differences in genome coverage and heterozygosity for all 1 Mb genome windows. (**D-F**) Mean (± SD) sex differences in genome coverage and heterozygosity per chromosome/scaffold, calculated from the 1 Mb genome windows. Dashed lines mark the genome-wide median across all 1 Mb windows. Data from 1 male and 1 female (sequenced three times each; see Supplementary Table 2, Additional File 3) central bearded dragons (*P. vitticeps*), analysed using findZX (without a synteny-species reference genome). The previously identified sex chromosomes scaffolds in this species (CEMB01002638.1, CEMB01011940.1 and CEMB01037558.1 [19]) are not clear outliers. The fourth sex-linked scaffold (CEMB01017046.1) is shorter than 1 Mb and not included in this plot.

Ruff (*C. pugnax)*

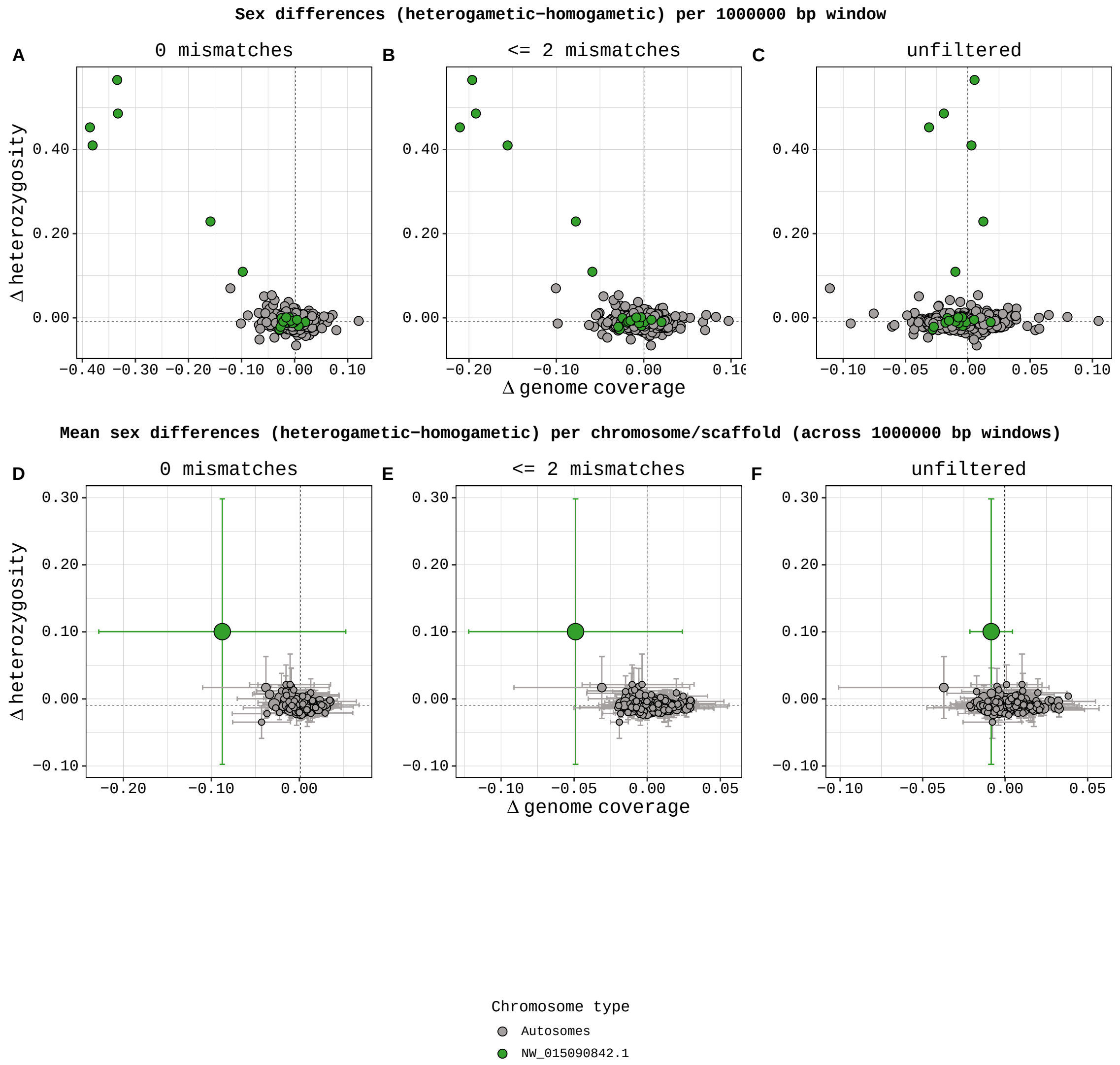

Supplementary Figure 11: (**A-C**) Morph differences in genome coverage and heterozygosity for all 1 Mb genome windows. (**D-F**) Mean (± SD) morph differences in genome coverage and heterozygosity per chromosome/scaffold, calculated from the 1 Mb genome windows. Dashed lines mark the genome-wide median across all 1 Mb windows. Data from 3 male ruffs (*C. pugnax*); 2 faeder males (heterozygotic inversion) and 1 resident male (homozygotic inversion). The data was analysed using findZX (without the use of a synteny-species reference genome). The previously identified scaffold containing the inversion polymorphism (scaffold28/NW_015090842.1 [15]) is a clear outlier.

2.5 Output plots type 4 from analyses with fewer samples

Mantled howler monkey (*A. palliata*), 1 female, 1 male

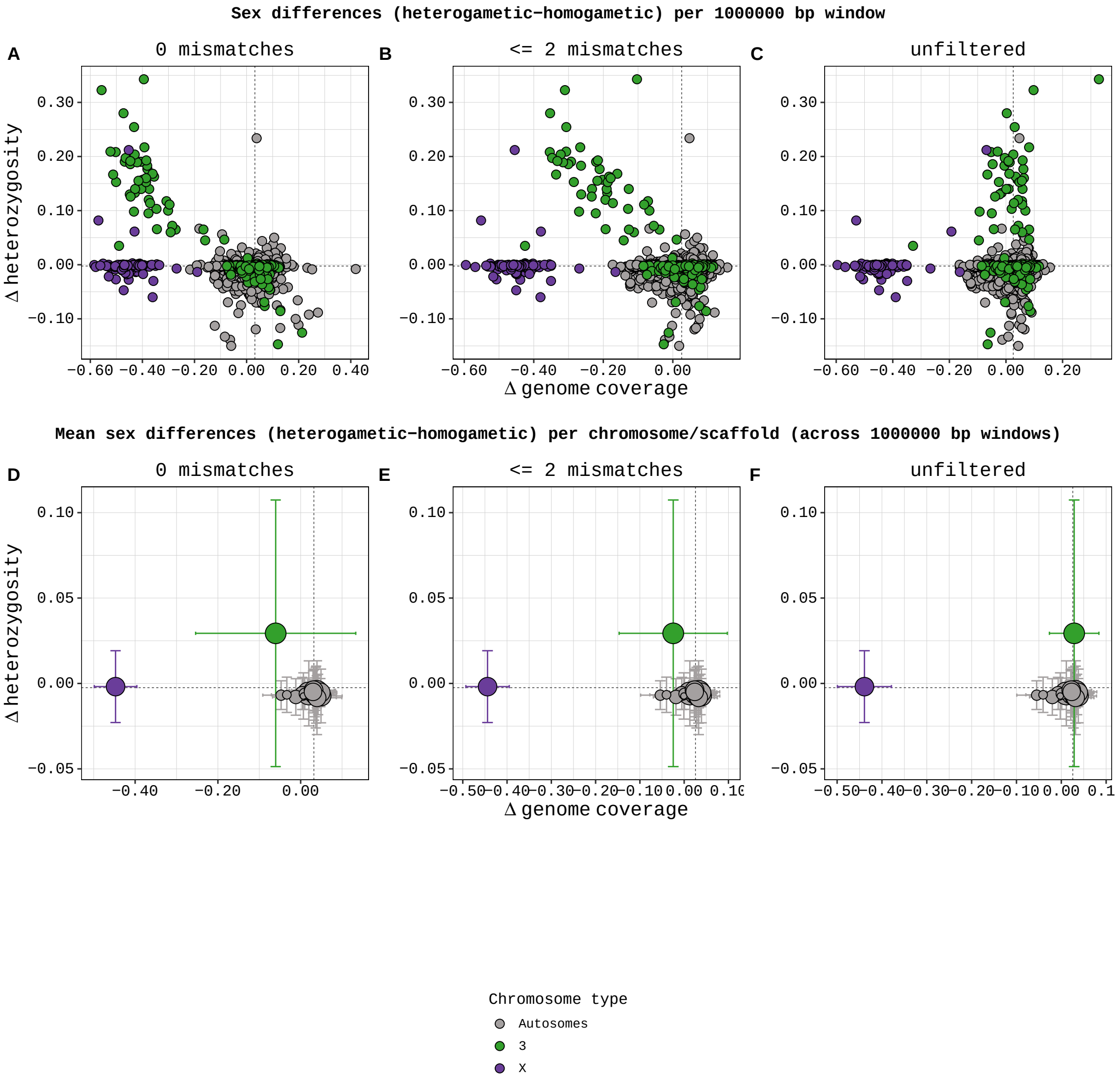

Supplementary Figure 12: (**A-C**) Sex differences in genome coverage and heterozygosity for all 1 Mb genome windows. (**D-F**) Mean (± SD) sex differences in genome coverage and heterozygosity per chromosome/scaffold, calculated from the 1 Mb genome windows. Dashed lines mark the genome-wide median across all 1 Mb windows. Data from 1 male and 1 female mantled howler monkey (*A. palliata*), analysed using findZX-synteny and human (*H. sapiens*) as a synteny-species reference genome. The previously identified sex chromosomes in this species (X and 3 [14]) are clear outliers.

Guppy (*P. reticulata*), 1 female, 1 male

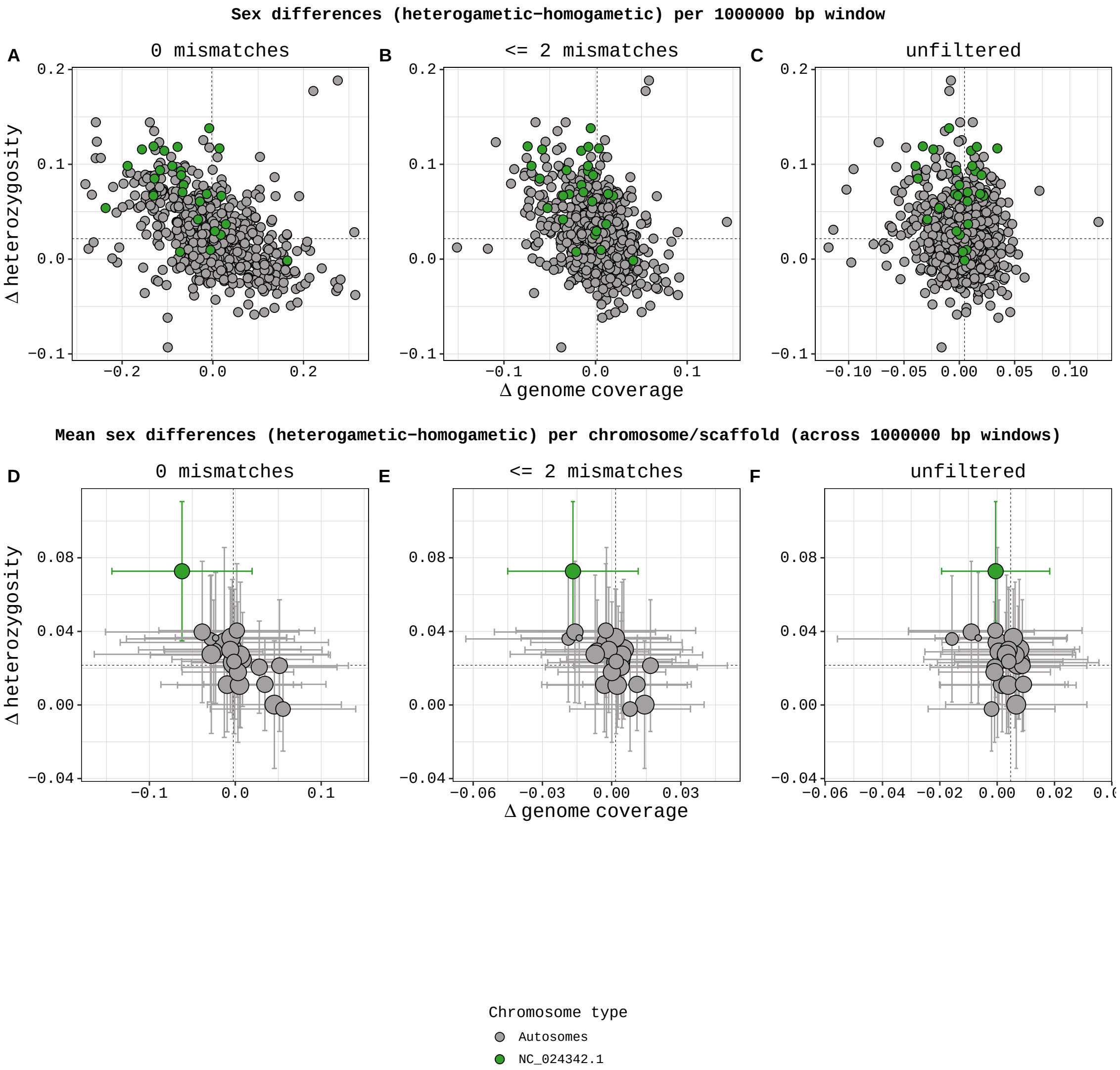

Supplementary Figure 13: (**A-C**) Sex differences in genome coverage and heterozygosity for all 1 Mb genome windows. (**D-F**) Mean (± SD) sex differences in genome coverage and heterozygosity per chromosome/scaffold, calculated from the 1 Mb genome windows. Dashed lines mark the genome-wide median across all 1 Mb windows. Data from 1 male and 1 female guppy (*P. reticulata*), analysed using findZX (without a synteny-species reference genome). The previously identified sex chromosome in this species (Chromosome 12/NC_024342.1 [17, 18]) is a clear outlier.

2.6 Output plots type 4 from analyses of subsampled files

Mantled howler monkey (*A. palliata*), subsampled to 50% of the smallest sample

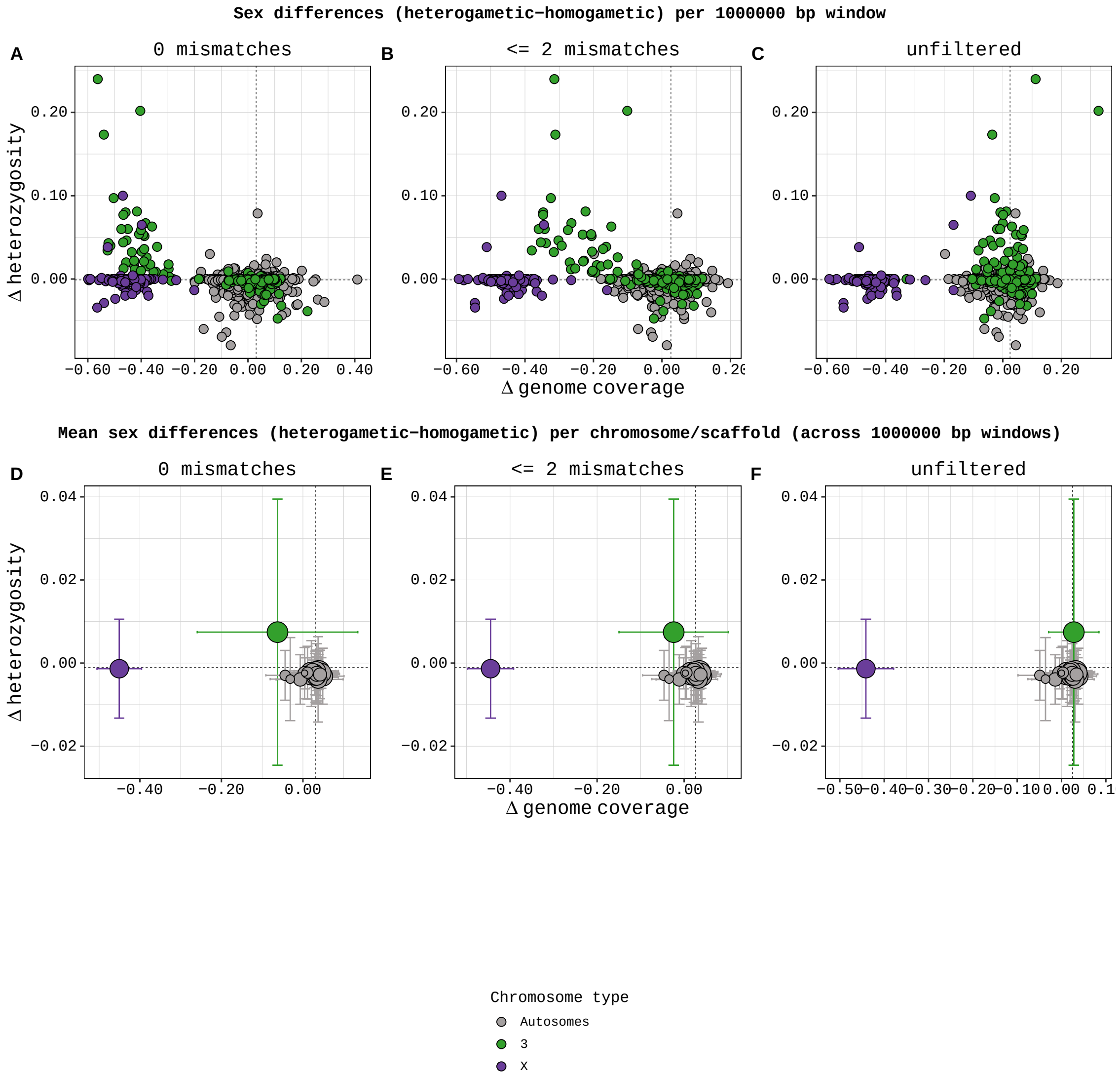

Supplementary Figure 14: (**A-C**) Sex differences in genome coverage and heterozygosity for all 1 Mb genome windows. (**D-F**) Mean (± SD) sex differences in genome coverage and heterozygosity per chromosome/scaffold, calculated from the 1 Mb genome windows. Dashed lines mark the genome-wide median across all 1 Mb windows. Data from 1 male and 1 female mantled howler monkeys (*A. palliata*), subsampled to 50% of the number of base pairs in the smallest of the fastq files. Analysed using findZX-synteny and human (*H. sapiens*) as a synteny-species reference genome. The previously identified sex chromosomes in this species (X and 3 [14]) are clear outliers.

2.7 Output plots type 4 (and 1) from analyses using distantly related species as synteny-species

Mantled howler monkey (*A. palliata*)

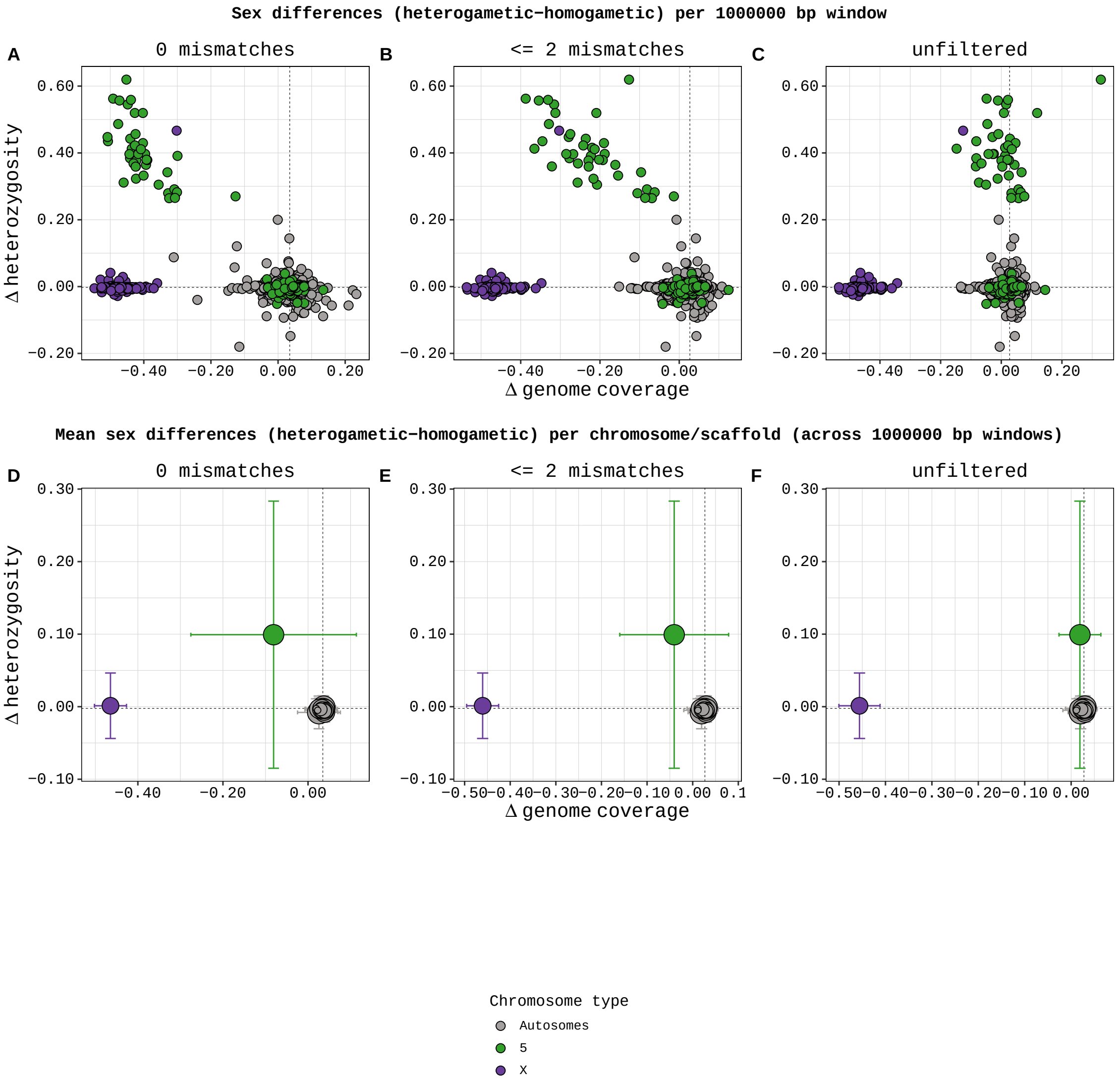

Supplementary Figure 15: (**A-C**) Sex differences in genome coverage and heterozygosity for all 1 Mb genome windows. (**D-F**) Mean (± SD) sex differences in genome coverage and heterozygosity per chromosome/scaffold, calculated from the 1 Mb genome windows. Dashed lines mark the genome-wide median across all 1 Mb windows. Data from 2 male and 2 female mantled howler monkeys (*A. palliata*), analysed using findZX-synteny and meerkat (*S. suricatta*) as a synteny-species reference genome. The previously identified sex chromosomes in this species (X and 5 [14]) are clear outliers.

Eurasian skylark (*A. alauda*)

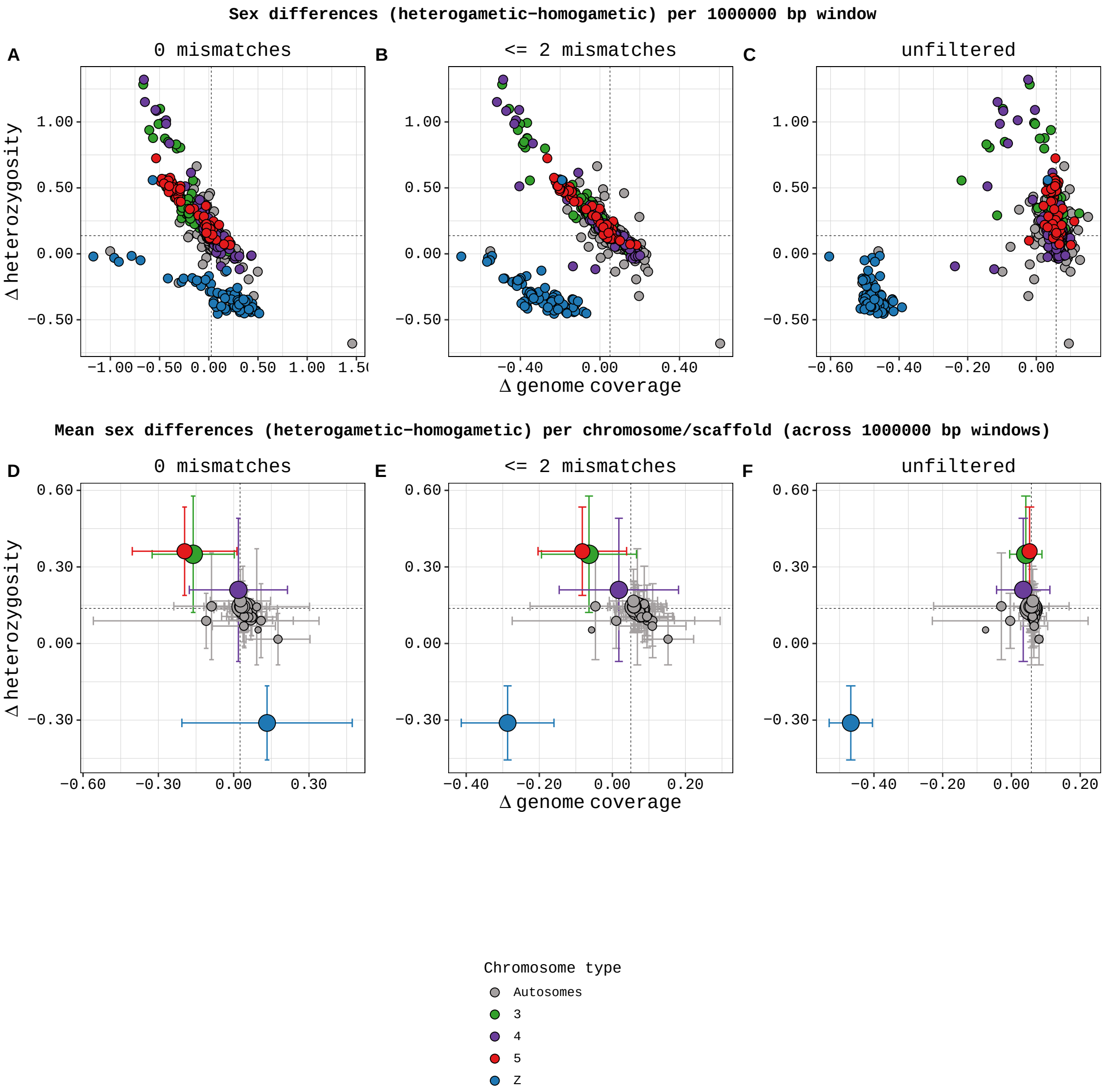

Supplementary Figure 16: (**A-C**) Sex differences in genome coverage and heterozygosity for all 1 Mb genome windows. (**D-F**) Mean (± SD) sex differences in genome coverage and heterozygosity per chromosome/scaffold, calculated from the 1 Mb genome windows. Dashed lines mark the genome-wide median across all 1 Mb windows. Data from 1 male and 1 female Eurasian skylark (*A. arvensis*), analysed with findZX-synteny and chicken (*G. gallus*) as a synteny-species. Three of the four previously identified sex chromosomes in this species are clear outliers (Z, 3 and 5 [11, 12]). Chromosome 4 is not a clear outlier, as a result of the sex-linked part of this chromosome only constituting 10 Mb of this chromosome (91 Mb in total). Supplementary Figure 20 show the same data as in this plot, but across chromosome positions in the chicken genome. In this plot, the beginning of chromosome 4 (which is homologous to chromosome 4A in the zebra finch) show a sex-linked pattern. The analysis was performed using a ”consensus reference genome”.

Eurasian skylark (*A. alauda)*

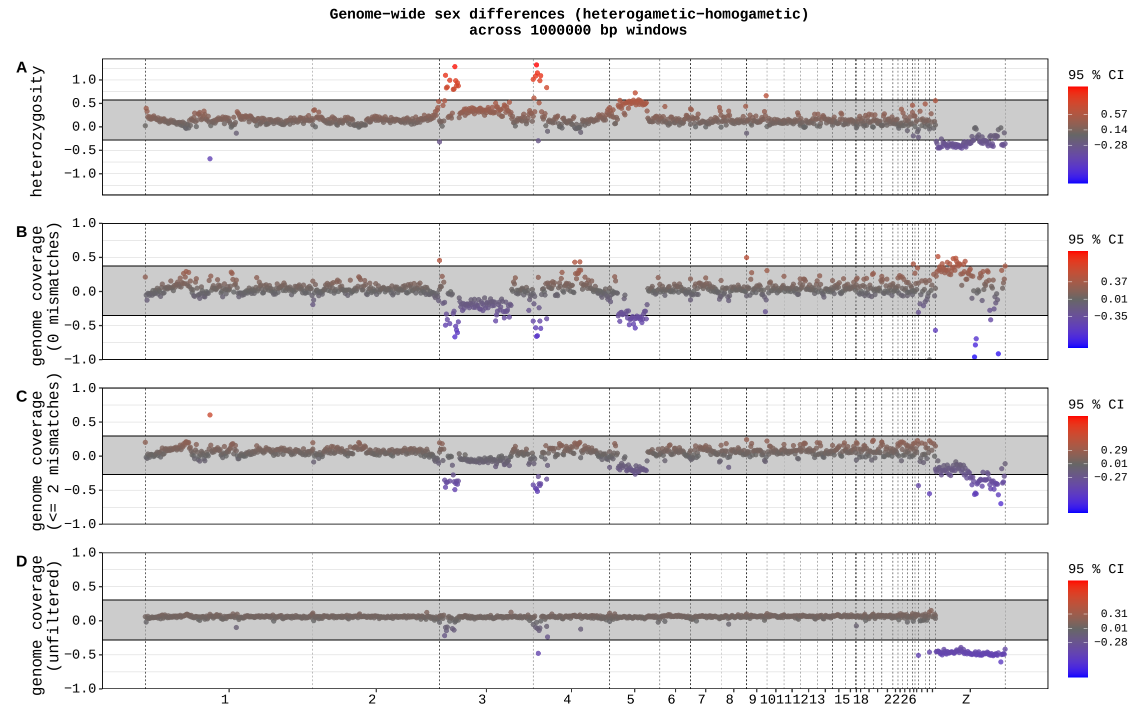

Supplementary Figure 17: Sex differences in genome coverage and heterozygosity values (1 Mb windows) for Eurasian skylark, plotted along chromosome positions in the chicken genome. The four rows show: (**A**) heterozygosity, and genome coverage with (**B**) strict filtering (0 mismatches allowed), (**C**) intermediate filtering ($\leq$2 mismatches) and (**D**) no filtering of mapped reads (unfiltered). The previously identified sex chromosomes in this species (Z, 3, 4 and 5 [11, 12]) are clear outliers. The analysis was performed using a "consensus reference genome".

Ruff (*C. pugnax*)

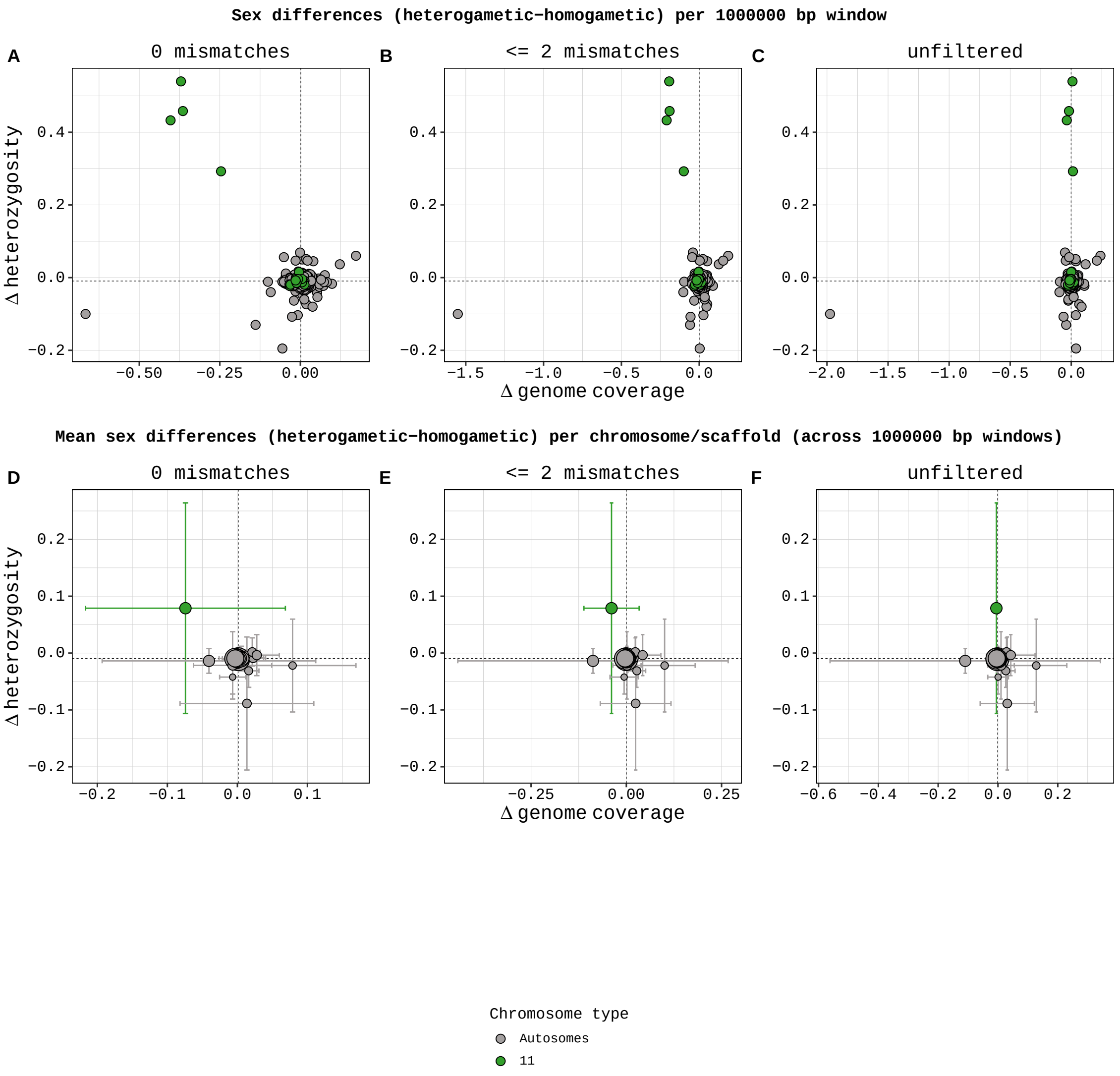

Supplementary Figure 18: (**A-C**) Morph differences in genome coverage and heterozygosity for all 1 Mb genome windows. (**D-F**) Mean (± SD) morph differences in genome coverage and heterozygosity per chromosome/scaffold, calculated from the 1 Mb genome windows. Dashed lines mark the genome-wide median across all 1 Mb windows. Data from 3 male ruffs (*C. pugnax*); 2 faeder males (heterozygotic inversion) and 1 resident male (homozygotic inversion).The data was analysed using findZX-synteny with chicken (*G. gallus*) as a synteny-species reference genome. Chromosome 11, which contain the inversion polymorphism [15], is an outlier.

References

[1] Bolger AM, Lohse M, Usadel B. 2014. Trimmomatic: a flexible trimmer for Illumina sequence data. *Bioinformatics*, 30, 21142120.

[2] Andrews S. 2010. FastQC: A Quality Control Tool for High Throughput Sequence Data [Online]. Available online at: http://www.bioinformatics.babraham.ac.uk/projects/fastqc/

[3] Ewels P, Magnusson M, Lundin S, Kller M. 2016. MultiQC: summarize analysis results for multiple tools and samples in a single report. *Bioinformatics*, 32, 30473048.

[4] Li H, Durbin R. 2009a. Fast and accurate short read alignment with BurrowsWheeler transform. *Bioinformatics*, 25, 17541760.

[5] Li H, Handsaker B, Wysoker A, Fennell T, Ruan J, Homer N, et al. 2009b. The sequence alignment/map format and SAMtools. *Bioinformatics*, 25, 20782079.

[6] Rimmer A, Phan H, Mathieson I. Iqbal Z, Twigg SRF, WGS500 Consortium, et al. 2014. Integrating mapping-, assembly- and haplotype-based approaches for calling variants in clinical sequencing applications. *Nature Genetics*, 46, 912918.

[7] Li H, 2011. Tabix: fast retrieval of sequence features from generic TAB-delimited files. *Bioinformatics*, 27, 718- 719.

[8] Danecek P, Auton A, Abecasis G, Albers CA, Banks E, DePristo MA, et al. 2011. The variant call format and VCFtools. *Bioinformatics*, 27, 21562158.

[9] Quinlan AR, Hall IM. 2010. BEDTools: a flexible suite of utilities for comparing genomic features. *Bioinformatics*, 26, 841842.

[10] Kiełbasa SM, Wan R, Sato K, Horton P, Frith M. C. 2011. Adaptive seeds tame genomic sequence comparison. *Genome Research*, 21, 487493.

[11] Sigeman H, Ponnikas S, Chauhan P, Dierickx E, Brooke MdeL, Hansson B. 2019. Repeated sex chromosome evolution in vertebrates supported by expanded avian sex chromosomes. *Proceedings of the Royal Society B*, 286, 20192051.

[12] Dierickx EG, Sin SYW, van Veelen HPJ, Brooke ML, Liu Y, Edwards SV, Martin SH. 2020. Genetic diversity, demographic history and neo-sex chromosomes in the Critically Endangered Raso lark. *Proceedings of the Royal Society B*, 287:20192613.

[13] Ma N, S, F, Jones T, C, Thorington R, W, Miller A, Morgan L. 1975. Y-Autosome Translocation in the Howler Monkey. *Journal of Medical Primatology*, 4:299-307.

[14] Solari A, J, Rahn M, 2005. I: Fine structure and meiotic behaviour of the male multiple sex chromosomes in the genus Alouatta. *Cytogenetic and Genome Research*, 108:262-267.

[15] Lamichhaney S, Fan G, Widemo F. et al. 2016. Structural genomic changes underlie alternative reproductive strategies in the ruff (*Philomachus pugnax*). *Nature Genetics*, 48, 8488.

[16] Grützner F, Rens W, Tsend-Ayush E, El-Mogharbel N, O'Brien PC, Jones RC, et al. 2004. In the platypus a meiotic chain of ten sex chromosomes shares genes with the bird Z and mammal X chromosomes. *Nature*, 432, 913917.

[17] Künstner A, Hoffmann M, Fraser BA, Kottler VA, Sharma E, et al. 2016 The Genome of the Trinidadian Guppy, *Poecilia reticulata*, and Variation in the Guanapo Population. *PLOS ONE*, 11: e0169087

[18] Wright AE, Darolti I, Bloch NI, Oostra V, Sandkam B, Buechel SD, et al. 2017. Convergent recombination suppression suggests role of sexual selection in guppy sex chromosome formation. *Nature Communications*, 8, 14251.

[19] Deakin JE, Edwards MJ, Patel H, et al. 2016. Anchoring genome sequence to chromosomes of the central bearded dragon (*Pogona vitticeps*) enables reconstruction of ancestral squamate macrochromosomes and identifies sequence content of the Z chromosome. *BMC Genomics*, 17:447.

Phylopic/Wikimedia commons credits:

The guppy silhouette (Figure 5d) was created by Josefine Bohr Brask and shared via phylopic.org under a CC By 3.0 license (https://creativecommons.org/licenses/by/3.0/). Not modified from its original state.

The ruff silhouette (Figure 5f) was created by Alexandre Vong and shared via phylopic.org under a CC By 3.0 license (https://creativecommons.org/licenses/by/3.0/). Not modified from its original state.

Silhouettes in Figure 5a-c,e were downloaded from phylopic.org and were without copyright restrictions.
