# Supplementary material for: Findzx: an automated pipeline for detecting and visualising sex chromosomes using whole-genome sequencing data
Source: BMC Genomics. 2022 Apr 27;23:328. doi: 10.1186/s12864-022-08432-9 (PMC9044604; doi:10.1186/s12864-022-08432-9)
Supplement: Supplementary file 6 — Additional file 6. Supplementary Table 5. [file 12864_2022_8432_MOESM6_ESM.html]

Snakemake Report


Loading Snakemake Report...

Please enable Javascript in your browser to see this report.

Loading 6.2 MB. For large reports, this can take a while.

Snakemake Report

- Fri Feb 11 17:20:42 2022 CET
- Snakemake 6.4.0

- Workflow (current)
- Statistics

##### Results

- 00 MultiQC reports
- 01 Sample and reference genome statistics
- Output plot type 1 (genome-wide sex differences)
- Output plot type 2 (genome-wide sexes separately)
- Output plot type 3 (scatter plots with chromosome/scaffold length)
- Output plot type 4 (scatter plots)
- Output plot type 5 ("confirm sexing")
- Output tables


## Workflow

This HTML document describe output from the findZX pipeline. (a) This page includes settings specified in the control file, and (b) descriptions of all files listed in this document.

- For details on methodology, see preprint:
- Instructions on how to run findZX is found on the GitHub page:
- [findZX] refers to if the following snakemake file was used for the analysis: snakemake -s workflow/findZX
- [findZX-synteny] refers to if the following snakemake file was used for the analysis: snakemake -s workflow/findZX-synteny

---

# Configuration file settings

**General settings:**

- findZX analysis run name: Alouatta\_palliata
- Analysed samples are listed here: config/9\_species\_config/Alouatta\_palliata\_units.tsv
- Reference genome: ../data/external\_raw/genome/AloPal\_v1.fasta
- Window sizes (bp): [1000000]
- Mismatch settings: [0.0, 0.2, 'unfiltered']
- Minimum size of scaffolds to plot: 10000 bp
- Fastq files were trimmed with settings ['LEADING:3', 'TRAILING:3', 'SLIDINGWINDOW:4:15', 'MINLEN:36', 'ILLUMINACLIP:workflow/meta/adapters/TruSeq3-PE.fa:2:30:10']

**Settings for plotting [findZX]:**

- List of chromosomes/scaffolds for plotting: (None)

**Settings for plotting [findZX-synteny]:**

- List of chromosomes/scaffolds for plotting: (config/9\_species\_config/chromosome\_files/HS\_chromosomes.list)
- List of chromosomes/scaffolds to highlight (Plot type 4 only): (['X', 3])

---

# Results section content

This section describe the output files that are attached to this HTML report.

**Quality control:**

00 MultiQC reports:

- MultiQC reports for trimmed and untrimmed fastq files

01 Sample and reference genome statistics:

- Reference genome statistics (calculated with Assembly stats)
- Per sample heterozygosity values (total number of heterozygous sites divided by genome length)
- [findZX-synteny]: Proportion (value between 0 and 1) of 5kb windows in the study-species reference genome that was sucessfully matched with the synteny-species reference genome.

**Output plots:**

All output plots are multi-page PDF files, where the last page contain information on what file was used to produce each plot.

[findZX] Output plots location: results/Alouatta\_palliata/no\_synteny/plots

[findZX-synteny] Output plots location: results/Alouatta\_palliata/synteny/HS/plots

Output plot type 1 (genome-wide sex differences):

- Files (one for each selected window size): 1\_sexDifferences.genomeWide.[1000000]bp.window.pdf
- Description: Per-sex differences in (A) heterozygosity and (B-D) genome coverage. Calculated along chromosome/scaffold positions according to the selected window sizes (bp). By default, the 50 largest scaffolds are plotted. If a list of chromosomes/scaffolds are provided in the config file ("chr\_file" or "synteny\_chr\_file"), these are plotted.

Output plot type 2 (genome-wide sexes separately):

- Files (one for each selected window size): 1\_sexesSeparate.genomeWide.[1000000]bp.window.pdf
- Description: Per-sex (A) heterozygosity and (B-D) genome coverage values (mean value for homogametic samples in purple, heterogametic samples in blue). Calculated along chromosome/scaffold positions according to the selected window sizes (bp). By default, the 50 largest scaffolds are plotted. If a list of chromosomes/scaffolds are provided in the config file ("chr\_file" or "synteny\_chr\_file"), these are plotted.

Output plot type 3 (scatter plots with chromosome/scaffold length):

- File name: 3\_sexDifferences.chromosome.pdf
- Description: Scatter plots showing between-sex genome coverage and percentage of heterozygosity differences, and scaffold/chromosome length (bp). This plot is especially useful for highly fragmented reference genomes.

Output plot type 4 (scatter plots):

- Files (one for each selected window size): 4\_sexDifferences.[1000000]bp.window.highlight.pdf
- Description: Scatter plots showing between-sex genome coverage and percentage of heterozygosity differences. Dashed lines mark the genome-wide median across all genome windows. The first set of plots (page 1: A-C) show sex differences in genome coverage and heterozygosity across genome window. The second set of plots (page 2:D-F) show mean (± standard deviation) sex differences in genome coverage and heterozygosity per chromosome/scaffold, calculated from the genome windows from A-C.

Output plot type 5 ("confirm sexing"):

- Files (one for each selected mismatch setting): 5\_confirmSexing.samplesSeparately.mismatch.[0.0, 0.2, 'unfiltered'].pdf
- These plot are based on per-individual coverage and heterozygosity values for all 5 kb windows, and can be used to (a) confirm, or identify mistakes, in the sexing of invididuals and to (b) identify samples where the alignment was not successful (i.e. low coverage).
- Description: Histogram of (A) genome coverage and (B) heterozygosity per 5 kb window. (C) Heatmap of genome coverage and heterozygosity values per 5 kb window. Heatmaps of (D) genome coverage and (E) heterozygosity (on the x-axis) and scaffold length (on the y-axis). (F) Heatmap of genome coverage values along the 50 largest chromosomes/scaffolds (or a selected list of chromosomes/scaffolds).

---

**Output tables:**

Selected Output tables are attached to this HTML file. All output tables are found under the results directory.

[findZX] Output tables location: results/Alouatta\_palliata/no\_synteny/tables

[findZX-synteny] Output tables location: results/Alouatta\_palliata/synteny/HS/tables

For every selected window size, the following tables are attached:

- Genome windows where genome coverage is significantly different between sexes (outside 95% CI, one file for each mismatch setting): diffGenomeCoverage.mismatch.[0.0, 0.2, 'unfiltered'].[1000000]bp.outlier.out

  > - Outliers are red and blue data points in Output plot type 1 (genome-wide sex differences)
- Genome windows where heterozygosity is significantly different between sexes (outside 95% CI): diffHeterozygosity.[1000000]bp.outlier.out panels B-D.

  > - Outliers are red and blue data points in Output plot type 1 (genome-wide sex differences) panel A.
- Mean and standard deviation values per chromosome/scaffold : sexDifferences\_mean\_std\_dev.[1000000]bp.window.tsv

  > - Same values as panels D-F in Output plot type 4 (scatter plots)

Click the nodes to obtain details about each step.

## 00 MultiQC reports

## 01 Sample and reference genome statistics

## Output plot type 1 (genome-wide sex differences)

#### window size: 1000000 bp

## Output plot type 2 (genome-wide sexes separately)

#### window size: 1000000 bp

## Output plot type 3 (scatter plots with chromosome/scaffold length)

## Output plot type 4 (scatter plots)

#### window size: 1000000 bp

## Output plot type 5 ("confirm sexing")

#### mismatch option: 0.0


#### mismatch option: 0.2


#### mismatch option: unfiltered

## Output tables

#### window size: 1000000 bp

## Statistics

If the workflow has been executed in cluster/cloud, runtimes include the waiting time in the queue.

Loading...

##### 

×

Download original

##### Rule multiqc

×

Rule properties

|  |  |
| --- | --- |
| Jobs | 1 |
| Input files | |
| - results/Alouatta\_palliata/qc/fastqc/SRR9655168\_\_homogametic.untrimmed\_fastqc.zip - results/Alouatta\_palliata/qc/fastqc/SRR9655169\_\_homogametic.untrimmed\_fastqc.zip - results/Alouatta\_palliata/qc/fastqc/SRR9655170\_\_heterogametic.untrimmed\_fastqc.zip - results/Alouatta\_palliata/qc/fastqc/SRR9655171\_\_heterogametic.untrimmed\_fastqc.zip | |
| Output files | |
| - results/Alouatta\_palliata/qc/fastqc/multiqc.untrimmed.html | |
| Conda software stack | |
| - multiqc ==1.10.1 | |
| Code | |
| |  |  | | --- | --- | | ``` 1 ``` | ``` multiqc {input} -n {params} -f ``` | | |

##### Rule fastqc

×

Rule properties

|  |  |
| --- | --- |
| Jobs | 4 |
| Input files | |
|  | |
| Output files | |
| - results/Alouatta\_palliata/qc/fastqc/{sample,SRR9655168|SRR9655169|SRR9655170|SRR9655171}\_\_{group,homogametic|homogametic|heterogametic|heterogametic}.untrimmed.html - results/Alouatta\_palliata/qc/fastqc/{sample,SRR9655168|SRR9655169|SRR9655170|SRR9655171}\_\_{group,homogametic|homogametic|heterogametic|heterogametic}.untrimmed\_fastqc.zip | |
| Conda software stack | |
| - fastqc ==0.11.9 | |
| Code | |
| |  |  | | --- | --- | | ```  1  2  3  4  5  6  7  8  9 10 11 12 13 14 15 16 17 18 19 20 21 22 23 24 25 26 27 28 29 30 31 32 33 34 35 36 37 38 39 40 41 42 43 44 45 46 47 48 49 50 51 52 ``` | ``` """Snakemake wrapper for fastqc."""  __author__ = "Julian de Ruiter" __copyright__ = "Copyright 2017, Julian de Ruiter" __email__ = "julianderuiter@gmail.com" __license__ = "MIT"   from os import path import re from tempfile import TemporaryDirectory  from snakemake.shell import shell  log = snakemake.log_fmt_shell(stdout=True, stderr=True)   def basename_without_ext(file_path):     """Returns basename of file path, without the file extension."""      base = path.basename(file_path)     # Remove file extension(s) (similar to the internal fastqc approach)     base = re.sub("\\.gz$", "", base)     base = re.sub("\\.bz2$", "", base)     base = re.sub("\\.txt$", "", base)     base = re.sub("\\.fastq$", "", base)     base = re.sub("\\.fq$", "", base)     base = re.sub("\\.sam$", "", base)     base = re.sub("\\.bam$", "", base)      return base   # Run fastqc, since there can be race conditions if multiple jobs # use the same fastqc dir, we create a temp dir. with TemporaryDirectory() as tempdir:     shell(         "fastqc {snakemake.params} -t {snakemake.threads} "         "--outdir {tempdir:q} {snakemake.input[0]:q}"         " {log}"     )      # Move outputs into proper position.     output_base = basename_without_ext(snakemake.input[0])     html_path = path.join(tempdir, output_base + "_fastqc.html")     zip_path = path.join(tempdir, output_base + "_fastqc.zip")      if snakemake.output.html != html_path:         shell("mv {html_path:q} {snakemake.output.html:q}")      if snakemake.output.zip != zip_path:         shell("mv {zip_path:q} {snakemake.output.zip:q}") ``` | | |

##### Rule multiqc\_2

×

Rule properties

|  |  |
| --- | --- |
| Jobs | 1 |
| Input files | |
| - results/Alouatta\_palliata/qc/fastqc/SRR9655168\_\_homogametic.trimmed\_fastqc.zip - results/Alouatta\_palliata/qc/fastqc/SRR9655169\_\_homogametic.trimmed\_fastqc.zip - results/Alouatta\_palliata/qc/fastqc/SRR9655170\_\_heterogametic.trimmed\_fastqc.zip - results/Alouatta\_palliata/qc/fastqc/SRR9655171\_\_heterogametic.trimmed\_fastqc.zip | |
| Output files | |
| - results/Alouatta\_palliata/qc/fastqc/multiqc.trimmed.html | |
| Conda software stack | |
| - multiqc ==1.10.1 | |
| Code | |
| |  |  | | --- | --- | | ``` 1 ``` | ``` multiqc {input} -n {params} -f ``` | | |

##### Rule fastqc\_2

×

Rule properties

|  |  |
| --- | --- |
| Jobs | 4 |
| Input files | |
|  | |
| Output files | |
| - results/Alouatta\_palliata/qc/fastqc/{sample,SRR9655168|SRR9655169|SRR9655170|SRR9655171}\_\_{group,homogametic|homogametic|heterogametic|heterogametic}.trimmed.html - results/Alouatta\_palliata/qc/fastqc/{sample,SRR9655168|SRR9655169|SRR9655170|SRR9655171}\_\_{group,homogametic|homogametic|heterogametic|heterogametic}.trimmed\_fastqc.zip | |
| Conda software stack | |
| - fastqc ==0.11.9 | |
| Code | |
| |  |  | | --- | --- | | ```  1  2  3  4  5  6  7  8  9 10 11 12 13 14 15 16 17 18 19 20 21 22 23 24 25 26 27 28 29 30 31 32 33 34 35 36 37 38 39 40 41 42 43 44 45 46 47 48 49 50 51 52 ``` | ``` """Snakemake wrapper for fastqc."""  __author__ = "Julian de Ruiter" __copyright__ = "Copyright 2017, Julian de Ruiter" __email__ = "julianderuiter@gmail.com" __license__ = "MIT"   from os import path import re from tempfile import TemporaryDirectory  from snakemake.shell import shell  log = snakemake.log_fmt_shell(stdout=True, stderr=True)   def basename_without_ext(file_path):     """Returns basename of file path, without the file extension."""      base = path.basename(file_path)     # Remove file extension(s) (similar to the internal fastqc approach)     base = re.sub("\\.gz$", "", base)     base = re.sub("\\.bz2$", "", base)     base = re.sub("\\.txt$", "", base)     base = re.sub("\\.fastq$", "", base)     base = re.sub("\\.fq$", "", base)     base = re.sub("\\.sam$", "", base)     base = re.sub("\\.bam$", "", base)      return base   # Run fastqc, since there can be race conditions if multiple jobs # use the same fastqc dir, we create a temp dir. with TemporaryDirectory() as tempdir:     shell(         "fastqc {snakemake.params} -t {snakemake.threads} "         "--outdir {tempdir:q} {snakemake.input[0]:q}"         " {log}"     )      # Move outputs into proper position.     output_base = basename_without_ext(snakemake.input[0])     html_path = path.join(tempdir, output_base + "_fastqc.html")     zip_path = path.join(tempdir, output_base + "_fastqc.zip")      if snakemake.output.html != html_path:         shell("mv {html_path:q} {snakemake.output.html:q}")      if snakemake.output.zip != zip_path:         shell("mv {zip_path:q} {snakemake.output.zip:q}") ``` | | |

##### Rule trim\_reads\_pe

×

Rule properties

|  |  |
| --- | --- |
| Jobs | 4 |
| Input files | |
|  | |
| Output files | |
| - results/Alouatta\_palliata/trimmed/{sample,SRR9655168|SRR9655169|SRR9655170|SRR9655171}\_\_{group,homogametic|homogametic|heterogametic|heterogametic}.1.fastq.gz - results/Alouatta\_palliata/trimmed/{sample,SRR9655168|SRR9655169|SRR9655170|SRR9655171}\_\_{group,homogametic|homogametic|heterogametic|heterogametic}.2.fastq.gz - results/Alouatta\_palliata/trimmed/{sample,SRR9655168|SRR9655169|SRR9655170|SRR9655171}\_\_{group,homogametic|homogametic|heterogametic|heterogametic}.1.unpaired.fastq.gz - results/Alouatta\_palliata/trimmed/{sample,SRR9655168|SRR9655169|SRR9655170|SRR9655171}\_\_{group,homogametic|homogametic|heterogametic|heterogametic}.2.unpaired.fastq.gz | |
| Conda software stack | |
| - trimmomatic ==0.36 - pigz ==2.3.4 - snakemake-wrapper-utils ==0.1.3 | |
| Code | |
| |  |  | | --- | --- | | ```   1   2   3   4   5   6   7   8   9  10  11  12  13  14  15  16  17  18  19  20  21  22  23  24  25  26  27  28  29  30  31  32  33  34  35  36  37  38  39  40  41  42  43  44  45  46  47  48  49  50  51  52  53  54  55  56  57  58  59  60  61  62  63  64  65  66  67  68  69  70  71  72  73  74  75  76  77  78  79  80  81  82  83  84  85  86  87  88  89  90  91  92  93  94  95  96  97  98  99 100 101 102 103 ``` | ``` """ bio/trimmomatic/pe  Snakemake wrapper to trim reads with trimmomatic in PE mode with help of pigz. pigz is the parallel implementation of gz. Trimmomatic spends most of the time compressing and decompressing instead of trimming sequences. By using process substitution (<(command), >(command)), we can accelerate trimmomatic a lot. Consider providing this wrapper with at least 1 extra thread per each gzipped input or output file. """  __author__ = "Johannes Köster, Jorge Langa" __copyright__ = "Copyright 2016, Johannes Köster" __email__ = "koester@jimmy.harvard.edu" __license__ = "MIT"   from snakemake.shell import shell from snakemake_wrapper_utils.java import get_java_opts  # Distribute available threads between trimmomatic itself and any potential pigz instances def distribute_threads(input_files, output_files, available_threads):     gzipped_input_files = sum(1 for file in input_files if file.endswith(".gz"))     gzipped_output_files = sum(1 for file in output_files if file.endswith(".gz"))     potential_threads_per_process = available_threads // (         1 + gzipped_input_files + gzipped_output_files     )     if potential_threads_per_process > 0:         # decompressing pigz creates at most 4 threads         pigz_input_threads = (             min(4, potential_threads_per_process) if gzipped_input_files != 0 else 0         )         pigz_output_threads = (             (available_threads - pigz_input_threads * gzipped_input_files)             // (1 + gzipped_output_files)             if gzipped_output_files != 0             else 0         )         trimmomatic_threads = (             available_threads             - pigz_input_threads * gzipped_input_files             - pigz_output_threads * gzipped_output_files         )     else:         # not enough threads for pigz         pigz_input_threads = 0         pigz_output_threads = 0         trimmomatic_threads = available_threads     return trimmomatic_threads, pigz_input_threads, pigz_output_threads   def compose_input_gz(filename, threads):     if filename.endswith(".gz") and threads > 0:         return "<(pigz -p {threads} --decompress --stdout {filename})".format(             threads=threads, filename=filename         )     return filename   def compose_output_gz(filename, threads, compression_level):     if filename.endswith(".gz") and threads > 0:         return ">(pigz -p {threads} {compression_level} > {filename})".format(             threads=threads, compression_level=compression_level, filename=filename         )     return filename   extra = snakemake.params.get("extra", "") java_opts = get_java_opts(snakemake) log = snakemake.log_fmt_shell(stdout=True, stderr=True) compression_level = snakemake.params.get("compression_level", "-5") trimmer = " ".join(snakemake.params.trimmer)  # Distribute threads input_files = [snakemake.input.r1, snakemake.input.r2] output_files = [     snakemake.output.r1,     snakemake.output.r1_unpaired,     snakemake.output.r2,     snakemake.output.r2_unpaired, ]  trimmomatic_threads, input_threads, output_threads = distribute_threads(     input_files, output_files, snakemake.threads )  input_r1, input_r2 = [     compose_input_gz(filename, input_threads) for filename in input_files ]  output_r1, output_r1_unp, output_r2, output_r2_unp = [     compose_output_gz(filename, output_threads, compression_level)     for filename in output_files ]  shell(     "trimmomatic PE -threads {trimmomatic_threads} {java_opts} {extra} "     "{input_r1} {input_r2} "     "{output_r1} {output_r1_unp} "     "{output_r2} {output_r2_unp} "     "{trimmer} "     "{log}" ) ``` | | |

##### Rule confirm\_sexing

×

Rule properties

|  |  |
| --- | --- |
| Jobs | 3 |
| Input files | |
| - results/Alouatta\_palliata/synteny\_lastal/HS/gencov.mismatch.{ED}.small.out - results/Alouatta\_palliata/synteny\_lastal/HS/heterozygosity.bestMatch.small - results/ref/AloPal\_v1/dedup/SRR9655168\_\_homogametic.sorted.dedup.mismatch.{ED}.samtools.stats.txt - results/ref/AloPal\_v1/dedup/SRR9655169\_\_homogametic.sorted.dedup.mismatch.{ED}.samtools.stats.txt - results/ref/AloPal\_v1/dedup/SRR9655170\_\_heterogametic.sorted.dedup.mismatch.{ED}.samtools.stats.txt - results/ref/AloPal\_v1/dedup/SRR9655171\_\_heterogametic.sorted.dedup.mismatch.{ED}.samtools.stats.txt | |
| Output files | |
| - results/Alouatta\_palliata/output/synteny/HS/plots/.misc/read\_length.sorted.nodup.mismatch.{ED}.csv - results/Alouatta\_palliata/output/synteny/HS/plots/5\_confirmSexing.samplesSeparately.mismatch.{ED}.pdf | |
| Conda software stack | |
| - r-doby=4.6.6 - r-data.table=1.14.0 - r-ggplot2=3.3.3 - r-plot3d=1.3 - r-cowplot=1.1.1 - r-viridislite=0.4.0 - r-gridGraphics=0.5\_1 - r-plotly=4.9.3 - tk=8.6.10 - r-tcltk2=1.2\_11 - imagemagick=7.0.11\_14 - r-tidyverse=1.2.1 - r-ggextra=0.8 - r-ggpubr=0.4.0 | |
| Code | |
| |  |  | | --- | --- | | ``` 1 2 3 4 ``` | ```         python workflow/scripts/read_length.py <(for FILE in $(ls {params.map_dir}); do echo "${{FILE##*/}}"; grep "average length" $FILE; done) > {output.read_length}          Rscript workflow/scripts/histogram_indv.R {input.gencov} {input.het} {output.read_length} {output.gencov_het} synteny {params.chromosomes} {params.hetero} {params.homo} 2> {log} ``` | | |

##### Rule matchScaffold2Chr\_cov

×

Rule properties

|  |  |
| --- | --- |
| Jobs | 3 |
| Input files | |
| - results/Alouatta\_palliata/synteny\_lastal/HS/bestMatch.list - results/Alouatta\_palliata/coverage/gencov.mismatch.{ED}.out - results/Alouatta\_palliata/coverage/gencov.mismatch.{ED}.norm.sexAverage.out | |
| Output files | |
| - results/Alouatta\_palliata/synteny\_lastal/HS/gencov.mismatch.{ED}.out - results/Alouatta\_palliata/synteny\_lastal/HS/gencov.mismatch.{ED}.small.out - results/Alouatta\_palliata/synteny\_lastal/HS/gencov.mismatch.{ED}.norm.sexAverage.out - results/Alouatta\_palliata/synteny\_lastal/HS/gencov.mismatch.{ED}.norm.sexAverage.small.out | |
| Conda software stack | |
| - bedtools=2.29.0 | |
| Code | |
| |  |  | | --- | --- | | ``` 1 2 3 4 5 ``` | ```         bedtools intersect -a {input.bestMatch} -b {input.cov} -wa -wb > {output.bestMatch}         cut -f 8,9,10,14- {output.bestMatch} > {output.bestMatch_small}         bedtools intersect -a {input.bestMatch} -b {input.cov_sexAverage} -wa -wb > {output.bestMatch_sexAverage}         cut -f 8,9,10,14- {output.bestMatch_sexAverage} > {output.bestMatch_small_sexAverage} ``` | | |

##### Rule matchScaffold2Chr

×

Rule properties

|  |  |
| --- | --- |
| Jobs | 1 |
| Input files | |
| - results/Alouatta\_palliata/synteny\_lastal/HS/HS\_align\_converted - results/Alouatta\_palliata/coverage/genome\_5kb\_windows.out | |
| Output files | |
| - results/Alouatta\_palliata/synteny\_lastal/HS/genome\_windows.out - results/Alouatta\_palliata/synteny\_lastal/HS/bestMatch.list - results/Alouatta\_palliata/synteny\_lastal/HS/bestMatch.status | |
| Conda software stack | |
| - bedtools=2.29.0 | |
| Code | |
| |  |  | | --- | --- | | ```  1  2  3  4  5  6  7  8  9 10 ``` | ```         cat {input.syns} | awk '{{print $10,$12,$13,$14,$16,$17,$1}}' | sed 's/ /	/g' | bedtools intersect -a stdin -b {input.gencov} -wa -wb | awk '{{if($10-$9=="5000") print $8,$9,$10,$7,$1,$2,$3,$4,$5,$6}}' | sed 's/ /	/g' | sed 's/	/STARTCOORD/' | sed 's/	/ENDCOORD/' > {output.windows}          sort -r -n -k2 < {output.windows} | awk '!x[$1]++' | sort -k1 | sed 's/STARTCOORD/	/' | sed 's/ENDCOORD/	/' > {params.absBestMatch}          cat {params.absBestMatch} | cut -f 8-10 | sort | uniq -c | awk '$1<=2 {{print}}' | awk '{{print $2,$3,$4}}' | sed 's/ /	/g' | sort | uniq | bedtools sort > {params.okWindows}          bedtools intersect -a <(<{params.absBestMatch} awk '{{print $8,$9,$10,$0}}' | sed 's/ /	/g' | sort -k1,1 | bedtools sort) -b {params.okWindows} -f 1 -r -wa | cut -f 4- | awk '$4>{params.match_bp} {{print}}'> {params.absBestMatchFilter}          echo "DONE" > {params.absLog} ``` | | |

##### Rule maf\_convert\_syns

×

Rule properties

|  |  |
| --- | --- |
| Jobs | 1 |
| Input files | |
| - results/Alouatta\_palliata/synteny\_lastal/HS/HS\_align | |
| Output files | |
| - results/Alouatta\_palliata/synteny\_lastal/HS/HS\_align\_converted | |
| Conda software stack | |
| - last=1238 - parallel=20210422 | |
| Code | |
| |  |  | | --- | --- | | ``` 1 2 ``` | ```         maf-convert psl {input} > {output} ``` | | |

##### Rule lastal\_syns

×

Rule properties

|  |  |
| --- | --- |
| Jobs | 1 |
| Input files | |
| - ../data/external\_raw/genome/AloPal\_v1.fasta - results/lastdb/lastdb\_Homo\_sapiens.GRCh38.dna\_rm.toplevel.noY.log | |
| Output files | |
| - results/Alouatta\_palliata/synteny\_lastal/HS/HS\_align | |
| Conda software stack | |
| - last=1238 - parallel=20210422 | |
| Code | |
| |  |  | | --- | --- | | ``` 1 2 ``` | ```         parallel-fasta "lastal -P {threads} {params.db}  | last-split" < {input.ref} > {output} ``` | | |

##### Rule lastdb

×

Rule properties

|  |  |
| --- | --- |
| Jobs | 1 |
| Input files | |
| - ../data/external\_raw/genome/Homo\_sapiens.GRCh38.dna\_rm.toplevel.noY.fasta | |
| Output files | |
| - results/lastdb/lastdb\_Homo\_sapiens.GRCh38.dna\_rm.toplevel.noY.log | |
| Conda software stack | |
| - last=1238 - parallel=20210422 | |
| Code | |
| |  |  | | --- | --- | | ``` 1 2 3 4 5 ``` | ```         lastdb -cR11 -P {threads} {params.db_name} {input}         mv {params.db_name}* {params.synteny_dir} #        touch {output}         echo "DONE" > {output.log} ``` | | |

##### Rule gencov\_bedtools

×

Rule properties

|  |  |
| --- | --- |
| Jobs | 3 |
| Input files | |
| - results/ref/AloPal\_v1/dedup/SRR9655170\_\_heterogametic.sorted.dedup.mismatch.{ED}.bam - results/ref/AloPal\_v1/dedup/SRR9655171\_\_heterogametic.sorted.dedup.mismatch.{ED}.bam - results/ref/AloPal\_v1/dedup/SRR9655168\_\_homogametic.sorted.dedup.mismatch.{ED}.bam - results/ref/AloPal\_v1/dedup/SRR9655169\_\_homogametic.sorted.dedup.mismatch.{ED}.bam - results/ref/AloPal\_v1/dedup/SRR9655170\_\_heterogametic.sorted.dedup.mismatch.{ED}.bam.bai - results/ref/AloPal\_v1/dedup/SRR9655171\_\_heterogametic.sorted.dedup.mismatch.{ED}.bam.bai - results/ref/AloPal\_v1/dedup/SRR9655168\_\_homogametic.sorted.dedup.mismatch.{ED}.bam.bai - results/ref/AloPal\_v1/dedup/SRR9655169\_\_homogametic.sorted.dedup.mismatch.{ED}.bam.bai - results/Alouatta\_palliata/coverage/genome\_5kb\_windows.out | |
| Output files | |
| - results/Alouatta\_palliata/coverage/gencov.mismatch.{ED}.out | |
| Conda software stack | |
| - bedtools=2.29.0 | |
| Code | |
| |  |  | | --- | --- | | ``` 1 2 ``` | ```         bedtools multicov -bams {input.bam_hetero} {input.bam_homo} -bed {input.bed} -p -q 20 > {output} ``` | | |

##### Rule bamtools\_filter

×

Rule properties

|  |  |
| --- | --- |
| Jobs | 8 |
| Input files | |
| - results/ref/AloPal\_v1/dedup/{sample}\_\_{group}.sorted.dedup.mismatch.unfiltered.bam | |
| Output files | |
| - results/ref/AloPal\_v1/dedup/{sample,SRR9655168|SRR9655169|SRR9655170|SRR9655171}\_\_{group,homogametic|homogametic|heterogametic|heterogametic}.sorted.dedup.mismatch.0.{ED, [0-9]+}.bam | |
| Conda software stack | |
| - bamtools ==2.5.1 | |
| Code | |
| |  |  | | --- | --- | | ```  1  2  3  4  5  6  7  8  9 10 11 12 13 14 15 16 17 18 19 20 21 22 23 24 25 26 27 28 29 30 31 32 33 34 35 36 37 38 39 40 41 42 43 44 45 46 47 48 ``` | ``` __author__ = "Antonie Vietor" __copyright__ = "Copyright 2020, Antonie Vietor" __email__ = "antonie.v@gmx.de" __license__ = "MIT"  from snakemake.shell import shell  log = snakemake.log_fmt_shell(stdout=False, stderr=True)  # extract arguments params = "" extra_limits = "" tags = snakemake.params.get("tags") min_size = snakemake.params.get("min_size") max_size = snakemake.params.get("max_size") min_length = snakemake.params.get("min_length") max_length = snakemake.params.get("max_length") additional_params = snakemake.params.get("additional_params")  if tags and tags is not None:     params = params + " " + " ".join(map('-tag "{}"'.format, tags))  if min_size and min_size is not None:     params = params + ' -insertSize ">=' + min_size + '"'     if max_size and max_size is not None:         extra_limits = extra_limits + ' -insertSize "<=' + max_size + '"' else:     if max_size and max_size is not None:         params = params + ' -insertSize "<=' + max_size + '"'  if min_length and min_length is not None:     params = params + ' -length ">=' + min_length + '"'     if max_length and max_length is not None:         extra_limits = extra_limits + ' -length "<=' + max_length + '"' else:     if max_length and max_length is not None:         params = params + ' -length "<=' + max_length + '"'  if additional_params and additional_params is not None:     params = params + " " + additional_params  if extra_limits:     params = params + " | bamtools filter" + extra_limits  shell(     "(bamtools filter"     " -in {snakemake.input[0]}" + params + " -out {snakemake.output[0]}) {log}" ) ``` | | |

##### Rule mark\_duplicates

×

Rule properties

|  |  |
| --- | --- |
| Jobs | 4 |
| Input files | |
| - results/ref/AloPal\_v1/mapped/{sample}\_\_{group}.sorted.bam | |
| Output files | |
| - results/ref/AloPal\_v1/dedup/{sample,SRR9655168|SRR9655169|SRR9655170|SRR9655171}\_\_{group,homogametic|homogametic|heterogametic|heterogametic}.sorted.dedup.mismatch.unfiltered.bam - results/Alouatta\_palliata/qc/dedup/{sample,SRR9655168|SRR9655169|SRR9655170|SRR9655171}\_\_{group,homogametic|homogametic|heterogametic|heterogametic}.metrics.txt | |
| Conda software stack | |
| - picard ==2.22.1 - snakemake-wrapper-utils ==0.1.3 | |
| Code | |
| |  |  | | --- | --- | | ```  1  2  3  4  5  6  7  8  9 10 11 12 13 14 15 16 17 18 19 20 21 22 23 24 25 26 27 ``` | ``` __author__ = "Johannes Köster" __copyright__ = "Copyright 2016, Johannes Köster" __email__ = "koester@jimmy.harvard.edu" __license__ = "MIT"   from snakemake.shell import shell from snakemake_wrapper_utils.java import get_java_opts  log = snakemake.log_fmt_shell(stdout=True, stderr=True)  extra = snakemake.params.get("extra", "") java_opts = get_java_opts(snakemake) bams = snakemake.input if isinstance(bams, str):     bams = [bams] bams = list(map("INPUT={}".format, bams))  shell(     "picard MarkDuplicates "  # Tool and its subcommand     "{java_opts} "  # Automatic java option     "{extra} "  # User defined parmeters     "{bams} "  # Input bam(s)     "OUTPUT={snakemake.output.bam} "  # Output bam     "METRICS_FILE={snakemake.output.metrics} "  # Output metrics     "{log}"  # Logging ) ``` | | |

##### Rule map\_reads

×

Rule properties

|  |  |
| --- | --- |
| Jobs | 4 |
| Input files | |
| - results/ref/AloPal\_v1/mapped/{sample}\_\_{group}.sorted.tmp.bam | |
| Output files | |
| - results/ref/AloPal\_v1/mapped/{sample,SRR9655168|SRR9655169|SRR9655170|SRR9655171}\_\_{group,homogametic|homogametic|heterogametic|heterogametic}.sorted.bam | |
| Conda software stack | |
| - bwa ==0.7.17 - samtools ==1.12 - picard ==2.20.1 | |
| Code | |
| |  |  | | --- | --- | | ```  1  2  3  4  5  6  7  8  9 10 11 12 13 14 15 16 17 ``` | ``` __author__ = "Johannes Köster" __copyright__ = "Copyright 2016, Johannes Köster" __email__ = "koester@jimmy.harvard.edu" __license__ = "MIT"   from snakemake.shell import shell from snakemake_wrapper_utils.samtools import get_samtools_opts   samtools_opts = get_samtools_opts(snakemake) log = snakemake.log_fmt_shell(stdout=True, stderr=True, append=True)   shell(     "samtools view {snakemake.params.extra} {samtools_opts} -o {snakemake.output[0]} {snakemake.input[0]} {log}" ) ``` | | |

##### Rule bwa\_index

×

Rule properties

|  |  |
| --- | --- |
| Jobs | 1 |
| Input files | |
| - ../data/external\_raw/genome/AloPal\_v1.fasta | |
| Output files | |
| - ../data/external\_raw/genome/AloPal\_v1.fasta.amb - ../data/external\_raw/genome/AloPal\_v1.fasta.ann - ../data/external\_raw/genome/AloPal\_v1.fasta.bwt - ../data/external\_raw/genome/AloPal\_v1.fasta.pac - ../data/external\_raw/genome/AloPal\_v1.fasta.sa | |
| Conda software stack | |
| - bwa ==0.7.17 | |
| Code | |
| |  |  | | --- | --- | | ```  1  2  3  4  5  6  7  8  9 10 11 12 13 14 15 16 17 18 19 20 21 22 23 24 25 26 27 28 29 30 31 32 ``` | ``` __author__ = "Patrik Smeds" __copyright__ = "Copyright 2016, Patrik Smeds" __email__ = "patrik.smeds@gmail.com" __license__ = "MIT"  from os import path  from snakemake.shell import shell  log = snakemake.log_fmt_shell(stdout=False, stderr=True)  # Check inputs/arguments. if len(snakemake.input) == 0:     raise ValueError("A reference genome has to be provided!") elif len(snakemake.input) > 1:     raise ValueError("Only one reference genome can be inputed!")  # Prefix that should be used for the database prefix = snakemake.params.get("prefix", "")  if len(prefix) > 0:     prefix = "-p " + prefix  # Contrunction algorithm that will be used to build the database, default is bwtsw construction_algorithm = snakemake.params.get("algorithm", "")  if len(construction_algorithm) != 0:     construction_algorithm = "-a " + construction_algorithm  shell(     "bwa index" " {prefix}" " {construction_algorithm}" " {snakemake.input[0]}" " {log}" ) ``` | | |

##### Rule samtools\_index

×

Rule properties

|  |  |
| --- | --- |
| Jobs | 12 |
| Input files | |
| - results/ref/AloPal\_v1/dedup/{sample}\_\_{group}.sorted.dedup.mismatch.{ED}.bam | |
| Output files | |
| - results/ref/AloPal\_v1/dedup/{sample,SRR9655168|SRR9655169|SRR9655170|SRR9655171}\_\_{group,homogametic|homogametic|heterogametic|heterogametic}.sorted.dedup.mismatch.{ED}.bam.bai | |
| Conda software stack | |
| - samtools ==1.10 | |
| Code | |
| |  |  | | --- | --- | | ```  1  2  3  4  5  6  7  8  9 10 11 12 13 ``` | ``` __author__ = "Johannes Köster" __copyright__ = "Copyright 2016, Johannes Köster" __email__ = "koester@jimmy.harvard.edu" __license__ = "MIT"   from snakemake.shell import shell  log = snakemake.log_fmt_shell(stdout=True, stderr=True)  shell(     "samtools index {snakemake.params} {snakemake.input[0]} {snakemake.output[0]} {log}" ) ``` | | |

##### Rule normalize\_cov\_mean

×

Rule properties

|  |  |
| --- | --- |
| Jobs | 3 |
| Input files | |
| - results/Alouatta\_palliata/coverage/gencov.mismatch.{ED}.out | |
| Output files | |
| - results/Alouatta\_palliata/coverage/gencov.mismatch.{ED}.norm.sexAverage.out | |
| Conda software stack | |
| - gawk=5.1.0 - numpy=1.19.2 - pandas=1.2.4 - scipy=1.6.3 - bedtools=2.29.0 | |
| Code | |
| |  |  | | --- | --- | | ``` 1 2 ``` | ```         python3 workflow/scripts/normalize_genCov.py {input} no-synteny {params.hetero} {params.homo} > {output} ``` | | |

##### Rule matchScaffold2Chr\_snp

×

Rule properties

|  |  |
| --- | --- |
| Jobs | 1 |
| Input files | |
| - results/Alouatta\_palliata/synteny\_lastal/HS/bestMatch.list - results/Alouatta\_palliata/variant\_calling/AloPal\_v1.heterozygosity.5kb.windows.NR.bed - results/Alouatta\_palliata/variant\_calling/AloPal\_v1.heterozygosity.sexAverage.NR.bed | |
| Output files | |
| - results/Alouatta\_palliata/synteny\_lastal/HS/heterozygosity.bestMatch - results/Alouatta\_palliata/synteny\_lastal/HS/heterozygosity.bestMatch.small - results/Alouatta\_palliata/synteny\_lastal/HS/heterozygosity.bestMatch.sexAverage.bed - results/Alouatta\_palliata/synteny\_lastal/HS/heterozygosity.bestMatch.small.sexAverage.bed | |
| Conda software stack | |
| - bedtools=2.29.0 | |
| Code | |
| |  |  | | --- | --- | | ``` 1 2 3 4 5 6 7 8 ``` | ```         bedtools intersect -a {input.bestMatch} -b {input.het} -wa -wb > {output.bestMatch}          cut -f 8,9,10,14- {output.bestMatch} > {output.bestMatch_small}                  bedtools intersect -a {input.bestMatch} -b {input.het_sexAverage} -wa -wb > {output.bestMatch_sexAverage}          cut -f 8,9,10,14- {output.bestMatch_sexAverage} > {output.bestMatch_small_sexAverage} ``` | | |

##### Rule proportion\_heterozygosity\_window\_2

×

Rule properties

|  |  |
| --- | --- |
| Jobs | 1 |
| Input files | |
| - results/Alouatta\_palliata/variant\_calling/AloPal\_v1.heterozygosity.5kb.windows.bed | |
| Output files | |
| - results/Alouatta\_palliata/variant\_calling/AloPal\_v1.heterozygosity.5kb.windows.NR.bed - results/Alouatta\_palliata/variant\_calling/AloPal\_v1.heterozygosity.sexAverage.NR.bed | |
| Conda software stack | |
| - gawk=5.1.0 - numpy=1.19.2 - pandas=1.2.4 - scipy=1.6.3 - bedtools=2.29.0 | |
| Code | |
| |  |  | | --- | --- | | ``` 1 2 3 4 ``` | ```         workflow/scripts/sum_heterozygosity_per_5kb.sh {input} > {output.het_sorted_window_mean} 	             python3 workflow/scripts/mean_heterozygosity_per_sex.py {output.het_sorted_window_mean} no-synteny {params.hetero} {params.homo} > {output.het_sexAverage} ``` | | |

##### Rule proportion\_heterozygosity\_window

×

Rule properties

|  |  |
| --- | --- |
| Jobs | 1 |
| Input files | |
| - results/Alouatta\_palliata/variant\_calling/AloPal\_v1.heterozygosity.bed - results/Alouatta\_palliata/coverage/genome\_5kb\_windows.out | |
| Output files | |
| - results/Alouatta\_palliata/variant\_calling/genome\_5kb\_windows.out - results/Alouatta\_palliata/variant\_calling/AloPal\_v1.heterozygosity.5kb.windows.tmp - results/Alouatta\_palliata/variant\_calling/AloPal\_v1.heterozygosity.5kb.windows.bed | |
| Conda software stack | |
| - bedtools=2.29.0 | |
| Code | |
| |  |  | | --- | --- | | ``` 1 2 3 4 5 6 ``` | ```         bedtools sort -i {input.windows} > {output.windows_sorted}         split -l 100000 {input.het} {params.split}         ls {params.dir} | grep split_file | while read file ; do bedtools intersect -a {input.windows} -b {params.dir}/${{file}} -wa -wb | cut -f 1-3,7- ; done > {output.het_tmp_window}          sort -k1,1 -k2,2 {output.het_tmp_window} > {output.het_sorted_window}         rm {params.split}* ``` | | |

##### Rule proportion\_heterozygosity

×

Rule properties

|  |  |
| --- | --- |
| Jobs | 1 |
| Input files | |
| - results/Alouatta\_palliata/variant\_calling/AloPal\_v1.biallelic.minQ20.minDP3.vcf.gz | |
| Output files | |
| - results/Alouatta\_palliata/variant\_calling/AloPal\_v1.heterozygosity.bed | |
| Code | |
| |  |  | | --- | --- | | ``` 1 2 ``` | ```         python3 workflow/scripts/heterozygosity_per_indv.py {input} {output.het} {params.hetero} {params.homo} > {log} ``` | | |

##### Rule vcftools\_filter

×

Rule properties

|  |  |
| --- | --- |
| Jobs | 1 |
| Input files | |
| - results/Alouatta\_palliata/variant\_calling/AloPal\_v1.vcf.gz | |
| Output files | |
| - results/Alouatta\_palliata/variant\_calling/AloPal\_v1.biallelic.minQ20.minDP3.vcf - results/Alouatta\_palliata/variant\_calling/AloPal\_v1.biallelic.minQ20.minDP3.vcf.gz | |
| Conda software stack | |
| - vcftools=0.1.16 - htslib=1.10 - tabix=0.2.6 - perl-vcftools-vcf=0.1.16 | |
| Code | |
| |  |  | | --- | --- | | ``` 1 2 3 4 ``` | ```         vcftools --gzvcf {input} --min-alleles 2 --max-alleles 2 --remove-filtered-geno-all --minQ 20 --minDP 3 --recode --stdout > {output.vcf}         bgzip -c {output.vcf} > {output.gz}         tabix -p vcf {output.gz} ``` | | |

##### Rule bgzip\_tabix

×

Rule properties

|  |  |
| --- | --- |
| Jobs | 1 |
| Input files | |
| - results/Alouatta\_palliata/variant\_calling/AloPal\_v1.vcf - results/Alouatta\_palliata/variant\_calling/freebayes\_done.log | |
| Output files | |
| - results/Alouatta\_palliata/variant\_calling/AloPal\_v1.vcf.gz | |
| Conda software stack | |
| - vcftools=0.1.16 - htslib=1.10 - tabix=0.2.6 - perl-vcftools-vcf=0.1.16 | |
| Code | |
| |  |  | | --- | --- | | ``` 1 2 3 ``` | ```         bgzip -c {input.vcf} > {output}         tabix -p vcf {output} ``` | | |

##### Rule platypus

×

Rule properties

|  |  |
| --- | --- |
| Jobs | 1 |
| Input files | |
| - ../data/external\_raw/genome/AloPal\_v1.fasta - results/ref/AloPal\_v1/dedup/SRR9655168\_\_homogametic.sorted.dedup.mismatch.unfiltered.bam - results/ref/AloPal\_v1/dedup/SRR9655169\_\_homogametic.sorted.dedup.mismatch.unfiltered.bam - results/ref/AloPal\_v1/dedup/SRR9655170\_\_heterogametic.sorted.dedup.mismatch.unfiltered.bam - results/ref/AloPal\_v1/dedup/SRR9655171\_\_heterogametic.sorted.dedup.mismatch.unfiltered.bam - results/ref/AloPal\_v1/dedup/SRR9655168\_\_homogametic.sorted.dedup.mismatch.unfiltered.bam.bai - results/ref/AloPal\_v1/dedup/SRR9655169\_\_homogametic.sorted.dedup.mismatch.unfiltered.bam.bai - results/ref/AloPal\_v1/dedup/SRR9655170\_\_heterogametic.sorted.dedup.mismatch.unfiltered.bam.bai - results/ref/AloPal\_v1/dedup/SRR9655171\_\_heterogametic.sorted.dedup.mismatch.unfiltered.bam.bai | |
| Output files | |
| - results/Alouatta\_palliata/variant\_calling/AloPal\_v1.vcf - results/Alouatta\_palliata/variant\_calling/freebayes\_done.log | |
| Conda software stack | |
| - platypus-variant=0.8.1.1 | |
| Code | |
| |  |  | | --- | --- | | ``` 1 2 3 ``` | ```         platypus callVariants --bamFiles={params.files} --refFile={input.ref} --output={output.vcf} --nCPU={threads}         echo "DONE" > {output.log} ``` | | |

##### Rule samtools\_stats

×

Rule properties

|  |  |
| --- | --- |
| Jobs | 12 |
| Input files | |
| - results/ref/AloPal\_v1/dedup/{sample}\_\_{group}.sorted.dedup.mismatch.{ED}.bam | |
| Output files | |
| - results/ref/AloPal\_v1/dedup/{sample,SRR9655168|SRR9655169|SRR9655170|SRR9655171}\_\_{group,homogametic|homogametic|heterogametic|heterogametic}.sorted.dedup.mismatch.{ED}.samtools.stats.txt | |
| Conda software stack | |
| - samtools ==1.10 | |
| Code | |
| |  |  | | --- | --- | | ```  1  2  3  4  5  6  7  8  9 10 11 12 13 14 15 16 17 ``` | ``` """Snakemake wrapper for trimming paired-end reads using cutadapt."""  __author__ = "Julian de Ruiter" __copyright__ = "Copyright 2017, Julian de Ruiter" __email__ = "julianderuiter@gmail.com" __license__ = "MIT"   from snakemake.shell import shell   extra = snakemake.params.get("extra", "") region = snakemake.params.get("region", "") log = snakemake.log_fmt_shell(stdout=False, stderr=True)   shell("samtools stats {extra} {snakemake.input} {region} > {snakemake.output} {log}") ``` | | |

##### Rule plotting\_chr

×

Rule properties

|  |  |
| --- | --- |
| Jobs | 1 |
| Input files | |
| - results/Alouatta\_palliata/output/synteny/HS/tables/diffGenomeCoverage.mismatch.0.0.chr.out - results/Alouatta\_palliata/output/synteny/HS/tables/diffGenomeCoverage.mismatch.0.2.chr.out - results/Alouatta\_palliata/output/synteny/HS/tables/diffGenomeCoverage.mismatch.unfiltered.chr.out - results/Alouatta\_palliata/output/synteny/HS/tables/diffHeterozygosity.chr.out | |
| Output files | |
| - results/Alouatta\_palliata/output/synteny/HS/plots/3\_sexDifferences.chromosome.pdf - results/Alouatta\_palliata/output/synteny/HS/plots/.misc/plotting\_chr.done | |
| Conda software stack | |
| - r-doby=4.6.6 - r-data.table=1.14.0 - r-ggplot2=3.3.3 - r-plot3d=1.3 - r-cowplot=1.1.1 - r-viridislite=0.4.0 - r-gridGraphics=0.5\_1 - r-plotly=4.9.3 - tk=8.6.10 - r-tcltk2=1.2\_11 - imagemagick=7.0.11\_14 - r-tidyverse=1.2.1 - r-ggextra=0.8 - r-ggpubr=0.4.0 | |
| Code | |
| |  |  | | --- | --- | | ``` 1 2 ``` | ```         Rscript workflow/scripts/scatterplot_chr.R {input.cov} {input.snp} {output.out_scatter2D} {params.chromosomes} {params.ED} 2> {log} ``` | | |

##### Rule calculate\_ratio\_chr

×

Rule properties

|  |  |
| --- | --- |
| Jobs | 3 |
| Input files | |
| - results/Alouatta\_palliata/synteny\_lastal/HS/gencov.mismatch.{ED}.norm.sexAverage.small.out | |
| Output files | |
| - results/Alouatta\_palliata/output/synteny/HS/tables/diffGenomeCoverage.mismatch.{ED}.chr.out | |
| Conda software stack | |
| - r-doby=4.6.6 - r-data.table=1.14.0 - r-ggplot2=3.3.3 - r-plot3d=1.3 - r-cowplot=1.1.1 - r-viridislite=0.4.0 - r-gridGraphics=0.5\_1 - r-plotly=4.9.3 - tk=8.6.10 - r-tcltk2=1.2\_11 - imagemagick=7.0.11\_14 - r-tidyverse=1.2.1 - r-ggextra=0.8 - r-ggpubr=0.4.0 | |
| Code | |
| |  |  | | --- | --- | | ``` 1 2 ``` | ```         Rscript workflow/scripts/calculate_chr.R {input} {output} ``` | | |

##### Rule calculate\_heterozygosity\_chr

×

Rule properties

|  |  |
| --- | --- |
| Jobs | 1 |
| Input files | |
| - results/Alouatta\_palliata/synteny\_lastal/HS/heterozygosity.bestMatch.small.sexAverage.bed | |
| Output files | |
| - results/Alouatta\_palliata/output/synteny/HS/tables/diffHeterozygosity.chr.out | |
| Conda software stack | |
| - r-doby=4.6.6 - r-data.table=1.14.0 - r-ggplot2=3.3.3 - r-plot3d=1.3 - r-cowplot=1.1.1 - r-viridislite=0.4.0 - r-gridGraphics=0.5\_1 - r-plotly=4.9.3 - tk=8.6.10 - r-tcltk2=1.2\_11 - imagemagick=7.0.11\_14 - r-tidyverse=1.2.1 - r-ggextra=0.8 - r-ggpubr=0.4.0 | |
| Code | |
| |  |  | | --- | --- | | ``` 1 2 ``` | ```         Rscript workflow/scripts/calculate_chr.R {input} {output} ``` | | |

##### Rule plotting\_linear

×

Rule properties

|  |  |
| --- | --- |
| Jobs | 1 |
| Input files | |
| - results/Alouatta\_palliata/output/synteny/HS/tables/diffGenomeCoverage.mismatch.0.0.{bp}bp.out - results/Alouatta\_palliata/output/synteny/HS/tables/diffGenomeCoverage.mismatch.0.2.{bp}bp.out - results/Alouatta\_palliata/output/synteny/HS/tables/diffGenomeCoverage.mismatch.unfiltered.{bp}bp.out - results/Alouatta\_palliata/output/synteny/HS/tables/diffHeterozygosity.{bp}bp.out | |
| Output files | |
| - results/Alouatta\_palliata/output/synteny/HS/plots/2\_sexesSeparate.genomeWide.{bp}bp.window.pdf - results/Alouatta\_palliata/output/synteny/HS/plots/1\_sexDifferences.genomeWide.{bp}bp.window.pdf - results/Alouatta\_palliata/output/synteny/HS/plots/.misc/plotting.linear.{bp}bp.done - results/Alouatta\_palliata/output/synteny/HS/tables/diffGenomeCoverage.mismatch.0.0.{bp}bp.outlier.out - results/Alouatta\_palliata/output/synteny/HS/tables/diffGenomeCoverage.mismatch.0.2.{bp}bp.outlier.out - results/Alouatta\_palliata/output/synteny/HS/tables/diffGenomeCoverage.mismatch.unfiltered.{bp}bp.outlier.out - results/Alouatta\_palliata/output/synteny/HS/tables/diffHeterozygosity.{bp}bp.outlier.out | |
| Conda software stack | |
| - r-doby=4.6.6 - r-data.table=1.14.0 - r-ggplot2=3.3.3 - r-plot3d=1.3 - r-cowplot=1.1.1 - r-viridislite=0.4.0 - r-gridGraphics=0.5\_1 - r-plotly=4.9.3 - tk=8.6.10 - r-tcltk2=1.2\_11 - imagemagick=7.0.11\_14 - r-tidyverse=1.2.1 - r-ggextra=0.8 - r-ggpubr=0.4.0 | |
| Code | |
| |  |  | | --- | --- | | ``` 1 2 ``` | ```         Rscript workflow/scripts/plot_windows_linear.R {input.cov} {input.snp} {output.absolute_out} {output.diff_out} {params.chromosomes} {params.ED} {params.nr_chromosomes} {params.window} {output.outlier_cov} {output.outlier_snp} 2> {log} ``` | | |

##### Rule calculate\_ratio\_window

×

Rule properties

|  |  |
| --- | --- |
| Jobs | 3 |
| Input files | |
| - results/Alouatta\_palliata/synteny\_lastal/HS/gencov.mismatch.{ED}.norm.sexAverage.small.out | |
| Output files | |
| - results/Alouatta\_palliata/output/synteny/HS/tables/diffGenomeCoverage.mismatch.{ED}.{window}bp.out | |
| Conda software stack | |
| - r-doby=4.6.6 - r-data.table=1.14.0 - r-ggplot2=3.3.3 - r-plot3d=1.3 - r-cowplot=1.1.1 - r-viridislite=0.4.0 - r-gridGraphics=0.5\_1 - r-plotly=4.9.3 - tk=8.6.10 - r-tcltk2=1.2\_11 - imagemagick=7.0.11\_14 - r-tidyverse=1.2.1 - r-ggextra=0.8 - r-ggpubr=0.4.0 | |
| Code | |
| |  |  | | --- | --- | | ``` 1 2 ``` | ```         Rscript workflow/scripts/calculate_windows_userSpec.R {input} {output} {params} ``` | | |

##### Rule calculate\_heterozygosity\_window

×

Rule properties

|  |  |
| --- | --- |
| Jobs | 1 |
| Input files | |
| - results/Alouatta\_palliata/synteny\_lastal/HS/heterozygosity.bestMatch.small.sexAverage.bed | |
| Output files | |
| - results/Alouatta\_palliata/output/synteny/HS/tables/diffHeterozygosity.{window}bp.out | |
| Conda software stack | |
| - r-doby=4.6.6 - r-data.table=1.14.0 - r-ggplot2=3.3.3 - r-plot3d=1.3 - r-cowplot=1.1.1 - r-viridislite=0.4.0 - r-gridGraphics=0.5\_1 - r-plotly=4.9.3 - tk=8.6.10 - r-tcltk2=1.2\_11 - imagemagick=7.0.11\_14 - r-tidyverse=1.2.1 - r-ggextra=0.8 - r-ggpubr=0.4.0 | |
| Code | |
| |  |  | | --- | --- | | ``` 1 2 ``` | ```         Rscript workflow/scripts/calculate_windows_userSpec.R {input} {output} {params} ``` | | |

##### Rule plotting

×

Rule properties

|  |  |
| --- | --- |
| Jobs | 1 |
| Input files | |
| - results/Alouatta\_palliata/output/synteny/HS/tables/diffGenomeCoverage.mismatch.0.0.{bp}bp.out - results/Alouatta\_palliata/output/synteny/HS/tables/diffGenomeCoverage.mismatch.0.2.{bp}bp.out - results/Alouatta\_palliata/output/synteny/HS/tables/diffGenomeCoverage.mismatch.unfiltered.{bp}bp.out - results/Alouatta\_palliata/output/synteny/HS/tables/diffHeterozygosity.{bp}bp.out - results/Alouatta\_palliata/output/synteny/HS/highlight\_file.list | |
| Output files | |
| - results/Alouatta\_palliata/output/synteny/HS/plots/4\_sexDifferences.{bp}bp.window.pdf - results/Alouatta\_palliata/output/synteny/HS/plots/4\_sexDifferences.{bp}bp.window.highlight.pdf - results/Alouatta\_palliata/output/synteny/HS/plots/.misc/plotting.{bp}bp.done - results/Alouatta\_palliata/output/synteny/HS/tables/sexDifferences\_mean\_SD.{bp}bp.window.tsv | |
| Conda software stack | |
| - r-doby=4.6.6 - r-data.table=1.14.0 - r-ggplot2=3.3.3 - r-plot3d=1.3 - r-cowplot=1.1.1 - r-viridislite=0.4.0 - r-gridGraphics=0.5\_1 - r-plotly=4.9.3 - tk=8.6.10 - r-tcltk2=1.2\_11 - imagemagick=7.0.11\_14 - r-tidyverse=1.2.1 - r-ggextra=0.8 - r-ggpubr=0.4.0 | |
| Code | |
| |  |  | | --- | --- | | ``` 1 2 ``` | ```             Rscript workflow/scripts/plot_windows.R {input.cov} {input.snp} {output.out_scatter} {params.chromosomes} {input.chromosomes_highlight} {params.ED} {params.window} {output.table} 2> {log} ``` | | |

##### Rule highlight\_file

×

Rule properties

|  |  |
| --- | --- |
| Jobs | 1 |
| Output files | |
| - results/Alouatta\_palliata/output/synteny/HS/highlight\_file.list | |
| Code | |
| |  |  | | --- | --- | | ``` 1 2 3 ``` | ```         echo {params.highlight_chr} | tr " " " " > {output} ``` | | |

##### Rule table\_readme

×

Rule properties

|  |  |
| --- | --- |
| Jobs | 1 |
| Input files | |
| - workflow/report/output\_table\_README.md | |
| Output files | |
| - results/Alouatta\_palliata/output/synteny/HS/tables/output\_table\_README.md | |
| Code | |
| |  |  | | --- | --- | | ``` 1 2 ``` | ```         cp {input} {output} ``` | | |

##### Rule run\_assembly\_stats

×

Rule properties

|  |  |
| --- | --- |
| Jobs | 1 |
| Input files | |
| - ../data/external\_raw/genome/AloPal\_v1.fasta | |
| Output files | |
| - results/Alouatta\_palliata/qc/assembly\_stats/AloPal\_v1\_stats.txt | |
| Conda software stack | |
| - assembly-stats=1.0 | |
| Code | |
| |  |  | | --- | --- | | ```  1  2  3  4  5  6  7  8  9 10 11 12 13 14 15 16 17 ``` | ``` __author__ = "Max Cummins" __copyright__ = "Copyright 2021, Max Cummins" __email__ = "max.l.cummins@gmail.com" __license__ = "MIT"  from snakemake.shell import shell from os import path  log = snakemake.log_fmt_shell(stdout=False, stderr=True)  shell(     "assembly-stats"     " {snakemake.params.extra}"     " {snakemake.input.assembly}"     " > {snakemake.output.assembly_stats}"     " {log}" ) ``` | | |

##### Rule synteny\_stats

×

Rule properties

|  |  |
| --- | --- |
| Jobs | 1 |
| Input files | |
| - results/Alouatta\_palliata/synteny\_lastal/HS/bestMatch.list - results/Alouatta\_palliata/coverage/genome\_5kb\_windows.out - results/Alouatta\_palliata/qc/assembly\_stats/AloPal\_v1\_stats.txt | |
| Output files | |
| - results/Alouatta\_palliata/synteny\_lastal/HS/synteny\_stats.out | |
| Code | |
| |  |  | | --- | --- | | ```  1  2  3  4  5  6  7  8  9 10 ``` | ```         ref_length=$(cat {input.ref_stats} | cut -f 2 | tail -n 1)          window_length=$(cat {input.windows} | cut -f 2-3 | awk '{{print $2-$1}}' | paste -sd+ - | bc)          match_bp=$(cat {input.bestMatch} | cut -f 1-3 | sort | uniq | awk '{{print $3-$2}}' | paste -sd+ - | bc )         echo "Study-species reference genome: {params.ref_genome_name}" > {output}         echo "Synteny-species reference genome: {params.synteny_ref}" >> {output}         echo "Total length of study-species reference genome: $ref_length bp" >> {output}         echo "scale=2; ${{match_bp}}/${{ref_length}}" | bc | awk '{{printf "%f",  $0}}' | awk '{{print "Prop. of synteny-species reference genome length matched to synteny-species:", $0}}'>> {output}         echo "Total length of study-species reference genome 5kb windows (only scaffolds longer than {params.MIN_SIZE_SCAFFOLD} bp): $window_length bp" >> {output}         echo "scale=2; ${{match_bp}}/${{window_length}}" | bc | awk '{{printf "%f", $0}}' | awk '{{print "Prop. of synteny-species reference genome 5kb windows matched:",$0}}' >> {output} ``` | | |

##### Rule het\_calc

×

Rule properties

|  |  |
| --- | --- |
| Jobs | 1 |
| Input files | |
| - results/Alouatta\_palliata/variant\_calling/AloPal\_v1.biallelic.minQ20.minDP3.vcf.gz | |
| Output files | |
| - results/Alouatta\_palliata/variant\_calling/AloPal\_v1.biallelic.minQ20.minDP3.het | |
| Conda software stack | |
| - vcftools=0.1.16 - htslib=1.10 - tabix=0.2.6 - perl-vcftools-vcf=0.1.16 | |
| Code | |
| |  |  | | --- | --- | | ``` 1 2 ``` | ```         vcftools --gzvcf {input} --het --stdout > {output} ``` | | |

##### Rule het\_calc\_genome

×

Rule properties

|  |  |
| --- | --- |
| Jobs | 1 |
| Input files | |
| - results/Alouatta\_palliata/variant\_calling/AloPal\_v1.biallelic.minQ20.minDP3.het - results/Alouatta\_palliata/qc/assembly\_stats/AloPal\_v1\_stats.txt | |
| Output files | |
| - results/Alouatta\_palliata/variant\_calling/AloPal\_v1.heterozygosity.perc.csv | |
| Code | |
| |  |  | | --- | --- | | ``` 1 2 3 4 ``` | ```         length=$(cat {input.assembly_stats} | cut -f 2 | tail -n 1)          echo "Sample,Heterozygous_sites,Genome_length,Percentage_heterozygosity" > {output}         cat {input.het} | grep -v INDV | awk -v var="$length" '{{print $1,($4-$2),var,($4-$2)/var*100}}' | sed 's/ /,/g' >> {output} ``` | | |

##### Rule gencov\_prepare\_fasta

×

Rule properties

|  |  |
| --- | --- |
| Jobs | 1 |
| Input files | |
| - ../data/external\_raw/genome/AloPal\_v1.fasta.fai | |
| Output files | |
| - results/Alouatta\_palliata/coverage/AloPal\_v1.filter.10000.fasta.fai - results/Alouatta\_palliata/coverage/genome\_5kb\_windows.out | |
| Conda software stack | |
| - bedtools=2.29.0 | |
| Code | |
| |  |  | | --- | --- | | ``` 1 2 3 ``` | ``` 	    cat {input} | awk '$2>= {params} {{print $0}}' > {output.filter_fai}         bedtools makewindows -g {output.filter_fai} -w 5000 -s 5000 | awk '$3-$2==5000 {{print}}' | bedtools sort > {output.windows} ``` | | |
